# An efficient multilevel image thresholding method based on improved heap-based optimizer

**DOI:** 10.1038/s41598-023-36066-8

**Published:** 2023-06-05

**Authors:** Essam H. Houssein, Gaber M. Mohamed, Ibrahim A. Ibrahim, Yaser M. Wazery

**Affiliations:** Faculty of Computers and Information, Minia University, Minia, Egypt

**Keywords:** Computational models, Image processing

## Abstract

Image segmentation is the process of separating pixels of an image into multiple classes, enabling the analysis of objects in the image. Multilevel thresholding (MTH) is a method used to perform this task, and the problem is to obtain an optimal threshold that properly segments each image. Methods such as the Kapur entropy or the Otsu method, which can be used as objective functions to determine the optimal threshold, are efficient in determining the best threshold for bi-level thresholding; however, they are not effective for MTH due to their high computational cost. This paper integrates an efficient method for MTH image segmentation called the heap-based optimizer (HBO) with opposition-based learning termed improved heap-based optimizer (IHBO) to solve the problem of high computational cost for MTH and overcome the weaknesses of the original HBO. The IHBO was proposed to improve the convergence rate and local search efficiency of search agents of the basic HBO, the IHBO is applied to solve the problem of MTH using the Otsu and Kapur methods as objective functions. The performance of the IHBO-based method was evaluated on the CEC’2020 test suite and compared against seven well-known metaheuristic algorithms including the basic HBO, salp swarm algorithm, moth flame optimization, gray wolf optimization, sine cosine algorithm, harmony search optimization, and electromagnetism optimization. The experimental results revealed that the proposed IHBO algorithm outperformed the counterparts in terms of the fitness values as well as other performance indicators, such as the structural similarity index (SSIM), feature similarity index (FSIM), peak signal-to-noise ratio. Therefore, the IHBO algorithm was found to be superior to other segmentation methods for MTH image segmentation.

## Introduction

Segmentation has an important role in the field of image processing^1^. Segmentation is the process of separating an image into two or more homogeneous segments based on the characteristics of the pixels in the image. It is utilized in various scopes, such as industry and medicine^2^, agriculture^3^, and surveillance^4^. Thresholding is one of the most common image segmentation approaches. To define the thresholds, most methods use the histogram of the image^5^, which is vital for determining the probability distribution value of pixels in the image^6^. Thresholding obtains the information of the histogram from an image and determines the best threshold ((*th*)) for categorizing the pixels into various groups. Image thresholding approaches can be categorized into two types: multi-level and bi-level thresholding. Bi-level thresholding techniques use one threshold to separate an image into two groups, whereas multi-level thresholding (MTH) uses two or more thresholds to separate an image into many groups^1^.

To obtain the best threshold values in MTH segmentation, thresholding techniques can be classified into two approaches: non-parametric and parametric. In parametric techniques, each group of grayscale range should be consistent with a Gaussian distribution. Parametric approaches are dependent on the evaluation of the histogram using mathematical operations. The Gaussian mixture is widespread, where used to define the set of operations that convergent the histogram, and the best thresholds are then selected. Non-parametric approaches employ distinct methods to separate the pixels into homogeneous areas; then, the best threshold is defined using statistical information, such as entropy or variance. The Kapur method^7^ and Otsu method^8^ are used in this study. The Otsu method selects the best thresholds by the maximization of the variance among groups. In the Kapur method, the threshold value is defined by minimizing the cross entropy between a segmented image and the original image. These methods are efficient for one or two *th* values of thresholds. However, they have several restrictions; for example, they are very costly in computation, mostly when the number of thresholds increases. Non-parametric techniques have several advantages. Specifically, in terms of computation, these methods are computationally faster than parametric methods, especially when used in optimization problems. Metaheuristic algorithms (MAs) can be used in the search process. Generally, these algorithms provide better results than techniques dependent on thresholding methods^9,10^.

Metaheuristic algorithms are used to solve challenging real-world problems. In the past several decades, researchers have extensively demonstrated the ability of MAs to solve several types of difficult optimization problems in various areas, such as optimization^11^, communications^12^, bioinformatics^13^, drug design^14^, Image segmentation^15,16^ and feature selection^17^, mainly due to the fact that these algorithms are general-purpose and easy to implement^18^. MAs are commonly inspired by nature and can be classified into four main categories: Evolutionary-based, swarm-based, physics-based, and human-based algorithms. Evolutionary-based algorithms (use mechanisms inspired by biological evolution, such as recombination, crossover, mutation, and the heritage of features in offspring^19^. Candidate solutions to optimization problems are represented as individuals in a population, and the quality of the solutions is determined by the fitness function. Two main Evolutionary-based algorithms are differential evolution (DE)^20^ and the genetic algorithm (GA)^21^, which are inspired by biological evolution, while swarm-based algorithms mimic the mass behavior of living creatures. Living creatures interact with each other in nature to achieve optimal mass behavior^22^. An offshoot is particle swarm optimization (PSO)^23^, which mimics the hunting behavior of groups of fish and birds. Physics-based algorithms are generally inspired by physics to generate factors that enable search for the optimal solution in the search scope^24,25^. Some of the most common categories in this branch are the gravitational search algorithm (GSA)^26^ and electromagnetism optimization (EMO)^27^. Human-based algorithms are inspired by human gregarious demeanor. The common and recent used algorithms in this category are teaching–learning-based optimization (TLBO)^28^, and the heap-based optimizer (HBO)^29^.

With respect to MTH in image processing, it is possible to use thresholding approaches such as the Otsu or Kapur method^30^ as the objective function. The problem is not only concerned with the increased number of thresholds, but is also related to the image; for this reason, each image is an autonomous problem concerned with the levels of thresholding used for segmentation^31^. The optimal segmentation threshold values must be highly accurate in most processes. Therefore, the use of MAs has been expanded in this field. The moth swarm algorithm discussed in^32^ was used to obtain the best threshold values with the Kapur method based on previous literature. In addition, a modified firefly algorithm was proposed in^33^ for image processing, and used the Kapur and Otsu methods as objective functions. In^34^, ant colony optimization was used in image segmentation based on a multi-threshold image segmentation method with Kapur entropy and a non-local two-dimensional histogram. In^35^, the researchers used a novel concept called a hyper-heuristic with MTH image segmentation, in which each iteration determined the optimal execution sequence of MAs to determine the best threshold values.

In^10^, the black widow optimization algorithm^10^ was proposed to determine the optimal threshold using the Kapur or Otsu method as an objective function with a multi-level threshold. In^36^, the crow search algorithm was utilized in conjunction with the Kapur approach and 30th values to obtain the optimal threshold. In^37^, the authors proposed the efficient krill herd algorithm to determine the best thresholds at various levels for color images, where the Tsallis entropy, Otsu method, and Kapur entropy were utilized as fitness functions. Harris hawks optimization (HHO) is a new algorithm, and its hybridization was achieved by adding another powerful algorithm, the differential evolution (DE) algorithm^38^. Specifically, the entire population was split into two equal subpopulations, which were assigned to the HHO and DE algorithms, respectively. This hybridization used the Otsu and Kapur approaches as fitness functions. In^39^, the authors combined the classical Otsu’s method with an energy curve for applying the segmentation of colored images in multilevel thresholding. The water cycle algorithm (WCA) is integrated with Masi entropy (Masi-WCA) and Tsallis in^40^ to segment the color image. the results of the experiment proved the superiority of the WCA for multilevel thresholding with Masi entropy compared to other competitive algorithms. The authors in^41^ used a multi-verse optimizer (MVO) algorithm based on the Energy-MCE thresholding approach for searching the accurate and near-optimal thresholds for segmentation.

In the same context, Elaziz et al.^42^ proposed DE as a technique to select the best MAs to determine the optimal threshold for the Otsu method. Opposition-based learning (OBL)is one of the important effective methods to improve search efficiency of meta-heuristic algorithms^43^. The hyper-heuristic method based on a genetic algorithm was presented in^44^ and estimates various MAs for determining the optimal threshold for each image using a predetermined value of *th* using the Otsu method. In^45^, new efficient version of the recent chimp optimization algorithm (ChOA) was proposed to overcome the weaknesses of the original ChOA and called opposition-based Lévy Flight chimp optimizer (IChOA). The IChOA is applied to solve the problem of MTH using the Otsu and Kapur methods as objective functions. In this paper, several MAs, including SCA, MFO, SSA, and EMO, were combined with Otsu. As mentioned, the utilization of MAs in MTH is growing rapidly, and a summary of various approaches can be found in^46^.

According to the No Free Lunch theorem, this signifies that there is no ideal algorithm for a particular problem^47^. For this reason, any algorithm must be evaluated for a real problem to demonstrate its performance. MTH based on OBL are frequently used to solve a diversity of other optimization problems. Therefore, this paper seeks to further the research in the image segmentation field by utilizing the recent heap-based optimizer (HBO). The HBO was introduced in^29^ for optimization. This algorithm mimics the job responsibilities and descriptions of employees. The staff are coordinated in a hierarchy, and a nonlinear tree-shaped data structure is used to represent the heap. The benefit of these algorithms is that types with unsuitable fitness are deleted from the circle, leading to improved convergence speed. Based on the advantages of the HBO and the No Free Lunch theorem, this paper aims to present an alternative version from HBO called IHBO algorithm to discover the optimal solution of complex MTH problems and overcoming the weaknesses of the original HBO.

The proposed method for MTH based on the HBO is called IHBO, and applies the Kapur and Otsu methods individually to obtain the optimal threshold from benchmark images. IHBO explores the search area determined by a histogram technique to provide the best threshold values using a set of factors inspired by humans’ career hierarchy. The performance of IHBO is evaluated through various tests in which benchmark images are utilized with many levels of complexity. The segmentation results are estimated using various assessments, such as the structural similarity (SSIM) index^48^, feature similarity (FSIM) index^49^ and peak signal-to-noise ratio (PSNR)^50^. Furthermore, IHBO algorithm was evaluated on the CEC’2020 test suite and compared against seven well-known metaheuristic algorithms including the basic HBO^29^, SSA^51^, MFO^52^, GWO^53^, SCA^54^, HS^55^, and EMO^27^. The evaluations are executed through various non-parametric and statistical techniques to determine whether the optimal solutions provided by the IHBO are superior.

The main contributions of this paper can be summarized as follows:

- An efficient HBO based on OBL called IHBO to overcome the weaknesses of the original HBO is presented.
- Evaluating the effectiveness of IHBO on the CEC’2020 test suite.
- IHBO is proposed to solve the problem of high computational cost for MTH .
- Proving the ability of the IHBO to solve the image segmentation problems using the Kapur’s entropy and Otsu’s method as fitness function.
- Verify the image quality using set of metrics FSIM, PSNR and SSIM to obtain optimal solutions.
- Evaluating the performance of the provided method based on the various segmentation degrees to estimate stability of the optimizer and evaluate quality of the segmentation.

The remainder of this paper is organized as follows. “Preliminaries” section describes the materials and methods used in this study, while “The proposed IHBO algorithm” section  presents the proposed algorithm. “Environmental and experimental requirements” section  illustrates the environmental and experimental requirements, while “Experimental results and discussion” section presents the performance evaluation and experimental results. Finally, conclusions and proposals for future work are provided in “Conclusions and future works” section.

## Preliminaries

This section introduces the materials required to implement the proposed segmentation method, as well as the approaches implemented based on the above-mentioned approaches.

### Objective functions formulation

The entropy criterion of the Kapur^7^ approach and between-class variance of the Otsu^8^ approach are widely utilized to determine the optimal threshold value *th* in image segmentation. Both algorithms were developed for bi-level thresholding techniques. An approach can be readily extended for solving MTH problems.

#### Otsu method for segmentation

The Otsu method is an automatic and non-parametric technique used to determine the optimal thresholds of an image^8^. This method is based on the maximum variance of the various classes as a criterion to segment the image. The intensity levels *L* are taken from a grayscale image, and the equation below is used to calculate the probability distribution of the intensity value:

1 $$\begin{aligned} {{Ph_i} = \frac{{{n_i}}}{{nk}}, {Ph_i} \ge 0, \sum \limits _{i = 1}^{L} {{Ph_i} = 1}, } \end{aligned}$$

where *i* is a specific intensity level in the range $0 \le i \le L-1$ and $n_i$ is the number of gray level *i* appearing in the image. The number of pixels in the image is *nk* and $Ph_i$ is the probability distribution of the intensity levels. For the simplest segmentation (bi-level), two classes are represented as

2 $$\begin{aligned} {C_1} = \frac{{Ph_1}}{{\omega _0(th)}},\ldots ,\frac{{Ph_{th}}}{{\omega _0(th)}} \text { and } {C_2} = \frac{{Ph_{th + 1}^c}}{{\omega _1(th)}},\ldots ,\frac{{Ph_L}}{{\omega _1(th)}}\mathrm{{ }}. \end{aligned}$$

The probability distribution for $C_1$ and $C_2$ are $\omega _0(th)$ and $\omega _1(th)$, respectively, as illustrated in (3).

3 $$\begin{aligned} \omega _0(th)=\sum _{i=1}^{th}Ph_i \text { and } \omega _1(th)=\sum _{th+1}^{L}Ph_i. \end{aligned}$$

It is necessary to calculate the mean levels $\mu _0$ and $\mu _1$ that define the classes using (4). Once these values are calculated, the Otsu based between classes $\sigma _B^{2}$ is calculated using (5) as follows:

4 $$\begin{aligned} \mu _0= & {} \sum _{i=1}^{th} \dfrac{ iPh_i}{\omega _0(th)} \text { and } \mu _1=\sum _{i=th+1}^L \dfrac{iPh_i}{ \omega _1(th)}\end{aligned}$$

5 $$\begin{aligned} \sigma _B^{2}= & {} \sigma _1+\sigma _2 \end{aligned}$$

Moreover, $\sigma _1$ and $\sigma _2$ in (5) indicate the variance of regions $C_1$ and $C_2$, and are calculated as

6 $$\begin{aligned} \sigma _1=\omega _0 (\mu _0 +\mu _T)^2 \text { and } \sigma _2=\omega _1(\mu _1 +\mu _T)^2, \end{aligned}$$

where $\mu _T=\omega _0\mu _0+\omega _1\mu _1$ and $\omega _0+\omega _1=1$. Based on the values $\sigma _1$ and $\sigma _2$, (7) provides the fitness function. Subsequently, the optimization problem is reduced to determine the intensity level that maximizes (7):

7 $$\begin{aligned} F_{Otsu}(th)=Max(\sigma _B^{2}(th)) \text { where } 0 \le th \le L-1, \end{aligned}$$

where $\sigma _B^{2}(th)$ is the Otsu method variance for a given *th* value. EBO methods are used determine the intensity level *th* for maximizing the fitness function according to (7). The fitness or objective function $F_{Otsu}(th)$ can be modulated for MTH as follows:

8 $$\begin{aligned} F_{Otsu}(TH)=Max(\sigma _B^{2}(th)) \text { where } 0 \le th \le L-1 \text { and } i=[1,2,3,\ldots ,n], \end{aligned}$$

where $TH= [th_1,th_2,\ldots th_n-1]$ represents a vector including MTH, and the variance calculations are as illustrated in (9).

9 $$\begin{aligned} \sigma _B^{2}=\sum _{i=1}^n \sigma _i = \sum _{i=1}^n \omega _1(\mu _1 -\mu _T)^2. \end{aligned}$$

Here *i* represents a class, and $\omega _i$ is the occurrence probability, and $\mu _j$ is the mean of a class. For MTH, these values are obtained as

10 $$\begin{aligned} \omega _{n-1}(th)=\sum _{i=th_n +1}^L Ph_i \end{aligned}$$

and

11 $$\begin{aligned} \mu _{n-1}=\sum _{i=th_{n}+1}^L \dfrac{iPh_i}{\omega _1(th_n) }. \end{aligned}$$

#### Kapur entropy

Another non-parametric method used to determine the best threshold value of an image was proposed by Kapur in^7^. The approach determines the best (*th*) implying the overall entropy to be maximized. For a bi-level scenario, the Kapur target capacity can be determined as

12 $$\begin{aligned} F_{kapur}(th)=h_1 +h_2, \end{aligned}$$

where the entropies $H_1$ and $H_2$ are computed as follows:

13 $$\begin{aligned} h_1=\sum _{i=1}^{th} \dfrac{Ph_i}{\omega _0} ln\left( \frac{Ph_i}{\omega _0}\right) \text { and } h_2=\sum _{i=th+1}^{L} \dfrac{Ph_i}{\omega _1} ln\left( \frac{Ph_i}{\omega _1}\right) . \end{aligned}$$

In (13), $Ph_i$ is the probability distribution of the intensity levels, which is computed by (1), and $\omega _0(th)$ and $\omega _1(th)$ are the probability distributions of classes $C_1$ and $C_2$, respectively. *ln*(.) represents the natural logarithm. Like the Otsu method, the entropy-based method can be modulated for MTH values. In this case, it is necessary to separate an image into *n* groups using a similar number of thresholds. The equation below can define the new objective function:

14 $$\begin{aligned} F_{kapur}(TH) =\sum _{i=1}^n h_i, \end{aligned}$$

where $TH=[th_1,th_2,\ldots th_{n-1}]$ is the vector including MTH. Each entropy is computed separately with its respective *th* values; thus, (14) is expanded for *n* entropies as follows:

15 $$\begin{aligned} h_n^c=\sum _{i=th_{n+1}}^L \dfrac{Ph_i}{\omega _{n-1}} ln \left( \dfrac{Ph_i}{\omega _{n-1}}\right) . \end{aligned}$$

Therefore, the values of probability occurrence $(\omega _0^c , \omega _1,\ldots , \omega _{n-1})$ of *n* classes can be determined using (10) and the probability distribution $Ph_i$ in (1).

### Heap-based optimizer (HBO)

The HBO mimics the job responsibilities and descriptions of the employees within a company^29^. Although the job title differ from company to another and from business to another, they are organized in a hierarchy and many of titles are given like corporate hierarchy structure, organizational chart tree, or corporate rank hierarchy (CRH), etc. The collection of methods that outlines how particular activities are directed to realize the goals of an organization and also defines how information flows among levels within the company^56^ is called an organizational structure. In this section, we explain the mathematical model of the Heap-based optimizer.

#### Mathematical modeling of the interaction with immediate boss

The upper levels set the rules and laws for employees within the centralized structure and subordinates follow their immediate boss. By the assumption that each immediate boss is a parent node of its children, thus we can model this behaviour by upgrading the location of each search agent ${{\vec {x}}_{i}}$ with reference to its original node *B* by using the below equation:

16 $$\begin{aligned} X_i^k(t + 1) = {B^k} + \gamma {\lambda ^k}|{B^k} - X_i^k(t)|\ \end{aligned}$$

where *t* is the current iteration, and | | calculates the absolute value. $\lambda ^k$ is the $k^{th}$ component of vector ${\vec {\lambda }}$, and it is generated random as following

17 $$\begin{aligned} \vec {\lambda } =2r-1 \end{aligned}$$

where *r* is a random number in range $\left[ 0,1 \right]$. In Eq. (16), the designed parameter is $\gamma$, this parameter is computed by the following rule:

18 $$\begin{aligned} \gamma = \left| {2 - \frac{{\left( {t\bmod \frac{T}{c}} \right) }}{{\frac{T}{{4c}}}}} \right| \ \ \end{aligned}$$

The current iteration is *t*, *T* is the maximum iteration’s number, and *C* is a user defined parameter. while executing the iterations, $\gamma$ decrease linearly from 2 to 0 and when reach to 0, it will increase again to 2 with iterations.

#### Modeling the interaction between colleagues mathematically

The employees having the same position are considered to be colleagues. Each employee interact with others to achieve the goals of an organization. By assuming that the nodes at the same level in heap are colleagues and others are search agents ${{\vec {x}}_{i}}$and they update their position based on the position of others selected colleagues ${{\vec S}_r}$, the position of a search agent is calculated as follows:

19 $$\begin{aligned} X_i^k(t + 1) = \ {\left\{ \begin{array}{ll} S_r^k + {\gamma ^{{\lambda ^k}}}|S_r^k - x_i^k(t)|,f(\vec S_r) < f(\vec x_i(t)) \\ x_i^k + {\gamma ^{{\lambda ^k}}}|S_r^k - x_i^k(t)|, f(\vec S_r) \ge f(\vec x_i(t)) \end{array}\right. } \end{aligned}$$

where *f* is the objective function and calculates the fitness of each search agent. Equation (19) enables the search agents to explore the search space $S_r^k$ if $({{\vec S}_r}) < f({{\vec x}_i}(t))$ and allows to explore the search space $x_i^k$ otherwise.

#### Self contribution of an employee

This stage explains the concept of employees self contribution. Modeling of this behavior are executed by retaining the prior position of the employee in the next iteration, as illustrated in below equation:

20 $$\begin{aligned} x_i^k(t + 1) = x_i^k(t) \end{aligned}$$

In Eq. (20), the search agent ${{\vec x}_i}$ does not change its rank for it’s *k*th design parameter in the next iteration. We used this behavior to organize the rate of change of each search agent in population.

#### Putting it all together

This phase explains how to combine the equations of position updating and modelling in previous subsections in one equation. There are three probabilities of selection that are used to determine equation used in updating position of search agents, this probabilities of selection is used to switch between exploration and exploitation phase. These probabilities is divided into three proportions $p_1$, $p_2$, and $p_3$. The search agent updates its location using Eq. (20) according to the proportion $p_1$. The below equation computes the outlines of $p_1$.

21 $$\begin{aligned} {p_1} = 1 - \frac{t}{T} \end{aligned}$$

The current iteration *t*, *T* is the maximum number of iterations. The search agent updates its location using Eq. (16) according to the selection of proportion $p_2$. The below equation compute the outlines of $p_2$.

22 $$\begin{aligned} {p_2} = {p_1} + \frac{{1 - {p_1}}}{2} \end{aligned}$$

Finally, the search agent updates its location using Eq. (19) according to the selection of $p_3$. The below equation computes the outlines of $p_3$.

23 $$\begin{aligned} {p_3} = {p_2} + \frac{{1 - {p_1}}}{2} = 1 \end{aligned}$$

A general position updating mechanism of HBO is computed as follows:

24 $$\begin{aligned} x_i^k(t + 1) = \left\{ {\begin{array}{ll} x_i^k(t),&{}\quad p \le {p_1}\\ {B^k} + \gamma {\lambda ^k}\left| {{B^k} - x_i^k(t)} \right| , &{}\quad p> {p_1}\,and\,p \le {p_2}\\ S_r^k + \gamma {\lambda ^k}\left| {S_r^k - x_i^k(t)} \right| , &{}\quad p> {p_2}\,and\,p \le {p_3}\,and\,f({{\vec S}_r}) < f({{\vec x}_i}(t))\\ x_i^k + \gamma {\lambda ^k}\left| {S_r^k - x_i^k(t)} \right| , &{}\quad p > {p_2}\,and\,p \le {p_3}\,and\,f({{\vec S}_r}) \ge f({{\vec x}_i}(t)) \end{array}} \right. \end{aligned}$$

where $p_1$, $p_2$ and $p_3$ are random numbers inside range [0, 1]. This subsection clarifies that the Eq. (20) improves exploration phase, Eq. (16) improves exploitation phase and convergence, and Eq. (19) allows the search agent to move from the exploration phase to exploitation phase. According to this observations, $p_1$ is higher initially and decreases linearly over iterations, this decreases the exploration phase and improves exploitation phase with iterations. After calculating $p_1$, the remainder of the span is splitted into two equal portions, which makes attraction towards the colleague and boss equally probable.

#### Steps of HBO

This section summarizes the HBO steps and clarifies details about their implementation-related calculations.

- Parameters initialization and definition: At first, all the search agents are randomly initialized in a potential solution space. The minimum and maximum boundaries of the search space are defined by variables $(L_i,\ U_i)$ respectively. The number of the population is (*N*) and maximum number of iteration (*T*). The specific parameter *C* can be calculated from $C=\left\lfloor T/25 \right\rfloor$.
- Population initialization: The random population *P* is generated from *N* search agents, each consisting of *D* dimensions. The population’s representation *P* is shown as follows:
  $$\begin{aligned} p = \left[ {\begin{array}{*{20}{c}} {\vec x_1^T}\\ {\vec x_2^T}\\ \vdots \\ {\vec x_N^T} \end{array}} \right] = \left[ {\begin{array}{*{20}{c}} {x_1^1}&{}\quad {x_1^2}&{}\quad {x_1^3}&{}\quad {}&{}\quad {x_1^D}\\ {x_2^1}&{}\quad {x_2^2}&{}\quad {x_2^3}&{}\quad {}&{}\quad {x_2^D}\\ {}&{}\quad {}&{}\quad {}&{}\quad {}&{}\quad {}\\ {x_N^1}&{}\quad {x_N^2}&{}\quad {x_N^3}&{}\quad {}&{}\quad {x_N^D} \end{array}} \right] \end{aligned}$$
- Heap building: We utilize $3-ary$ heap to execute CRH. Although heap is a tree shaped data structure, it can be executed using an array. The below operations are $d-ary$ heap based operations required for the HBO execution.

1. parent (*i*): By the assumption that the heap is performed as an array, this method receives the node’s index then retrieves its parent’s index. The formulation of parent’s index for a node *i* is calculated by below equation:
  25 $$\begin{aligned} parent(i) = \left\lfloor {\frac{{i + 1}}{D}} \right\rfloor \end{aligned}$$
  where $\lfloor \rfloor$ indicates the floor function, which retrieves the highest integer less than or equal to a given input.
2. child (*i*; *k*): The node can own a maximum of 3 childrens in a $3-ary$ heap. Therefore we can say, the manager may not manages more than 3 direct persons. The index of the *k*th child of a node *i* is returned by this function. The below equation shows mathematical formulation of this function.
  26 $$\begin{aligned} child(i,k) = D \times i - D + k + 1 \end{aligned}$$
  For example,index of the 3nd child of 3nd node is calculated as:
  $$\begin{aligned} child(3,3) = 12 - 4 + 3 + 1 = 12 \end{aligned}$$
3. depth (*i*): Assuming the last level depth equals to 0, therefore we can calculate the depth of any node *i* in constant time through below formula:
  27 $$\begin{aligned} depth(i) = \left\lceil {\log (D \times i - i + 1)} \right\rceil - 1 \end{aligned}$$
  The ceil function is $\lceil \rceil$, which retrieves the smallest integer greater than or equal to the input. For example, depth of a node indexed 27 in heap is calculated as: $depth(27) = \left\lceil {\log _{3} (81 - 27 + 1)} \right\rceil - 1 = \left\lceil {{\text{2}}{\text{.6476}}} \right\rceil = 3$
4. colleague (*i*): Assuming that nodes at the same level of node *i* are the colleagues of this node. The index of any elected colleague of node *i* is returned by this step and the index can be calculated by generating any random integer in the range $\frac{dd^{depth(i)-1)}-1}{D-1} +1, \frac{dd^{depth(i)-1)}-1}{D-1}$.
5. Heapify_Up (*i*): searching upward in the heap then add node *i* at its correct place to save the heap property. Algorithm 1 show the pseudo code of this operation.
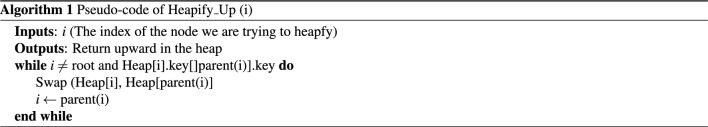
  Finally, the algorithm to build the heap is shown in Algorithm 2.
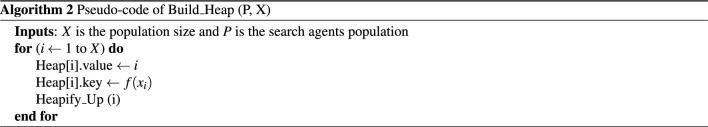
6. Repeated applications of position updating mechanism: search agents’ position is repeatedly updated according to previously explained equations trying to converge on the optimum global. The pseudo code of HBO is shown in Algorithm 3.
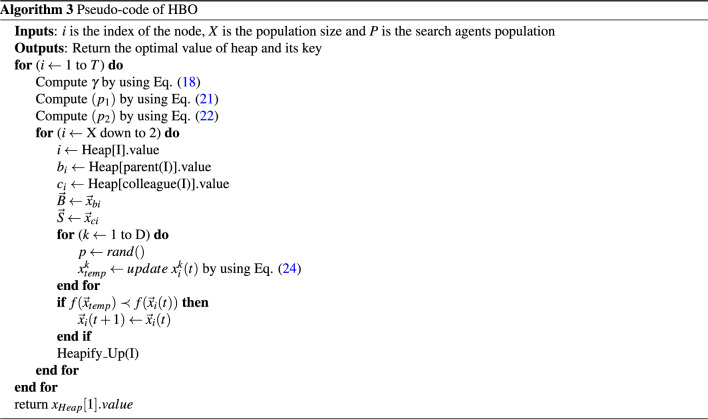

### Opposition-based learning (OBL)

The idea of opposition-based learning (OBL) is applicable strategy of search strategy to avoid stagnancy in candidate solutions. OBL is a novel concept inspired from the opposite relationship between entities^57^. The concept of opposition was presented in 2005 as the first time, which has attracted a many of research efforts in the last decennium. Many of Met-heuristic algorithms use the concept of OBL to develop their performance such as harmony search algorithm^58^, grasshopper optimization^59^, ant colony optimization^60^, artificial bee colony^61^ and etc. OBL improve the exploitation phase of a search mechanism. Mostly in meta-heuristic algorithms, convergence occurs quickly when the initial solutions are closer to the optimal location; moreover, late convergence is expected. So that, OBL method produce novel solutions by considering opposite search areas which may prove to be nearer to the best solution. OBL is regraded not only the candidate solutions obtained by a stochastic iteration scheme, but also their ’opposite solutions’ located in opposite parts of the search space. The OBL method has been hybridized with many bio-inspired optimization gives shorter expected distances to the best solution compared to randomly sampled solution pairs^62^ such as cuckoo optimization algorithm^63^, shuffled complex evolution algorithm^64^, particle swarm optimization^65^, harmony search^66^, chaotic differential evolution algorithm^67^, and shuffled frog algorithm^68^. In optimization problems, the strategy of simultaneously examining a candidate and its opposite solution has the purpose of accelerating the convergence rate towards a globally best solution. According to previous related works, in initialization phase utilize OBL only to improve the convergence and prevent stuck in local optima of HBO, then IHBO is utilized to solve problem of multi-thresholding for image segmentation by use two objective functions called Kapur and Otsu.

## The proposed IHBO algorithm

In this paper, the HBO algorithm is enhanced based on the OBL as local search strategy called IHBO to evade the drawbacks of the random population and improve the rate of convergence of the algorithm by developing the variety of its solutions. IHBO uses OBL strategy in the initialization phase to improve the search process as following:

28 $$\begin{aligned} Q_i = LB_j+UB_j-X_i, i\in {1,2,\ldots ,n} \end{aligned}$$

where $Q_i$ is a vector-maintaining solution resulting from the use of OBL, and $UB_j$ and $LB_j$ are the upper and lower bounds of the $j^{t} h$ component of a vector *X*. The phases of the proposed image thresholding model are described in depth below.

### Initialization phase

In this phase, the algorithm starts by reading the image, converting it to grayscale, computing the histogram of the selected benchmark images, and then computing the probability distribution by (1). The algorithm initializes the IHBO parameters, which are the population size (*N*), maximum iteration number (*T*), boundaries of the search space ($L_{I}$, $U_{I}$), and number of iterations per cycle (*t*). Thereafter, the OBL strategy is utilized to calculate the $Q_i$ vector-maintaining solution by (28).

### Updating phase

**Figure 1:**
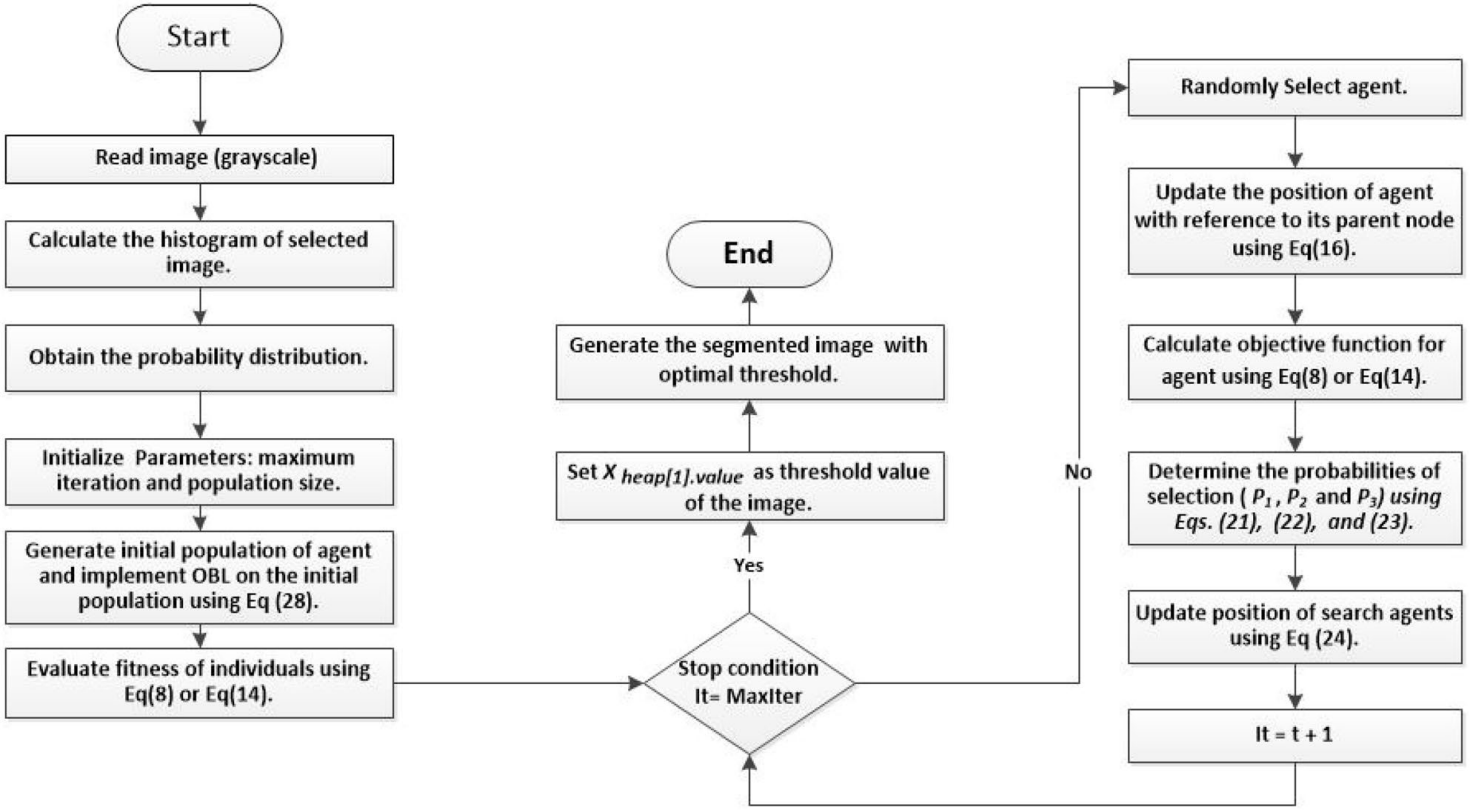
Flowchart of the proposed algorithm.

This phase provides the best threshold values by evaluating the fitness value of $X_i$ and $Q_i$ populations. To update the search agents’ positions (*X*), we use the fitness value of the optimal threshold of the Otsu $F_{Otsu}$ method (8) or Kapur $F_{kapur}$ method (14) as the objective function then comparing the fitness value of $X_i$ and $Q_i$ and saving the global best solution with the highest fitness. We define the position of each agent based on the fitness value. In addition, we determine three probabilities of selection $P_{1}$, $P_{2}$, and $P_{3}$ using (21), (22), and (23) sequentially, and then, based on the probabilities, we calculate the position of each agent within the heap using (24). The agent’s position (*X*) is updated using important $D-ary$ heap-based operations, such as Heapify_Up(*i*), which is used to search for the superior node in the heap, and we insert the node at its correct position to preserve the heap characteristics, as demonstrated in Algorithm 1. Then, each agent upgrades its location frequently according to the best fitness value, and seeks the global optimum, as depicted in Algorithm 3. Optimization scenarios of implementing the proposed IHBO algorithm illustrated in Figure 1.

### Segmentation phase

In this phase, we generate the segmented image with the optimal threshold value in an image after setting $x_{heap}[1].value$ as the threshold value of the image. The pseudo-code of the proposed IHBO algorithm is illustrated in in Algorithm 4.
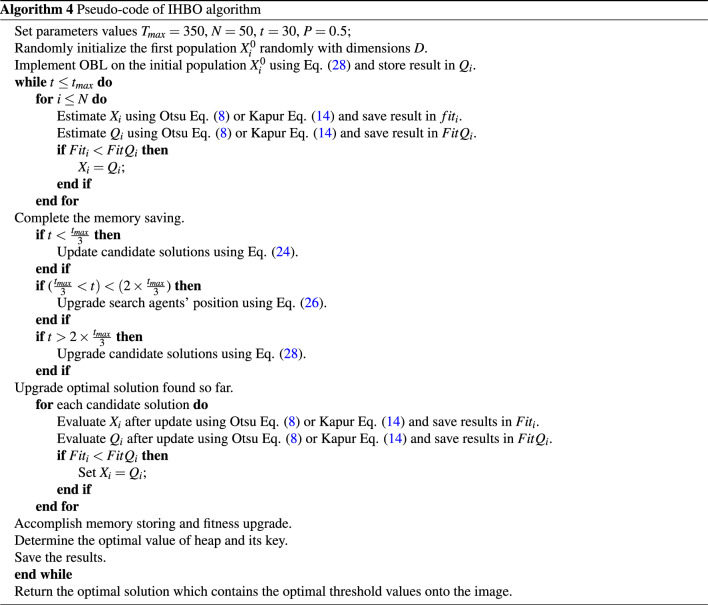

### Computational complexity of the IHBO

This section discusses the computational complexity of IHBO algorithm. The complexity of the population’s initialization can be represented as ${\mathcal {O}}(N \times D)$ time complexity, where *D* and *N* indicate the dimension of the problem and the size of the population, respectively. Additionally, the IHBO calculates the complexity with the fitness of each search agent as ${\mathcal {O}}\left( N \times D \times T_{\max } \right)$, where the maximum number of iterations is $T_{\max }$. Besides, the IHBO requires ${\mathcal {O}}(t)$ time complexity for executing *t* number of its main operations. Therefore, the time complexity of the proposed IHBO is ${\mathcal {O}}\left( N \times D \times t \times T_{\max }\right)$. But, the total amount of space occupied by the algorithm is called the space complexity. So, the space complexity of the proposed IHBO can be represented by ${\mathcal {O}}(N \times D)$.

## Performance evaluation of the proposed IHBO algorithm

### Parameter settings

This section provides the estimation of the proposed IHBO algorithm. As we all know, adjusting parameters will certainly affect the performance of an algorithm. However, according to the suggestion of Arcuri et al.^69^, when comparing algorithm performance, all algorithm parameters should be kept at their default values, which come from their original papers, to ensure they are in a relatively optimal state. Moreover, we reduce the risk of better parametrization bias as each algorithm is set to its default values. Therefore, in this work, all algorithm parameters are kept at their default values.

**Figure 2:**
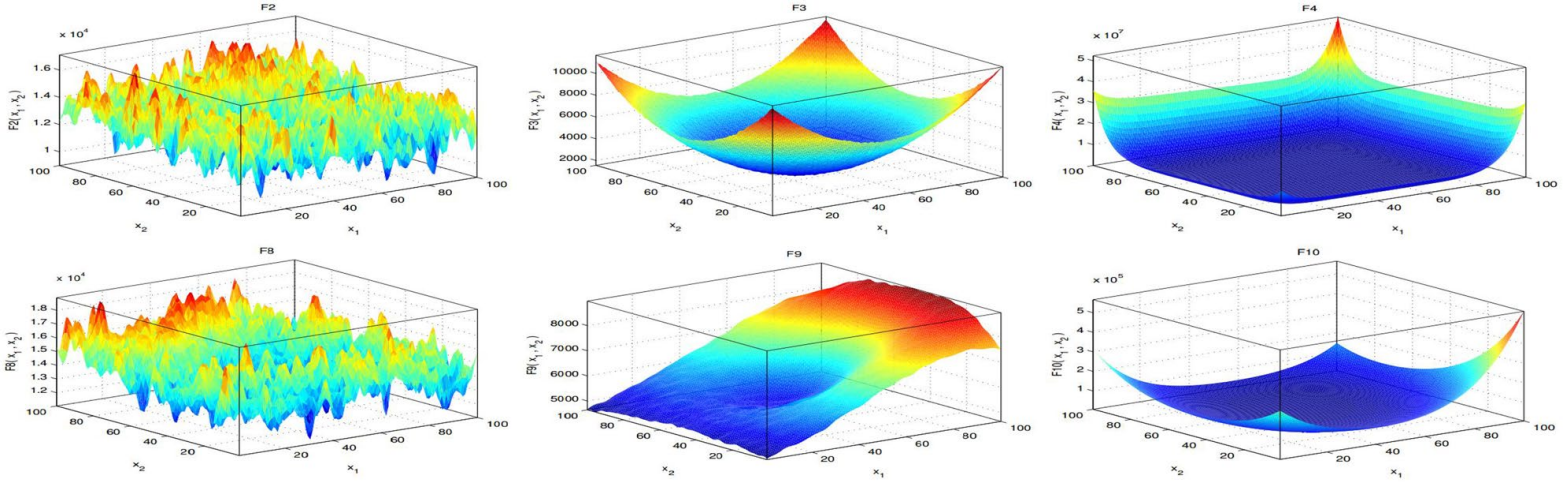
CEC’2020 benchmark functions in 2D view.

Thus, the performance of the proposed IHBO algorithm is evaluated over the IEEE Congress on Evolutionary Computation (CEC’2020)^70^ as test problems. The CEC’2020 benchmark functions is utilized to test the performance of IHBO algorithm. Initially, this benchmark functions contained 10 test functions referred to as $f_1$–$f_{10}$. Consequently, function 1 is unimodal functions, functions 2–4 are multimodal functions, functions 5–7 are hybrid functions, and functions 8–10 are composition functions. Table 1 illustrates the parameters setting and mathematical formulation of the CEC’2020 benchmark functions; ’Fi*’ refers to the best global value. Figure 2 illustrates a 2D visualization of the CEC’2020 benchmark functions to understand the differences and the nature of each problem.

**Table 1:** Parameter settings of CEC’2020 benchmark test.

No.	Function description	Fi*
Unimodal function		
F1	Shifted and Rotated Bent Cigar Function	100
Multimodal shifted and rotated functions		
F2	Shifted and Rotated Schwefel’s Function	1100
F3	Shifted and Rotated Lunacek bi-Rastrigin Function	700
F4	Expanded Rosenbrock’s plus Griewangk’s Function	1900
Hybrid functions		
F5	Hybrid Function 1 ($\hbox {N} = 3$)	1700
F6	Hybrid Function 2 ($\hbox {N} = 4$)	1600
F7	Hybrid Function 3 ($\hbox {N} = 5$)	2100
Composition functions		
F8	Composition Function 1 ($\hbox {N} = 3$)	2200
F9	Composition Function 2 ($\hbox {N} = 4$)	2400
F10	Composition Function 3 ($\hbox {N} = 5$)	2500

### Statistical results analysis of CEC’2020 benchmark

This section illustrates CEC’2020 benchmark test are utilized to estimate the performance of the proposed IHBO that contain qualitatively and quantitatively metrics. The standard deviation (STD) and mean of optimal solutions acquired by the proposed algorithm and all another algorithms utilized in the comparison is calculated. Furthermore, the qualitative metrics consists of average fitness history, convergence curve, and search history is used to evaluate the performance of the IHBO on the CEC’2020 test suite against seven well-known metaheuristic algorithms including the original HBO algorithm, SSA, MFO, GWO, SCA, HS, and EMO. Table 2 shows the STD and mean of the optimal value obtained from the proposed algorithm and the other competing algorithms for each CEC’2020 benchmark functions with 20 dimensional, and the optimal results of the STD and mean is minimum values in results. The results of the mean and STD of the proposed algorithm are proved superiority in solving seven CEC’2020 benchmark functions against to other competing algorithms.

**Table 2:** Mean and STD values of the optimal fitness obtained with competing algorithms on the CEC’2020 functions with $Dim=20$.

Functions	HS		SCA		MFO		GWO		SSA		HBO		IHBO	
	Mean	Std	Mean	Std	Mean	Std	Mean	Std	Mean	Std	Mean	Std	Mean	Std
F1	2.7133E+10	1.4183E+05	8.6737E+09	3.2590E+04	**2.4237E**+**07**	1.3700E+09	4.8253E+10	4.8874E+03	3.5695E+09	3.2884E+03	1.4994E+10	3.7401E+01	4.1417E+11	1.5482E+09
F2	3.1859E+09	3.1203E+10	5.1390E+09	4.2318E+09	4.4332E+09	6.6776E+09	4.9220E+10	6.6198E+07	4.2912E+09	4.4798E+08	3.3996E+09	2.1044E+08	**2.6221E**+**09**	2.7699E+07
F3	9.6206E+08	6.8217E+06	9.6568E+09	3.7090E+06	7.7019E+07	2.7089E+06	8.9464E+08	2.7347E+08	9.0421E+07	6.3036E+08	7.9531E+07	3.8975E+10	**1.1088E**+**07**	7.0401E+07
F4	2.0157E+08	1.3668E+08	3.4429E+08	5.3612E+05	**1.9991E**+**07**	9.7905E+08	6.6635E+09	2.4878E+11	2.5966E+08	1.5150E+05	2.0029E+07	2.3428E+06	6.7985E+07	5.9369E+10
F5	6.2557E+08	6.4993E+08	1.1866E+10	7.4304E+08	1.4980E+09	6.7269E+05	8.5775E+11	3.9047E+06	1.5708E+10	1.7314E+06	5.9495E+09	6.7486E+07	**1.4908E**+**08**	7.8877E+07
F6	3.3488E+04	7.5211E+00	2.7999E+05	2.6069E+04	9.4696E+04	3.7214E+03	2.2082E+05	0.0000E+00	1.7973E+04	6.2490E+02	4.0739E+03	0.0000E+00	**1.0872E**+**03**	3.7049E+01
F7	3.3488E+07	7.5211E+03	2.7999E+07	2.6069E+07	9.4696E+06	3.7214E+06	2.2082E+08	0.0000E+00	1.7973E+07	6.2490E+05	4.0739E+05	0.0000E+00	**1.0872E**+**05**	3.7049E+04
F8	**2.3744E**+**08**	5.3560E+05	3.2600E+08	1.4781E+08	2.6752E+08	7.2306E+08	5.1174E+09	6.4210E+06	5.1713E+08	2.7202E+10	2.3802E+08	6.4911E+11	6.6854E+08	0.0000E+00
F9	3.3557E+10	4.2292E+10	3.0644E+10	6.3500E+08	2.9123E+09	5.6238E+08	3.6391E+10	1.6099E+12	3.0745E+09	6.5302E+10	2.9969E+09	3.5113E+08	**1.0346E**+**09**	3.6709E+09
F10	3.1568E+08	6.0725E+08	3.3829E+08	3.1844E+08	3.0797E+09	3.8147E+05	3.4776E+10	2.1653E+07	3.3225E+08	6.2899E+07	3.0599E+08	1.6946E+10	**2.2474E**+**08**	5.9369E+09

Significant values are in bold.

### Boxplot analysis

**Figure 3:**
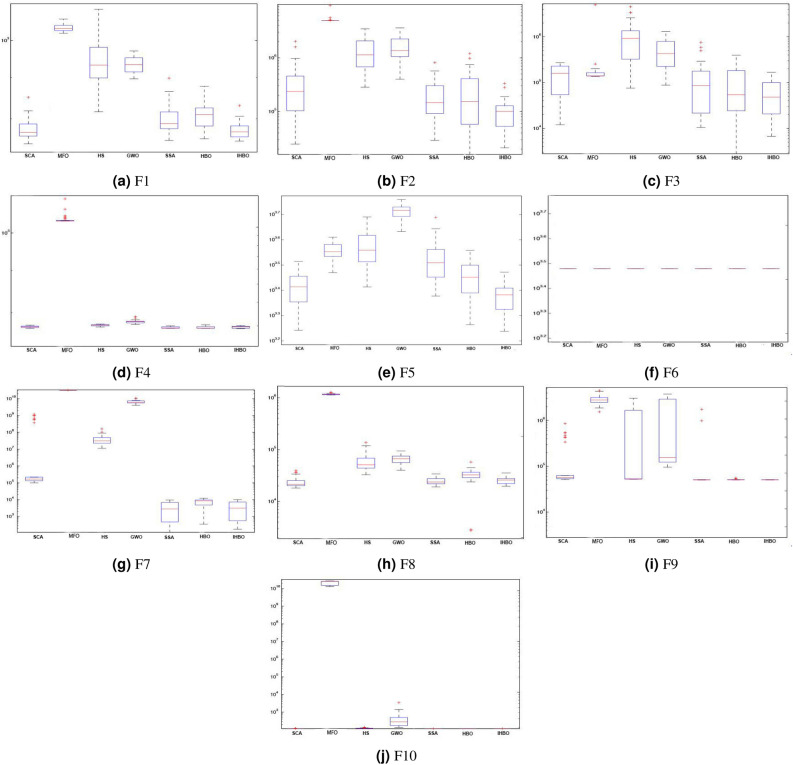
Results of Boxplots obtained all algorithms over CEC’2020 benchmark functions with $Dim=20$.

Boxplot analysis is a graphical technique used to display data distribution characteristics. The boxplot technique is designed to report data that follow a normal distribution and have homogeneous variances, the results of boxplot for all algorithms and them functions are illustrated in Fig. 3. Boxplot is most important plots to describe data distributions into quartiles. This quartiles are the median of the first half of the data is *first* quartile, the *second* quartile is the median, the *third* quartile is median of the second half of the data, and the largest observation. The region among the first and third quartile is called the interquartile range and used to give an indication of spread in the data. The ends of the rectangles determine the lower and upper quartiles and a narrow boxplot refers to highest agreement among data. Figure 3 shows the boxplots of the proposed IHBO algorithm and illustrates the results of ten functions boxplot for Dim = 20. In reality, the results of proposed algorithm are proved superiority than all other competing algorithms on most of the test functions, but the performance of proposed algorithm is limited on F2, and F7.

### Convergence curves analysis

**Figure 4:**
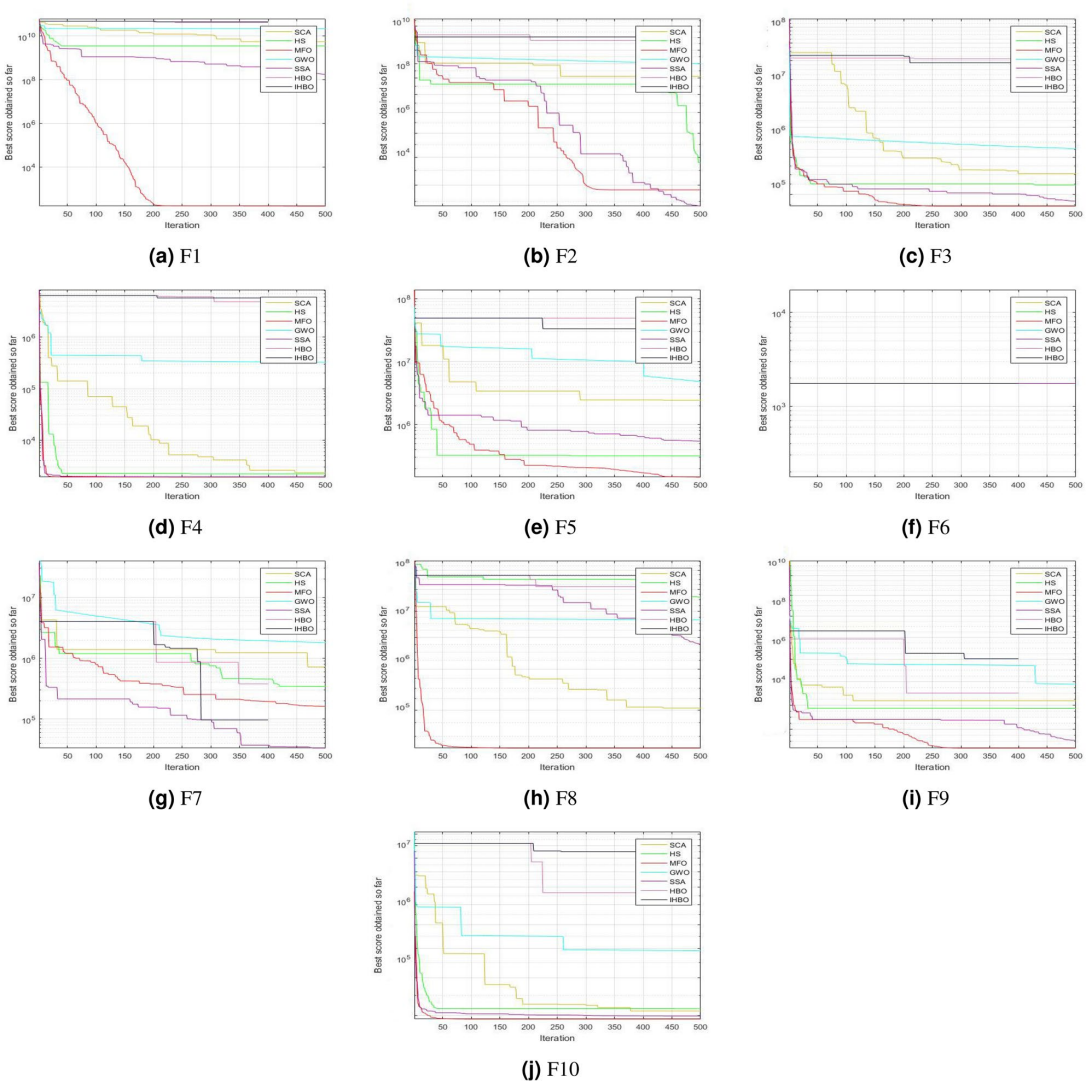
Results of convergence curves for the proposed algorithm with other competing algorithms over CEC’2020 benchmark functions with $Dim=20$.

This subsection explains the convergence plots of the proposed algorithm with other competitor algorithms. Figure 4 illustrates the convergence plots of IHBO, HBO, SSA, GWO, MFO, HS, and SCA for the CEC’2020 benchmark functions. Furthermore, the proposed algorithm achieved optimal solutions and reached a stable point for most functions. Thus, IHBO can solve problems that require fast computation, such as online optimization problems. The proposed algorithm exhibited stable behavior, and its solutions converged easily in most of the problems it was tested on. Due to space limitations.

### Qualitative metrics analysis

**Figure 5:**
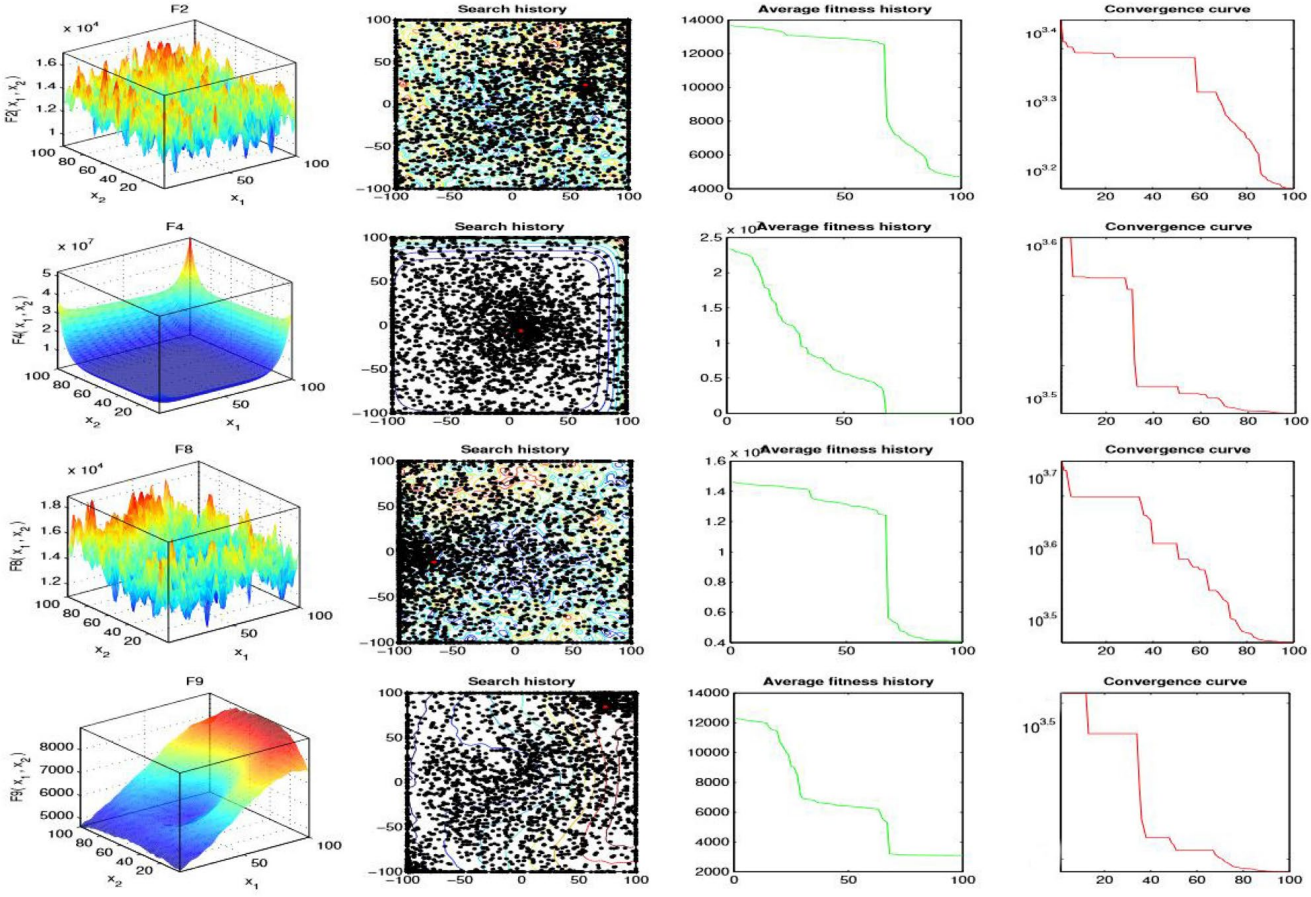
Qualitative metrics on F2, F4, F8, F9, and F10 in 2D view of the functions.

Even though the earlier outcome analyses assure the high performance of the proposed IHBO algorithm, the performance of more experiments and analyses would help us to draw more clear conclusions about the algorithm performance in real problem solving. Figure 5 illustrates the qualitative analysis of the proposed IHBO algorithm. The first column illustrates a set of the CEC’2020 benchmark functions as plots in two-dimensional space. The second column illustrates the search history of search agents, from the first to the last iteration and display their exploitation behavior to realize the desired outcomes. The third column shows the average fitness history over 350 iterations, explaining the general behavior of the agents and the role that they play in the search of the best solution. According to average fitness history, all the history curves are decreasing, which means that the population improves at each iteration. The fourth column shows convergence curve and optimization history revealed the progress of fitness over a number of iterations. Optimization history is decreasing indicates that the solutions are optimized during iterations until reach the best solution.

## Environmental and experimental requirements

This section presents the test images used for the experiments, then describes the empirical setup, and analyzes the results.

### Benchmark images

To evaluate the proposed method, ten images of common benchmark were used. The selected benchmark images due to the various levels of complexity and included the following images: Baboon, Lena, Butterfly, Pirate, Cameraman, Peppers, Bridge, Living Room, Barbara, and Jetplane^71,72^. Most images had the same dimensions (512 $\times$ 512 pixels); however, two images (Cameraman and Lena) were 256 $\times$ 256 pixels. Table 3 displays the set of test images used.

**Table 3:**
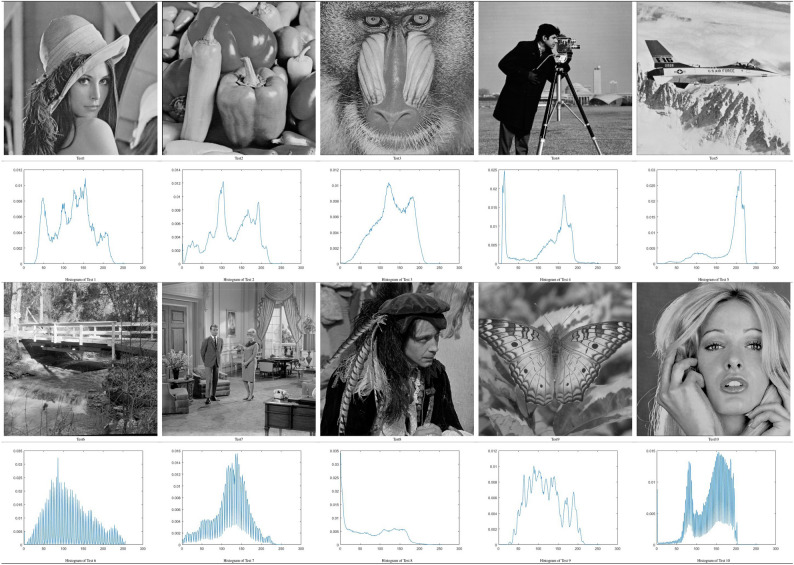
Set of test images.

### Environmental setup

In this study, the proposed IHBO is compared with seven well-known metaheuristic algorithms including the original HBO, SSA, MFO, GWO, SCA, HS, and EMO. All competitor algorithms were applied and executed in Matlab 2015, and implemented on PC with 6G RAM running in a Windows 8.1 64-bit environment with an Intel Core I5 processor. The counterparts were executed 30 times per test image, number of iterations was set to 350, and population size is 50. The parameters settings of each algorithm were determined according to standard criteria and information found in previous literature (default values). The number of thresholds used was $th_2,th_3,th_4,$ and $th_5$ according to related literature^73^. The parameters settings of the IHBO and their values are presented in Table 4.

**Table 4:** The parameters of IHBO and it’s value.

Parameter	Value
Maximum number of iterations	30
Number of local iterations	350
Size of population	50
Dimension of problem (*Dim*)	20
Mutation ratio	0.5
Values [*C*, $p_1$, $p_2$] from corresponding equations	

### Evaluation metrics

Two metrics were utilized to estimate the performance of the IHBO algorithm. The first metric was used to evaluate the quality of the image, while the second metric was used to compare the edges of the segmented image. These metrics are important for evaluating the performance of the IHBO approach based on the Otsu and Kapur methods as objective functions. Statistical tests, such as the standard deviation (STD), Wilcoxon rank test, and average, were used to analyze the fitness of the proposed algorithm. We used the SSIM^48^, FSIM^74^, and PSNR^75^ to measure the quality and stability of the image.

#### Structural similarity index (SSIM)

The SSIM^48^ index is a metric that is used to analyze the internal structures in a segmented image. A higher SSIM value indicates better segmentation of the original image due to the fact that structures in the two images match. The equation below describes the SSIM:

29 $$\begin{aligned} SSIM(I,Seg)= \dfrac{(2\mu _1 \mu _{Seg} + c_1)( 2\sigma _{1,Seg} +c_2 )}{(\mu _I^2 + \mu _{Seg}^2 + c1)(\sigma _I^1 + \sigma _{Seg}^2 +c2)} \end{aligned}$$

The mean of the intensities of the original image *I* and segmented image Seg are $\mu _I$ and $\mu _{Seg}$, respectively, and $\sigma _I$ and $\sigma _{Seg}$ are the standard deviations of the original image *I* and segmented image *Seg*, respectively. $\sigma _{I,Seg}$ is the covariance of the original image *I* and segmented image *Seg*, and $c_1$ and $c_2$ are two constants.

#### Feature similarity index (FSIM)

The FSIM^74^ index is a metric that is used to compute the similarity between the segmented image and original image based on their internal features. A higher FSIM value indicates better segmentation by the thresholding method. The FSIM can be described in the following steps:

30 $$\begin{aligned} FSIM= \frac{ \sum _{v\epsilon \Omega }S_L(v)PC_{m}(v)}{\sum _{v\epsilon \Omega }PC_{m}(v)} \end{aligned}$$

The entire domain of the image is $\omega$:

31 $$\begin{aligned} S_L(v)= & {} S_{PC}(v)S_G(v) \end{aligned}$$

32 $$\begin{aligned} S_PC(v)= & {} \frac{2PC_1(v)PC_2(v)+T_1}{PC_1^2(v)+PC_2^2(v)+T_1} \end{aligned}$$

33 $$\begin{aligned} S_G(v)= & {} \frac{2G_1(v)G_2(v)+T_1}{G_1^2(v)+G_2^2(v)+T_1}, \end{aligned}$$

and *G* is the image’s gradient magnitude and can be computed as follows:

34 $$\begin{aligned} G= & {} \sqrt{G_x^2+G_y^2} \end{aligned}$$

35 $$\begin{aligned} PC(v)= & {} \frac{E(v)}{\left( \epsilon + \sum _{n}A_n(v) \right) } \end{aligned}$$

The vector’s magnitude in *v* on n is *E*(*v*), and the local amplitude of scale *n* is $A_n(v)$. The small positive number is $\epsilon$ and $PC_m(v) = max(PC_1(v),PC2(v))$.

#### Peak signal-to-noise ratio (PSNR)

The PSNR^75^ is another metric used to evaluate the quality of segmentation by determining the difference between the quality of the original image and that of the segmented image. The PSNR is used to compare the original and segmented image using the root mean square error (RMSE) of each pixel, as expressed in (37). The PSNR can be defined as follows:

36 $$\begin{aligned} PSNR=20log_{10} \dfrac{255}{RMSE}, \end{aligned}$$

where

37 $$\begin{aligned} RMSE = \sqrt{\dfrac{\sum _{i=1}^M \sum _{j=1}^N ((I(i,j)-Seg(i,j))^2)}{M x N}}. \end{aligned}$$

In (37), *I* and *Seg* are the segmented and original images of size $M \times N$, respectively. A higher PSNR value indicates that there is higher similarity between the segmented and original images, which reflects a more effective segmentation process.

## Experimental results and discussion

The experimental results are discussed in this section to evaluate the efficiency of the proposed algorithm.

### Otsu results analysis

This subsection analyzes the outcomes of the IHBO based on the between-class variance as the fitness function, as proposed by Otsu. Table 7 illustrates the best threshold values obtained by applying the IHBO with the Otsu entropy as the objective function (8). Tables 5 and 6 present a graphical analysis of the thresholds, illustrating the resulting images of the IHBO with a different number of thresholds. Table 8 shows the computational time values of comparison algorithms obtained by Otsu’s method. The IHBO proved its superiority in computational time compared to other competitive algorithms with 23 cases in 40 experiments and came in the first place. GWO came in second place with 10 experiments, while HBO come in third place with nine experiments, followed by EMO with two experiments. Finally, the MFO came in fifth place with only one experiment, and the remaining algorithms could not obtain the best computational time in any of the experiments. Table 9 illustrates the Otsu STD and average of the fitness results for the benchmark images. The IHBO demonstrated superiority in MTH by obtaining an optimal fitness values for 23 cases in 40 experiments. The HBO algorithm obtained the best fitness value in eight experiments, while the SCA obtained the optimal fitness value in five experiments and SSA come in fourth place with four experiments followed by MFO with three experiments. Finally, HS obtained the optimal fitness value in only one experiments and the remaining algorithms could not obtain the optimal fitness value in any of the experiments. Table 9 illustrates the STD values calculated for the 40 independent outcomes for each tested image with various thresholds. A lower STD value indicates that the algorithm is more stable.

Table 10 presents the STD and mean PSNR for the benchmark images using the eight MAs. The IHBO was in first place in terms of the mean values of PSNR in 22 experiments. The SSA was in second place in seven experiments, while HBO was in third place, as it was superior in only six experiments. In fourth place was SCA with the best PSNR in only five experiments followed by MFO and HS with three experiments. Finally, the worst results were obtained by EMO which did not obtain the optimal values of the PSNR in any of the experiments. With respect to the STD, the IHBO was not the best alternative for lower dimensions (2 or 3 *th*). This is because the STD value was higher, which represents higher instability of the algorithm. However, MFO was a more unstable algorithm in terms of the PSNR. For the remaining approaches, the STD values followed the same tendency: lower for small dimensions and higher for four thresholds. However, the SSA was the least unstable algorithm, while the SCA was in second place. HBO was in the third place, HS was in fourth place, and the IHBO was in fifth place. Furthermore, GWO was in sixth place, and EMO was in seventh place.

Table 11 illustrates the STD and mean of the FSIM obtained from 40 experiments. The results of the FSIM indicate that the IHBO obtained the highest FSIM in 22 experiments and was in first place, while the HBO was in second place in ten experiments. However, the SCA was in third place in eight experiments. SSA was in fourth place in two experiments, followed by HS and EMO, which appeared in fifth place in only one experiment. Finally, GWO and MFO came in last place in the experiments. The SCA was thus the best approach in terms of the STD because its values were lower in most experiments. The SSA came in second place, followed by EMO in third place. Then, the IHBO appeared in fourth place. GWO was in fifth place, followed by HBO. Finally, the least stable approaches were MFO and HS due to their high STD values in most cases.

Table 12 presents the results of the STD and mean SSIM obtained in 40 experiments. The IHBO came in first place in terms of mean PSNR with the best SSIM in 22 experiments, while the SCA, HBO, and SSA came in second place in six experiments with higher SSIM values. EMO came in the third place in two experiments, followed by HS and MFO, which came in fourth place with only one experiment. Finally, GWO came in last place in the experiments. Because it provided the largest number of minimum values of the STD of all algorithms, SCA was the best method. In second place was the HBO, followed by EMO, which was in third place. The IHBO was in fourth place, while GWO was in fifth. Finally, MFO, HS, and SSA had no minimum STD values in the experiments.

Table 7 illustrates the thresholds that were applied on the selected benchmark images. In Tables 5 and 6, the histograms are illustrated with the respective threshold values and the segmented images of the selected images using 2, 3, 4, and 5 thresholds. These results indicate that for some images, there was improvement in the quality of their contrast as the number of thresholds increased, particularly for the images Butterfly, Living Room, Jetplane, Lena, Pirate, Cameraman, Lake, and Bridge, presenting a higher amount of information in the image with the largest number of thresholds when compared with an image with only two thresholds. The most difficult histograms to segment were for Test 6, 9, and 10, relating to Bridge, Butterfly, and Barbara, respectively. The complexity was due to different numbers of pixels in the images, which could produce several classes or even make it impossible to select the optimal thresholds.

Table 13 presents the p-values resulting from the Wilcoxon test for fitness using the Otsu fitness function. This table presents the difference between the proposed algorithm and the compared algorithms (HBO, SSA, MFO, GWO, SCA, HS, and EMO).

A difference between the SCA and MFO in comparison to the IHBO can be observed, which indicates that the proposed algorithm has a significant development. However, for the number of thresholds (nTh) = 5, the differences between the IHBO and most of the competing algorithms are clear by performing the comparison over 30 runs in each experiment. In the results, NaN indicates that the dataset to be compared is the same. This signifies that the algorithms obtained the same solution; thus, their results from the Wilcoxon test reveal that they are similar and that there are no differences between the methods.

**Table 5:**
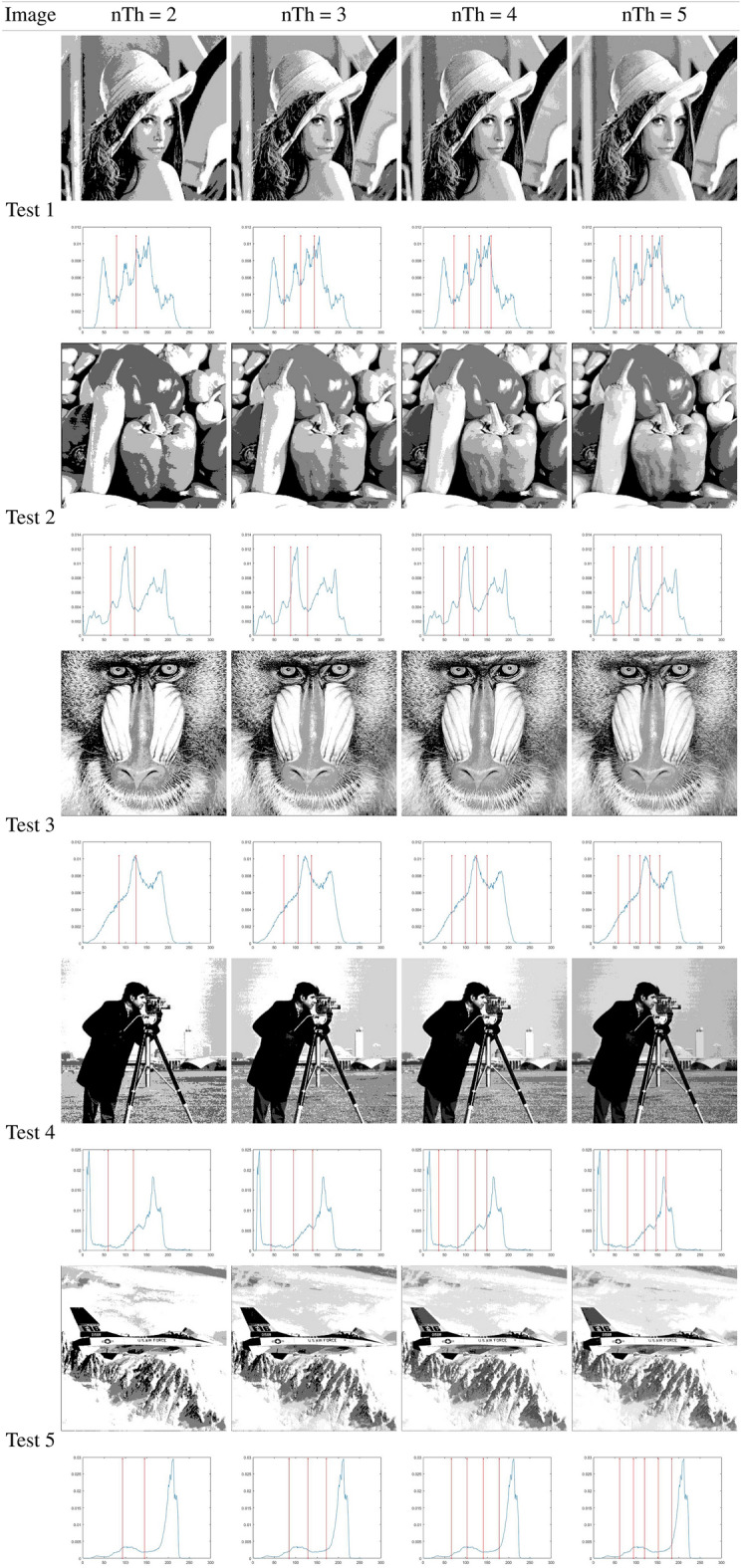
Implementation results of IHBO-Otsu over the set of benchmark images.

**Table 6:**
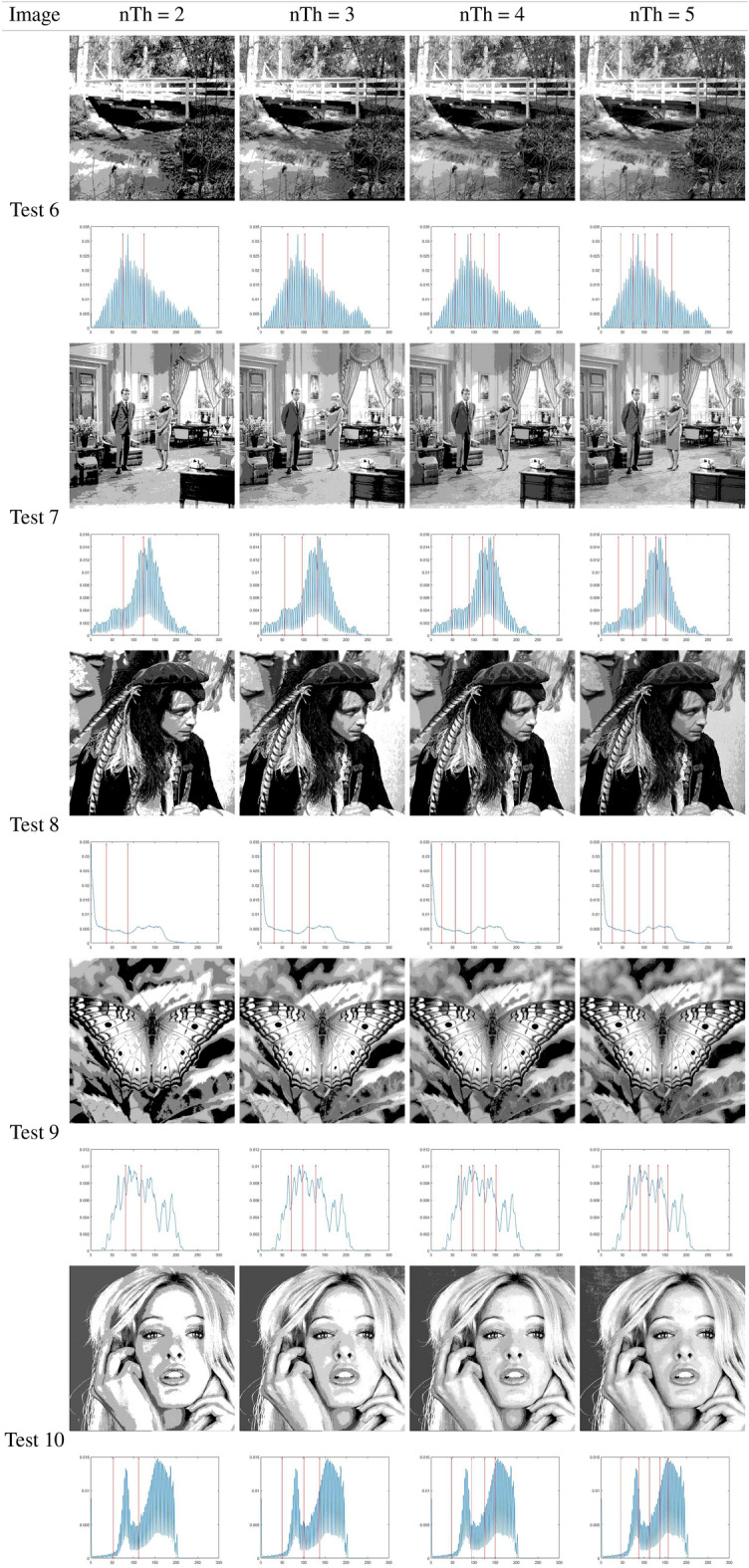
Implementation results of IHBO-Otsu over the set of benchmark images.

**Table 7:** The best thresholds values obtained by Otsu’s method.

Test Image	nTh	IHBO	HBO	SSA	MFO	GWO	SCA	HS	EMO
Test 1	2	81 124	80 125	79 125	79 125	79 125	79 125	79 125	79 125
	3	72 114 144	71 112 143	75 115 139	71 111 142	74 119 144	70 116 144	70 116 140	71 106 140
	4	72 108 135 159	71 108 131 155	70 102 124 149	71 105 130 153	73 105 139 159	70 106 134 157	73 102 133 148	70 100 121 144
	5	62 88 114 139 161	62 88 125 139 167	36 82 102 139 163	62 87 125 140 169	52 77 117 141 163	58 51 116 138 162	63 81 118 136 160	62 88 122 136 165
Test 2	2	65 122	65 122	65 122	63 122	65 122	65 121	65 122	65 122
	3	50 88 128	65 122 129	55 89 126	58 88 124	59 92 129	53 85 124	59 91 127	50 86 123
	4	48 85 118 150	48 85 118 150	50 88 118 151	48 82 115 149	46 81 117 144	42 87 110 141	45 88 115 150	39 80 112 140
	5	48 81 107 133 160	48 85 102 145 159	48 85 108 140 159	48 88 101 143 160	48 83 106 138 157	44 83 101 138 155	48 85 103 145 150	48 79 102 141 160
Test 3	2	98 152	97 149	97 149	97 150	97 149	97 149	97 149	97 149
	3	72 116 137	80 120 131	85 121 131	70 118 136	77 110 134	82 110 135	73 119 132	85 116 130
	4	66 98 124 149	58 102 122 160	52 106 127 158	58 92 120 145	51 79 124 151	63 84 125 153	51 104 127 155	51 108 125 155
	5	58 85 110 133 157	67 91 120 136 154	54 83 109 134 151	66 90 122 134 152	54 86 105 135 151	59 86 110 134 160	69 91 125 133 153	67 88 124 130 151
Test 4	2	59 119	59 119	59 119	59 119	59 119	59 119	59 119	59 119
	3	42 95 140	59 112 139	38 92 138	60 110 140	52 115 140	48 110 137	38 93 139	34 90 136
	4	36 83 123 150	31 86 140 152	38 92 138 159	30 85 142 151	38 91 137 149	37 83 139 152	37 92 137 149	29 75 130 149
	5	36 81 120 147 170	36 82 122 149 172	35 81 118 147 162	35 82 121 148 171	35 81 121 144 160	35 82 120 147 173	36 82 120 148 171	35 82 121 145 170
Test 5	2	93 145	93 145	93 145	92 145	93 145	93 145	93 145	92 145
	3	84 129 172	83 125 171	79 121 168	83 124 170	82 126 164	83 125 171	79 120 168	83 123 171
	4	67 105 142 180	63 109 152 173	84 129 152 182	67 103 141 178	68 107 140 178	68 107 143 175	67 103 140 176	84 128 154 180
	5	66 101 135 166 191	68 106 142 169 190	68 106 132 159 184	68 104 140 169 193	22 91 115 147 187	22 91 115 147 187	68 106 140 170 191	68 108 131 160 182
Test 6	2	75 126	75 124	75 124	75 124	75 124	75 124	75 124	75 124
	3	63 103 145	65 104 140	65 102 139	65 102 136	66 107 136	62 107 141	65 100 134	65 102 137
	4	55 88 120 156	60 103 125 190	50 88 128 171	60 106 122 175	56 77 124 177	59 69 122 172	50 89 125 170	52 88 129 170
	5	46 76 103 134 164	46 81 104 139 161	48 80 103 132 165	44 83 106 139 160	39 69 101 138 168	41 87 109 138 161	46 81 104 139 161	46 81 111 134 160
Test 7	2	76 123	75 123	75 123	75 123	71 120	75 123	75 123	75 123
	3	55 97 132	56 103 133	56 103 133	49 105 132	45 108 128	56 103 133	56 105 131	44 108 131
	4	49 88 121 147	46 87 122 148	46 86 124 148	46 87 122 148	47 88 123 147	46 84 123 148	46 85 123 149	46 86 120 145
	5	41 76 105 129 153	43 88 102 126 158	41 88 108 129 159	43 88 100 120 157	41 61 111 121 161	38 81 110 123 156	43 88 104 125 155	43 89 110 121 159
Test 8	2	51 116	51 116	51 116	51 115	51 116	51 116	51 115	51 115
	3	31 73 112	36 76 115	36 74 116	36 72 110	35 74 114	36 76 114	38 74 114	40 62 108
	4	25 55 89 126	23 59 90 106 127	30 61 90 126	22 59 94 125 123	31 56 88 127	30 52 85 126	30 61 92 125	30 65 8790 125
	5	22 52 83 110 136	22 53 86 112 132	22 53 88 111 132	20 52 80 109 134	22 55 78 111 130	21 53 99 125 140	22 53 86 114 131	22 51 82 115 130
Test 9	2	83 119	81 118	81 118	81 118	81 118	81 118	81 118	81 118
	3	72 99 127	70 96 129	71 108 129	70 90 125	71 95 127	72 92 120	71 105 128	70 94 128
	4	71 98 124 152	72 99 123 152	63 103 125 153	72 95 120 151	74 94 124 158	69 96 123 152	72 99 127 154	63 113 126 154
	5	69 90 111 132 155	71 89 114 132 159	68 71 113 139 159	71 85 114 130 155	61 80 92 115 158	63 81 111 130 159	71 89 114 131 165	70 89 111 130 154
Test 10	2	55 114	52 112	52 112	52 112	52 112	52 112	52 112	52 112
	3	52 101 138	49 102 138	53 102 138	50 101 136	54 100 129	47 102 132	51 102 137	52 109 135
	4	50 95 125 150	50 101 127 147	49 100 126 146	50 101 127 147	42 97 124 143	48 92 120 142	49 98 121 140	49 98 124 148
	5	46 91 117 139 160	48 90 115 140 159	47 88 103 139 162	46 90 117 137 158	41 86 103 141 159	40 79 119 143 161	48 92 114 142 157	50 91 112 141 153

**Table 8:** The computational time values of comparison algorithms obtained by Otsu’s method.

Test Image	nTh	IHBO		HBO		SSA		MFO		GWO		SCA		HS		EMO	
		Mean	STD	Mean	STD	Mean	STD	Mean	STD	Mean	STD	Mean	STD	Mean	STD	Mean	STD
Test 1	2	0.5652	3.1416E-01	0.6926	7.0576E-01	0.7961	4.9555E-01	0.5781	3.1218E-01	**0.5043**	2.8886E-01	0.6464	6.3756E-01	0.6359	2.6455E-01	0.6463	3.1111E-01
	3	**0.4634**	3.8720E-02	0.5323	1.0758E-01	0.6453	2.0229E-01	0.5630	1.2397E-01	0.5793	1.0142E-01	0.6819	3.6190E-01	0.6193	1.1858E-01	0.6819	1.3945E-01
	4	**0.4337**	4.5210E-02	0.9825	2.7709E-01	0.6215	1.6423E-01	0.5252	5.0820E-02	0.4713	2.6840E-02	0.7821	9.3830E-02	0.5777	3.8720E-02	0.7820	4.5535E-02
	5	**0.4352**	4.7080E-02	0.9205	1.0670E-01	0.6137	1.8678E-01	0.5482	1.3783E-01	0.4926	3.7180E-02	0.7857	7.9750E-02	0.6030	5.0710E-02	0.7856	5.9635E-02
Test 2	2	0.6488	3.3770E-02	0.5741	3.0052E-01	0.7236	2.3441E-01	0.7423	9.0090E-02	**0.5295**	1.2100E-02	0.9135	2.4068E-01	0.8165	1.1792E-01	0.9134	1.3867E-01
	3	0.9051	1.3200E+01	**0.5305**	1.2859E-01	0.9649	3.6256E-01	0.8414	1.9767E-01	**0.5305**	1.0120E-02	0.8508	2.3100E-01	0.9256	4.0733E-01	0.8507	4.7902E-01
	4	0.7256	1.4960E-02	0.8701	1.8095E-01	0.9772	7.5636E-01	0.8050	1.2320E-01	**0.6547**	1.1990E-02	0.8758	1.8535E-01	0.8855	1.5367E-01	0.8757	1.7303E-01
	5	**0.6570**	1.1110E-02	0.8759	9.5480E-02	0.9779	7.9266E-01	0.8343	9.4820E-02	0.6749	1.4628E-02	0.8893	1.5235E-01	0.9177	4.2570E-01	0.8892	4.7934E-01
Test 3	2	**0.5602**	1.0670E-02	0.8046	1.4916E-01	0.8584	5.4142E-01	0.7366	6.5120E-02	0.6113	2.4100E-02	0.8544	2.7258E-01	0.8103	2.7026E-01	0.843	3.0431E-01
	3	0.5259	9.1300E-03	**0.4801**	9.8010E-02	0.8131	4.8246E-01	0.8016	1.0615E-01	0.6253	1.7146E-02	0.8406	2.2460E-01	0.8818	1.4435E-01	0.8405	1.6253E-01
	4	**0.4662**	9.1300E-03	0.4900	3.0569E-01	0.8405	4.3813E-01	0.9729	2.8446E-01	0.6547	4.8823E-01	0.9092	1.5528E-01	1.0701	9.9876E-02	0.9091	1.1246E-01
	5	**0.4178**	2.5892E-02	0.4718	2.2671E-01	0.9731	9.1850E-01	0.7714	1.0098E-01	0.6940	3.6330E-02	0.8769	1.4981E-01	0.8485	1.5409E-01	0.8768	1.7351E-01
Test 4	2	0.4121	6.9300E-02	**0.4039**	1.9448E-01	0.8385	4.8444E-01	0.6912	4.0810E-02	0.4285	5.7672E-02	0.6760	2.7763E-01	0.7603	2.5801E-02	**0.4039**	2.9052E-02
	3	0.4333	3.4948E-02	0.4682	1.2121E-01	0.8624	4.7234E-01	0.7288	6.7100E-02	0.4451	3.1893E-02	0.6149	1.4125E-01	0.8017	3.5551E-02	**0.4149**	4.0030E-02
	4	0.4585	3.5388E-02	**0.4436**	1.1528E-01	0.9959	6.2370E-01	0.7387	3.7400E-02	0.4647	1.1870E-02	0.6912	2.5730E-01	0.8126	2.9131E-02	0.4912	3.2802E-02
	5	**0.4473**	8.8689E-02	0.4604	2.3199E-01	1.2890	1.3500E+00	0.7356	5.1590E-02	0.4898	2.3980E-02	0.6012	1.9012E-01	0.8092	1.0297E-01	0.5012	1.1594E-01
Test 5	2	0.6028	3.5168E-02	**0.5735**	7.2930E-02	0.8597	4.9159E-01	0.6680	4.1030E-02	0.7032	1.3549E-02	0.6204	2.9749E-01	0.7348	7.0983E-02	0.6142	7.9927E-02
	3	**0.5067**	2.1760E-02	0.5765	2.0988E-01	0.8965	7.4514E-01	0.7108	5.0118E-02	0.7249	9.7120E-03	0.8450	4.3708E-01	0.7818	6.0877E-02	0.8365	6.8548E-02
	4	**0.5189**	5.7150E-03	0.6712	6.0280E-02	1.0702	8.2478E-01	0.7183	6.6784E-02	0.7514	1.4028E-02	0.9586	3.8987E-01	0.7902	1.8810E-01	0.9490	2.1180E-01
	5	**0.5004**	5.1763E-02	0.7004	7.6230E-02	1.1645	7.8078E-01	0.7389	6.5945E-02	0.8056	5.5394E-02	0.9699	1.5457E-01	0.8128	2.6527E-01	0.9601	2.9869E-01
Test 6	2	**0.3997**	2.4837E-02	0.7587	3.5472E-02	0.9604	1.8348E-01	1.0563	6.9926E-01	**0.3997**	1.3189E-02	0.8271	3.0866E-01	1.1620	5.3753E-02	0.8187	5.9451E-02
	3	**0.4492**	5.4730E-02	0.6675	7.1221E-02	0.8845	6.4251E-01	0.9728	3.1318E-01	0.4573	1.9784E-02	0.8071	2.5849E-01	1.0701	1.7153E-01	0.7989	1.8971E-01
	4	0.4549	2.0771E-02	0.7090	8.5269E-02	0.6991	3.0492E-01	0.7689	1.9939E-01	**0.4539**	1.3549E-02	0.8437	3.1925E-01	0.8458	6.2836E-02	0.8352	6.9497E-02
	5	**0.4692**	2.4177E-02	0.7047	1.0302E-01	0.7083	1.2606E-01	0.7791	7.2899E-02	0.4900	4.9399E-02	0.8053	9.9400E-02	0.8570	1.2805E-01	0.7972	1.4163E-01
Test 7	2	0.4509	3.3190E-02	0.6757	1.2360E-01	0.4790	3.6707E-02	0.5268	1.3225E-01	**0.3931**	1.5107E-02	0.7903	1.9452E-01	0.5742	4.5502E-02	0.7824	5.0325E-02
	3	**0.4036**	2.5387E-02	0.6619	1.0912E-01	0.5345	3.3959E-02	0.5879	1.6738E-01	0.4636	1.1414E-01	0.8713	2.9999E-01	0.6408	1.1446E-01	0.8625	1.2660E-01
	4	**0.4834**	1.3562E-01	0.6949	1.8872E-01	0.7648	1.6870E-01	0.8412	9.4601E-02	0.6201	1.6906E-02	0.8249	1.0618E-01	0.9169	3.9701E-02	0.8166	4.3909E-02
	5	**0.4511**	1.6661E-01	0.6434	4.0184E-02	1.0059	1.0034E-01	1.1064	6.2228E-02	0.6565	4.3670E-01	0.8755	2.2056E-01	1.2060	1.3547E-01	0.8667	1.4983E-01
Test 8	2	**0.4889**	1.2188E-01	0.6315	6.1202E-02	0.7423	7.6161E-02	0.8165	2.0503E-01	0.5219	2.7589E-02	0.8219	7.6893E-01	0.8900	4.2408E-02	0.8136	4.6903E-02
	3	**0.4311**	6.6160E-02	0.6312	6.3380E-02	0.8094	5.5390E-02	0.8903	1.1814E-01	0.5363	2.1340E-02	0.9446	4.5270E-01	0.9704	7.9402E-02	0.9350	9.0201E-02
	4	0.5785	2.6365E-01	**0.5548**	3.8006E-02	0.8652	7.8029E-02	0.9516	1.0276E-01	**0.5548**	1.0729E-02	0.9682	7.9573E-01	1.0372	7.5407E-02	0.9584	8.5662E-02
	5	**0.4404**	1.0429E-01	0.7974	2.2139E-01	1.0382	9.3762E-02	1.1420	2.4321E-01	0.6679	1.0139E-02	0.9837	6.0668E-01	1.2447	5.9423E-02	0.9738	6.7505E-02
Test 9	2	0.5814	4.0773E-02	**0.5747**	3.4794E-01	0.4466	1.0239E-01	0.4913	9.3195E-02	0.6124	1.6911E-02	0.7452	5.8153E-02	0.5355	3.1984E-02	0.7376	3.3135E-02
	3	**0.5994**	2.8904E-02	0.6029	9.4721E-02	0.5045	1.9628E-01	0.5549	1.2631E-01	0.6341	1.7031E-02	0.7472	9.1132E-02	0.6048	3.6978E-02	0.7397	3.8309E-02
	4	0.5188	5.6489E-02	**0.4566**	4.1557E-01	0.6027	2.6702E-01	**0.4566**	1.6636E-01	0.6585	3.2251E-02	0.7911	1.1743E-01	0.7226	2.9131E-02	0.7831	3.4479E-02
	5	0.5332	1.9848E-02	**0.4842**	5.1341E-01	0.6898	2.2361E-01	0.7587	1.2510E-01	0.6787	2.8386E-02	0.8748	4.9614E-01	0.8270	3.1434E-02	0.8660	3.7205E-02
Test 10	2	**0.3844**	1.7914E-02	0.9624	3.1042E-01	0.7397	3.5095E-02	0.8136	1.3587E-01	0.4082	1.5582E-02	0.8474	1.3220E+00	0.8869	4.1000E-02	0.8388	4.8528E-02
	3	0.3418	3.0178E-02	0.9286	4.7516E-01	0.7943	2.1144E-02	0.8736	2.1161E-01	**0.3513**	5.5684E-02	0.9564	1.2279E+00	0.9523	2.7447E-02	0.9467	3.2486E-02
	4	0.4190	8.2430E-03	0.9657	8.4410E-02	0.8262	2.1362E-02	0.9088	3.8729E-01	**0.3815**	6.2569E-02	0.9658	6.8974E-01	0.9905	4.3848E-02	0.9560	5.0660E-02
	5	**0.4238**	5.8797E-02	0.9911	1.7805E-01	0.9468	8.3050E-01	1.0414	1.4083E-01	0.5098	7.4769E-02	0.9882	4.0178E-01	1.1352	6.8562E-02	0.9782	7.9214E-02

Significant values are in bold.

**Table 9:** Mean and STD values of the optimal fitness obtained by Otsu’s method.

Test Image	nTh	IHBO		HBO		SSA		MFO		GWO		SCA		HS		EMO	
		Mean	STD	Mean	STD	Mean	STD	Mean	STD	Mean	STD	Mean	STD	Mean	STD	Mean	STD
Test 1	2	**2194.885**	5.7134E-13	2131.370	5.2000E-12	1958.607	3.2500E-12	1964.426	9.2000E-13	1604.246	8.0200E-02	1964.402	8.2900E-13	1963.413	9.2300E-13	1964.417	1.6600E-02
	3	**2260.382**	4.0653E-06	2194.972	3.7000E-05	2123.712	1.4600E-02	2131.265	5.7800E-03	1604.246	5.6000E-01	2129.935	4.6100E-13	2111.747	0.0000E+00	2131.372	4.1600E-02
	4	**2286.649**	2.7735E-05	2220.479	2.8100E-02	2184.805	4.0400E-02	2194.973	3.4900E-02	1601.951	1.8900E+01	2437.504	2.3100E-12	2179.246	9.3000E-03	2194.947	2.0500E-01
	5	**2307.986**	7.8450E-03	2241.198	7.1400E-02	2208.524	4.6100E-02	2220.834	9.8000E-03	1600.293	1.4400E+01	2587.055	1.3500E+00	2215.602	1.2300E-02	2219.391	4.3100E-01
Test 2	2	2221.765	7.6033E-14	2157.472	6.9200E-13	2430.503	6.9200E-13	**2537.504**	6.9200E-13	2034.527	8.2500E-02	2437.504	6.9200E-13	2437.871	6.9200E-13	2437.504	6.9200E-13
	3	2256.541	1.0141E-13	2191.242	9.2300E-13	2580.323	2.5000E-02	**2588.330**	9.2300E-13	2039.244	8.2000E-01	2587.583	9.2300E-13	2582.915	4.6100E-13	2588.300	1.3700E-01
	4	**2777.255**	2.1865E-03	2696.888	1.9900E-02	2646.375	4.1400E-02	2657.473	1.5000E+01	2044.527	2.9300E+01	2653.467	4.6100E-13	2638.716	1.3800E-02	2657.472	2.1700E-01
	5	**2796.418**	6.6584E-01	2715.496	6.0600E+00	2686.608	6.8200E-01	2696.804	7.3500E+00	2058.534	2.0500E+01	2653.351	5.7800E-01	2696.471	7.6900E-01	2696.889	1.4400E+00
Test 3	2	**1692.447**	0.0000E+00	1643.472	0.0000E+00	1546.032	0.0000E+00	1552.457	0.0000E+00	1222.792	1.2000E-01	1552.457	0.0000E+00	1543.807	0.0000E+00	1552.457	0.0000E+00
	3	1747.897	4.3950E-04	**1757.917**	4.0000E-03	1637.029	5.3100E-02	1643.472	6.9200E-13	1242.797	2.8900E+01	1697.317	6.9200E-13	1628.171	2.9000E-03	1643.472	3.8400E-02
	4	1744.546	1.5798E-05	**1755.063**	1.6000E-02	1654.125	6.2200E-02	1697.017	1.7200E-02	1649.755	2.3900E+01	1693.695	2.8900E-04	1679.165	9.1000E-03	1697.317	1.3000E+00
	5	1792.750	5.3320E-05	1740.872	5.4000E-02	1712.485	5.3300E-01	**1803.288**	4.3300E+00	1685.315	2.0200E+01	1692.815	2.3000E-03	1711.761	6.0000E-02	1723.199	1.0500E+00
Test 4	2	**3838.491**	1.5163E-14	3727.414	1.3800E-12	3641.278	1.3800E-12	3651.867	1.3800E-12	3067.814	4.2000E-02	3651.613	1.3800E-12	3830.909	9.2300E-13	3651.867	1.2000E-02
	3	3895.112	1.4284E-05	3782.397	1.3000E-03	3716.225	2.3700E-02	3727.414	2.3100E-12	3208.087	5.7600E-01	3727.232	2.3100E-12	**3895.142**	1.3800E-12	3727.371	5.1300E+00
	4	**3925.344**	4.6037E-04	3911.754	4.1900E-02	3769.229	6.8300E-02	3782.398	1.5100E-02	3258.632	1.5700E+00	3778.105	9.1000E-03	3774.971	7.4200E-02	3782.398	1.2200E+00
	5	3985.776	9.2184E-04	**3987.697**	8.3900E-02	3800.264	1.1600E-01	3813.742	1.3300E-02	3295.114	1.9100E+01	3806.778	6.0900E-04	3833.697	3.5100E-02	3805.724	5.0000E+00
Test 5	2	2000.114	1.0141E-14	**2025.432**	9.2300E-13	1942.973	8.0000E-03	1949.294	9.2300E-13	1974.575	3.6900E-02	1949.234	9.2300E-13	1947.463	4.6100E-13	1949.293	9.2300E-13
	3	2045.033	8.7899E-06	2070.919	8.0000E-04	**2118.918**	4.2000E-02	2025.433	0.0000E+00	2002.571	5.6400E-01	**2118.918**	0.0000E+00	2012.874	8.4900E-04	2025.433	6.8500E-02
	4	2134.909	1.3734E-04	**2196.931**	1.2500E-02	2061.398	7.4600E-02	2070.527	5.0000E-03	2059.734	1.8400E+01	2125.228	4.6100E-13	2067.908	7.4000E-03	2070.926	1.5100E+00
	5	**2150.180**	4.8345E-02	2111.259	4.4000E+00	2086.816	8.0000E-02	2096.967	6.1300E+00	2098.914	1.4700E+01	**2150.180**	3.7000E-03	2050.263	1.6900E-02	2096.935	6.7300E-01
Test 6	2	2690.368	0.0000E+00	**2724.423**	0.0000E+00	2527.085	0.0000E+00	2534.441	3.6400E-02	2236.247	9.4000E-02	2534.441	0.0000E+00	2534.492	0.0000E+00	2534.441	2.8500E-02
	3	**2891.223**	0.0000E+00	2824.839	0.0000E+00	2714.836	4.0300E-04	2724.423	1.7000E-03	2448.361	2.4400E+01	2723.995	0.0000E+00	2696.862	0.0000E+00	2723.047	1.7900E-02
	4	**2943.991**	1.0987E-04	2876.396	1.0000E-02	2811.446	4.6000E-03	2824.843	4.5600E-02	2625.646	2.9000E+01	2823.443	9.9800E-04	2824.348	1.3800E-12	2824.208	2.3800E-02
	5	**2975.808**	7.6622E-05	2907.482	7.7600E-02	2863.994	1.7500E-01	2876.308	4.4600E-02	2650.884	2.4200E+01	2828.659	8.3000E-03	2875.682	3.2900E-02	2876.308	1.3800E-01
Test 7	2	**1807.663**	1.7690E-14	1760.103	1.6100E-12	1623.129	1.6100E-12	1627.909	1.6100E-12	1606.711	8.9100E-02	**1807.663**	1.6100E-12	1627.294	1.6100E-12	1627.909	5.2600E-02
	3	**1871.842**	1.0141E-14	1828.864	9.2300E-13	1753.192	3.7900E-04	1760.103	1.9000E-03	1740.822	2.1600E+01	1857.629	9.2300E-13	1760.981	1.3800E-12	1760.103	3.0800E-01
	4	1879.158	1.7360E-04	1871.971	1.5800E-02	1813.172	5.3300E-02	1828.792	5.6600E-02	1801.213	2.7600E+01	**1897.867**	1.1500E-12	1827.486	9.2300E-13	1828.307	1.7400E-01
	5	1901.635	4.7691E-05	**1907.029**	4.8300E-02	1859.064	4.2300E-01	1871.924	5.3400E-02	1846.239	2.3700E+01	1824.237	4.5000E-03	1865.092	2.8700E-02	1871.971	7.3800E-02
Test 8	2	**3288.010**	6.8328E-16	3212.516	6.9200E-13	3053.809	6.9200E-13	3063.526	6.9200E-13	2501.315	6.1000E-02	3263.475	6.9200E-13	3063.343	6.9200E-13	3063.475	6.9200E-13
	3	3345.355	1.5163E-13	3268.544	1.3800E-12	3200.527	1.3800E-12	3212.517	1.4100E-01	2650.271	9.6000E+00	**3350.259**	1.3800E-12	3212.577	6.9200E-13	3212.517	8.3000E-02
	4	**3383.481**	4.7685E-01	3305.795	4.3400E+00	3253.889	2.0200E-02	3268.741	6.3200E-02	2674.957	1.6600E+01	3367.113	1.2700E-02	3268.748	1.1500E-12	3268.497	1.4700E-01
	5	**3403.903**	4.1422E-01	3325.748	3.7700E+00	3291.844	1.9600E-01	3307.331	3.7800E+00	2690.816	1.1400E+01	3400.386	2.5000E-03	3285.643	9.4000E-03	3307.331	2.6900E-01
Test 9	2	1650.510	0.0000E+00	**1671.403**	0.0000E+00	1551.144	0.0000E+00	1555.731	0.0000E+00	1530.216	5.0400E-02	1555.691	0.0000E+00	1552.766	0.0000E+00	1555.691	0.0000E+00
	3	**1753.172**	2.5381E-13	1712.918	2.3100E-12	1655.822	6.0300E-02	1671.403	1.1900E-04	1610.256	2.8700E+01	1669.475	2.3100E-12	1657.173	1.3800E-12	1671.403	4.1300E-02
	4	**1779.966**	3.8456E-03	1739.097	3.5000E-02	1700.282	2.5600E-01	1713.252	6.7100E-05	1706.167	3.1900E+01	1669.737	1.3800E-12	1701.348	3.9600E-06	1713.127	2.4500E-01
	5	**1797.314**	4.1972E-03	1756.047	3.8200E-02	1727.451	6.4500E-01	1736.528	4.1400E-01	1733.115	1.9400E+01	1706.363	4.5400E-02	1710.863	3.9700E-01	1759.022	2.7300E-01
Test 10	2	1679.010	7.6033E-14	1640.459	6.9200E-13	**1679.369**	9.4000E-03	1542.907	6.9200E-13	1454.615	2.0700E-01	1542.899	6.9200E-13	1540.462	1.1500E-12	1642.899	1.1300E-02
	3	**1742.790**	7.6033E-14	1702.775	6.9200E-13	1740.314	6.8000E-03	1640.459	2.6000E-03	1457.515	2.7000E+01	1639.327	6.9200E-13	1636.417	1.9600E+01	1640.459	2.0300E-02
	4	**1787.985**	1.1553E-05	1731.011	1.1700E-02	**1787.985**	1.8600E-02	1702.775	9.8600E+00	1587.387	2.9600E+01	1702.246	9.2300E-13	1677.415	3.4000E-03	1742.775	9.5100E-01
	5	1787.292	1.2979E-02	1746.255	3.3400E+01	**1789.766**	1.5900E+01	1731.011	2.0700E+01	1721.601	1.5200E+01	1722.038	2.0100E-01	1712.117	1.6800E-03	1757.986	6.2000E-12

Significant values are in bold.

**Table 10:** Mean and STD values of PSNR results obtained by Otsu’s method.

Test Image	nTh	IHBO		HBO		SSA		MFO		GWO		SCA		HS		EMO	
		Mean	STD	Mean	STD	Mean	STD	Mean	STD	Mean	STD	Mean	STD	Mean	STD	Mean	STD
Test 1	2	**19.0524**	2.2436E-03	17.4279	2.0400E-02	15.4016	1.8500E-12	15.4016	2.4800E-13	15.7527	6.5800E-02	15.2431	2.5800E-13	15.0441	1.1400E-12	14.2494	3.1400E-02
	3	**20.5241**	9.2384E-04	18.7742	8.4000E-03	17.4279	1.4500E-02	18.7742	1.2700E-02	15.7523	1.9980E-01	17.2103	2.3500E-02	17.2318	8.7000E-03	15.9879	4.1500E-02
	4	18.1574	4.2673E-03	19.4801	3.8800E-02	18.7742	3.9200E-02	18.7742	4.1500E-02	16.7526	6.0230E-01	**20.0341**	7.2100E-15	18.1213	1.5200E-02	17.9861	1.1150E-01
	5	19.2599	4.2233E-04	20.6629	3.8400E-02	**20.6851**	2.0500E-02	19.4028	3.0200E-02	18.7529	8.8760E-01	19.2071	1.5260E-01	20.2814	2.1500E-02	20.2616	1.1380E-01
Test 2	2	18.0705	7.9296E-04	**18.3592**	7.2100E-02	16.2997	7.2100E-15	16.2997	7.2100E-15	16.4912	3.1600E-02	15.4016	7.2100E-15	15.4016	7.2100E-15	15.4085	1.6200E-02
	3	**21.6706**	1.1878E-04	20.7376	1.0800E-02	18.3592	1.6500E-02	18.3592	1.0800E-14	17.2917	6.4410E-01	17.4279	1.0800E-14	17.4279	1.0800E-14	17.4333	4.4300E-02
	4	**22.3900**	4.2893E-05	22.3104	3.9000E-03	20.7376	5.8000E-03	20.7376	2.3680E-01	18.2907	6.2630E-01	18.7742	0.0000E+00	18.7752	2.5000E-03	18.7771	4.2300E-02
	5	**23.3803**	3.1103E-03	23.2163	2.8280E-01	23.3104	3.2150E-01	22.2854	2.0580E-01	19.2977	5.3970E-01	19.5116	2.6470E-01	19.7961	3.6460E-01	19.5624	3.1610E-01
Test 3	2	**18.3590**	1.3858E-03	17.7084	1.2600E-02	15.4217	1.2600E-14	15.4217	1.2600E-14	15.4087	4.9600E-02	17.7117	1.2000E-13	15.4217	1.2600E-14	15.3238	1.1100E-02
	3	20.0802	3.9593E-01	20.1976	3.6000E-03	17.7084	6.3400E-02	17.7084	7.2100E-15	17.9785	7.4620E-01	**20.2084**	7.2100E-15	17.7089	2.6000E-03	17.7589	8.2900E-02
	4	21.4252	3.9703E-03	21.4279	3.6100E-02	20.1976	5.0100E-02	**21.4758**	4.1400E-02	19.7216	1.4092E+00	20.2001	1.4800E-02	20.2119	3.2000E-02	20.2669	9.7700E-02
	5	**23.4331**	8.3036E-02	23.2646	7.5500E-02	21.5676	1.9760E-01	21.7046	2.4100E-01	20.5034	3.2110E-01	21.5699	5.0000E-03	21.6749	1.0130E-01	21.6435	1.7910E-01
Test 4	2	20.0953	2.5626E-02	20.2114	2.3300E-02	**20.2474**	0.0000E+00	17.2474	2.6300E-02	18.8571	1.8300E-02	18.5476	3.1400E-02	18.9077	1.7300E-02	17.8087	6.7000E-03
	3	**21.6474**	3.9813E-03	21.5328	3.6200E-02	21.2113	9.1000E-03	20.2114	1.0800E-14	20.7496	1.1220E-01	20.3452	1.0800E-14	20.3442	1.0800E-14	20.3376	2.3900E-02
	4	23.0580	1.4078E-02	**23.4783**	1.2800E-02	21.5328	2.1700E-02	21.5328	8.0000E-03	21.9144	7.3410E-01	21.1737	5.0000E-04	21.1807	1.3100E-02	21.1741	2.8600E-02
	5	23.3517	2.9035E-02	23.7773	2.6400E-02	**23.7827**	1.7400E-02	23.2827	1.6200E-02	22.4174	7.3460E-01	23.6934	3.6000E-03	23.6814	1.3100E-02	23.6585	6.0200E-02
Test 5	2	**19.5379**	1.3858E-03	18.7868	1.2600E-02	15.0294	1.2400E-02	15.0295	1.2600E-14	18.0546	6.5100E-02	15.0347	1.2600E-14	15.1295	1.2600E-14	15.2374	2.6000E-02
	3	**21.6678**	2.8375E-03	20.7351	2.5800E-02	18.7868	6.1700E-02	18.7868	1.4400E-14	20.8075	1.3422E+00	18.7868	1.4400E-14	18.8143	2.7400E-02	18.8376	8.7900E-02
	4	**23.5257**	3.7833E-03	23.1664	3.4400E-02	20.7351	5.5700E-02	20.8801	1.9300E-02	23.1682	1.0403E+00	20.0359	1.0800E-14	21.3378	1.0000E-02	20.7131	1.1300E-01
	5	24.8769	3.9725E-02	24.5853	3.6120E-01	24.6603	3.8100E-02	24.6603	5.5720E-01	20.2984	9.2180E-01	22.1518	2.2900E-02	**24.9666**	3.0500E-02	22.0424	1.6140E-01
Test 6	2	**17.1204**	1.9577E-03	16.5754	1.7800E-02	13.9437	1.0800E-14	13.9437	1.4300E-04	15.4016	2.3400E-02	16.2997	1.0800E-14	16.2037	1.0800E-14	16.2878	1.1000E-03
	3	**19.6319**	1.5837E-04	18.8728	1.4400E-02	16.5753	4.6000E-03	16.5753	5.4200E-03	17.2654	3.4510E-01	18.3502	1.4400E-14	18.0481	1.4400E-14	18.1785	2.4700E-02
	4	20.6370	1.0976E-02	20.7069	9.9800E-02	18.8728	9.2000E-03	18.8728	2.4900E-02	19.5449	9.5150E-01	**20.7371**	2.6000E-03	20.7076	1.0800E-14	20.3278	4.0600E-02
	5	**23.3362**	2.2986E-03	22.2612	2.0900E-02	20.5396	1.7000E-02	20.5396	1.2800E-02	20.7012	1.0633E+00	22.3144	1.5000E-03	22.2041	1.1100E-02	22.1665	4.6600E-02
Test 7	2	**18.8937**	5.9610E-01	18.1975	5.4200E-02	15.9994	5.4100E-15	15.9994	5.4100E-15	16.3202	2.4500E-02	15.7494	5.4100E-15	15.9351	5.4100E-15	16.0148	3.6000E-03
	3	20.6004	7.9296E-04	**20.6734**	7.2100E-02	20.1974	9.4000E-03	18.1974	2.1800E-02	17.0135	6.8190E-01	18.1774	7.2100E-15	18.1684	7.2100E-15	18.1068	3.4000E-02
	4	22.2613	2.1006E-04	22.1927	1.9100E-02	**22.6734**	1.1100E-02	20.6829	2.9500E-02	19.6592	1.1060E+00	20.6734	1.8000E-14	20.6734	1.8000E-14	20.6658	5.2000E-02
	5	**23.9678**	3.7174E-03	23.7537	3.3800E-02	22.2254	2.6400E-02	22.2736	3.1300E-02	20.3591	9.3050E-01	22.3205	7.7000E-03	22.2565	5.2000E-03	22.1581	7.9100E-02
Test 8	2	**20.5438**	4.0253E-03	20.3472	3.6600E-02	20.3472	2.9800E-02	17.8874	0.0000E+00	14.9097	0.0000E+00	14.6065	0.0000E+00	14.6291	7.6000E-03	14.6071	0.0000E+00
	3	**22.3472**	3.9593E-03	22.1504	3.6000E-02	**22.3472**	3.6000E-15	20.3472	1.3000E-01	17.0926	1.8104E+00	19.1571	3.6000E-15	19.1531	3.6000E-15	19.1178	1.6760E-01
	4	**23.6265**	8.3586E+00	23.4415	7.6000E-02	23.5732	7.2400E-02	22.1732	1.4060E-01	17.8655	1.9656E+00	21.1671	5.0800E-02	21.1802	7.2100E-15	20.5465	2.5600E-01
	5	24.2248	1.9753E-02	**24.7531**	1.7960E-01	24.6945	2.5100E-02	23.6945	3.8600E-01	20.2017	1.8785E+00	21.4112	2.2800E-02	22.2684	3.4700E-02	22.1879	1.7990E-01
Test 9	2	16.5384	1.4847E-03	16.9578	1.3500E-02	13.6937	1.3200E-02	**16.9978**	1.3500E-02	14.9607	2.6500E-02	13.9573	1.4200E-02	15.9581	1.4200E-02	13.9576	1.6400E-02
	3	18.6995	1.1922E-02	18.9346	1.0840E-01	16.9578	1.6960E-01	**19.0346**	8.0000E-03	15.3148	1.0297E+00	16.5753	1.0800E-14	18.5753	1.0800E-14	16.7093	1.8240E-01
	4	19.5683	2.3866E-03	**19.7293**	2.1700E-02	19.1112	1.2830E-01	19.1112	2.1500E-02	16.1401	1.0999E+00	18.8728	7.2100E-15	**19.7293**	6.4000E-03	18.9318	1.1340E-01
	5	**20.8438**	6.3019E-02	20.7638	5.7300E-02	19.7293	1.6300E-01	20.1234	9.1200E-02	18.3409	8.7270E-01	20.5658	3.0300E-02	**20.8438**	1.3940E-01	20.5454	1.2450E-01
Test 10	2	19.9428	9.8323E-03	**19.9572**	8.9400E-02	19.6091	2.6800E-02	14.6091	0.0000E+00	19.1375	7.7500E-02	**19.9572**	0.0000E+00	13.6937	0.0000E+00	13.7163	4.5900E-02
	3	21.1545	5.0591E-03	21.1803	4.6000E-02	**21.2571**	2.0000E-04	19.1571	4.0600E-02	21.3725	1.1890E+00	21.9578	0.0000E+00	16.8803	4.5820E-01	16.9389	7.6600E-02
	4	**22.9984**	7.0168E-03	22.3993	6.3800E-02	21.3803	2.9700E-02	21.1803	5.1600E-01	22.8746	1.1509E+00	22.1112	1.0800E-14	19.1148	2.0100E-02	19.1497	1.1920E-01
	5	**23.6993**	1.0206E-02	23.1291	9.2800E-02	**23.6993**	1.4700E-01	22.3993	7.5300E-02	23.3993	9.4460E-01	**23.6993**	2.2000E-02	19.7688	5.8100E-02	19.7809	1.3630E-01

Significant values are in bold.

**Table 11:** Mean and STD values of FSIM results obtained by Otsu’s method.

Test Image	nTh	IHBO		HBO		SSA		MFO		GWO		SCA		HS		EMO	
		Mean	STD	Mean	STD	Mean	STD	Mean	STD	Mean	STD	Mean	STD	Mean	STD	Mean	STD
Test 1	2	**0.7771**	1.0269E-05	0.7536	1.0000E-04	0.6984	3.3800E-16	0.6984	3.4000E-16	0.5984	1.2000E-02	0.7611	3.3800E-16	0.7011	3.0500E-16	0.7111	4.2000E-03
	3	0.7936	1.6225E-03	0.8036	1.5800E-02	0.7536	2.0000E-04	0.75647	2.3800E-05	0.5531	5.9000E-03	**0.8157**	2.2500E-16	0.8107	2.3500E-16	0.8044	2.1000E-02
	4	0.8230	9.2418E-05	**0.8333**	9.0000E-04	0.8036	6.0000E-04	0.8036	7.6500E-04	0.6301	1.4800E-02	0.8316	5.6300E-16	0.8275	3.0000E-04	0.8062	2.4000E-03
	5	0.8857	5.1344E-05	0.8589	5.0000E-04	0.8322	2.0000E-04	0.8329	4.0000E-04	0.6541	1.8400E-02	0.8459	2.1000E-03	**0.8862**	2.0000E-04	0.8436	1.7000E-03
Test 2	2	**0.8054**	2.1359E-03	0.7810	2.0800E-02	0.7244	3.3800E-16	0.7044	3.3800E-16	0.6996	1.8000E-03	0.6974	3.3800E-16	0.6923	3.4300E-16	0.5908	1.3000E-03
	3	**0.8490**	2.3105E-03	0.8233	2.2500E-02	0.7811	7.0000E-04	0.7817	2.2500E-16	0.7494	1.9000E-02	0.7906	2.2500E-16	0.7436	2.2500E-16	0.7244	1.7000E-03
	4	**0.8843**	2.0537E-04	0.8575	2.0000E-04	0.8633	4.0000E-04	0.8233	1.0900E-02	0.7863	2.2500E-02	0.8036	3.3800E-16	0.8036	2.0000E-04	0.8037	1.0000E-03
	5	0.8876	4.9290E-03	**0.8881**	4.8000E-03	0.8575	2.7000E-03	0.8584	6.8000E-03	0.8035	1.6200E-02	0.8342	4.0000E-04	0.8336	1.6000E-03	0.8802	1.5000E-03
Test 3	2	0.8856	1.5608E-02	**0.8882**	1.5200E-02	0.8452	1.0300E-16	0.8452	1.1900E-16	0.8448	1.6000E-03	**0.8882**	1.0700E-16	0.8482	1.5100E-16	0.8458	3.6000E-03
	3	0.9223	4.1075E-04	0.9235	4.0000E-04	0.8879	9.0000E-04	0.9235	7.8900E-16	0.8834	1.2600E-02	**0.9279**	7.8900E-16	0.8878	3.0000E-04	0.8876	1.0000E-03
	4	**0.9499**	5.1344E-04	0.9406	5.0000E-04	0.9235	7.0000E-04	0.9426	6.0000E-04	0.9055	2.1800E-02	0.9290	2.0000E-04	0.9237	4.0000E-04	0.9243	1.6000E-03
	5	0.9547	1.9511E-03	0.9549	1.9000E-03	0.9398	1.0000E-03	0.9395	2.7000E-03	0.9309	1.7800E-02	0.9398	1.3000E-03	0.9404	2.1000E-02	**0.9609**	2.1000E-03
Test 4	2	0.8101	6.6849E-02	**0.8147**	6.5100E-02	0.7711	4.5100E-16	0.7711	2.0100E-16	0.7975	4.0000E-04	0.7902	3.1500E-16	0.7932	4.5100E-16	0.7951	2.1000E-03
	3	0.8371	2.1872E-02	**0.8476**	2.1300E-02	**0.8476**	2.0000E-04	0.8147	5.6300E-16	0.8025	7.0000E-04	0.8735	5.6300E-16	0.8735	5.6300E-16	0.8734	4.0000E-04
	4	0.9130	3.0806E-05	0.8854	3.0000E-04	0.8476	4.0000E-04	0.8476	2.7300E-04	0.8386	1.5900E-02	**0.9122**	9.0000E-05	0.9118	3.0000E-04	0.9117	2.0000E-03
	5	0.9225	2.0537E-06	0.8946	2.0000E-04	0.8863	1.0000E-04	0.8863	5.1800E-05	0.8851	1.4700E-02	**0.9382**	6.4700E-05	0.9362	1.1000E-03	0.9377	2.4000E-03
Test 5	2	**0.8500**	2.5672E-05	**0.8500**	2.5000E-03	0.8109	3.0000E-04	0.8129	2.2500E-16	0.8109	1.2000E-03	0.8171	2.2500E-16	0.8119	2.2500E-16	0.8102	7.0000E-04
	3	**0.9120**	9.2418E-06	0.8844	9.0000E-04	0.8589	2.1000E-03	0.8586	1.1300E-16	0.8477	1.5700E-02	0.8501	1.1300E-16	0.8505	1.0000E-03	0.8514	1.9000E-03
	4	**0.9465**	1.0269E-04	0.9179	1.0000E-03	0.8843	1.5000E-03	0.8847	5.4300E-04	0.8735	1.6800E-02	0.8853	3.3800E-16	0.8844	4.0000E-04	0.8856	2.1000E-03
	5	**0.9700**	4.3129E-04	0.9407	4.2000E-03	0.9178	1.9000E-03	0.9178	7.6000E-03	0.8785	1.4700E-02	0.9171	6.0000E-04	0.9191	2.6000E-03	0.9155	2.0000E-03
Test 6	2	**0.8595**	3.9021E-04	0.8335	3.8000E-03	0.7661	3.3800E-16	0.7662	5.0200E-05	0.7267	5.0000E-04	0.8335	3.3700E-16	0.7245	8.0000E-04	0.7204	6.4400E-07
	3	0.8748	3.9021E-04	**0.8858**	3.8000E-03	0.8335	8.1000E-05	0.8335	2.0900E-04	0.7796	9.9000E-03	0.8811	3.3800E-16	0.7818	3.3800E-16	0.7828	5.0000E-04
	4	0.9029	3.2860E-04	0.9142	3.2000E-03	0.8858	1.0000E-04	0.8858	2.9100E-04	0.8097	1.7300E-02	**0.9233**	6.2200E-05	0.8203	7.8900E-16	0.8205	1.7000E-03
	5	0.9251	1.3349E-04	**0.9367**	1.3000E-03	0.9362	5.0000E-04	0.9162	1.3500E-04	0.8255	2.0400E-02	0.8575	2.1900E-05	0.8576	2.2000E-03	0.8574	1.6000E-03
Test 7	2	**0.8355**	4.6312E-03	0.6163	4.5100E-02	**0.8355**	4.5100E-16	0.7572	4.5000E-16	0.7579	2.5000E-03	0.7502	4.0100E-16	0.7512	4.1100E-16	0.7522	1.1000E-03
	3	**0.9108**	2.5672E-04	0.8832	2.5000E-03	0.8288	1.4100E-05	0.8288	1.3600E-04	0.8274	2.2500E-02	0.8278	2.2500E-16	0.8284	2.2500E-16	0.8241	5.0000E-04
	4	**0.9401**	3.1833E-04	0.9117	3.1000E-03	0.8832	4.0000E-04	0.8837	6.3300E-04	0.8624	2.4400E-02	0.9130	3.3800E-16	0.8802	3.3800E-16	0.8825	7.0000E-04
	5	**0.9627**	1.4376E-04	0.9336	1.4000E-03	0.9109	9.0000E-04	0.9129	1.3000E-03	0.8893	2.2100E-02	0.9209	1.0000E-04	0.9116	7.0000E-04	0.9429	1.3000E-03
Test 8	2	**0.9009**	2.1154E-17	0.8736	2.0600E-16	0.7952	2.5100E-16	0.7952	2.3200E-16	0.7578	1.5000E-03	0.7573	2.2500E-16	0.7473	2.2500E-16	0.7674	2.2000E-03
	3	**0.9386**	4.6312E-16	0.9102	4.5100E-16	0.8735	4.5100E-16	0.8735	1.3000E-03	0.8667	3.0100E-02	0.8352	4.5100E-16	0.8356	4.5100E-16	0.8354	1.5000E-03
	4	**0.9469**	9.8580E-04	0.9376	9.6000E-03	0.9413	8.0000E-04	0.9113	9.0000E-04	0.8428	2.8200E-02	0.8986	5.0000E-04	0.8975	2.7000E-02	0.8987	1.8000E-03
	5	0.9523	4.4155E-04	0.9526	4.3000E-03	0.9372	1.3000E-03	0.9372	5.4000E-03	0.8839	2.6500E-02	**0.9581**	1.0000E-04	0.9286	1.1000E-03	0.9265	1.5400E-03
Test 9	2	**0.8038**	4.3129E-04	0.8029	4.2000E-03	0.7291	4.2200E-05	0.7291	4.3200E-05	0.7664	8.0000E-04	0.7662	4.5500E-05	0.7762	4.5000E-05	0.7662	1.0000E-04
	3	**0.8740**	2.5672E-04	0.8476	2.5000E-03	0.8029	1.4000E-03	0.8029	1.5700E-04	0.8256	2.6000E-02	0.8335	2.2500E-16	0.8335	2.2500E-16	0.8351	2.3000E-03
	4	**0.9129**	9.3445E-04	0.8853	9.1000E-03	0.8498	1.9000E-03	0.8498	2.0000E-04	0.8754	2.7000E-02	0.8858	2.0100E-02	0.8858	5.1100E-06	0.8872	2.2000E-03
	5	0.8886	1.7457E-04	0.8998	1.7000E-03	0.8782	3.4000E-03	0.8789	2.4000E-03	0.8914	1.5800E-02	**0.9154**	8.0000E-04	0.9148	5.0000E-04	0.9116	2.4000E-03
Test 10	2	0.8247	5.7809E-16	**0.8351**	5.6300E-16	0.7573	1.4000E-03	0.7573	5.6300E-16	0.7261	1.0000E-03	0.7291	5.6000E-16	0.7291	5.3300E-16	0.7275	6.0000E-04
	3	**0.9214**	5.7809E-16	0.8935	5.6300E-16	0.8357	1.3000E-03	0.8357	7.2500E-04	0.7896	2.4300E-02	0.8589	5.6300E-16	0.8015	1.2200E-02	0.8052	3.3000E-03
	4	0.9271	1.2322E-04	**0.9281**	1.2000E-03	0.9281	2.1200E-03	0.8935	1.1000E-02	0.8211	2.2700E-02	0.8491	3.0500E-16	0.8494	1.1000E-03	0.8511	3.1000E-03
	5	**0.9679**	4.5182E-04	0.9483	4.4000E-03	0.9581	3.7000E-03	0.9387	4.3200E-03	0.8734	2.2400E-02	0.8782	7.5400E-05	0.8702	3.4000E-03	0.8776	6.1000E-03

Significant values are in bold.

**Table 12:** Mean and STD values of SSIM results obtained by Otsu’s method.

Test Image	nTh	IHBO		HBO		SSA		MFO		GWO		SCA		HS		EMO	
		Mean	Std	Mean	Std	Mean	Std	Mean	Std	Mean	Std	Mean	Std	Mean	Std	Mean	Std
Test 1	2	0.6499	3.2010E-04	0.6279	3.1000E-03	0.6459	4.0100E-16	0.7679	4.5100E-16	0.2855	5.0000E-04	0.7679	3.0400E-16	0.7679	4.3100E-16	**0.7879**	4.4000E-03
	3	0.7011	1.2804E-04	0.6774	1.2400E-02	0.7218	8.0000E-04	0.6459	8.6200E-05	0.2858	9.5000E-03	**0.8166**	7.8900E-16	0.8006	7.8900E-16	0.8066	2.3000E-03
	4	0.6318	4.1304E-05	0.7028	4.0000E-04	0.7647	5.0000E-04	0.7262	3.9200E-04	0.6585	7.4000E-03	**0.8312**	4.5100E-16	0.8212	2.0000E-04	0.8103	1.7000E-03
	5	0.7759	1.9619E-04	0.7497	1.9000E-03	0.8089	5.2000E-03	0.7647	1.5000E-03	0.6912	1.2600E-02	0.8575	2.3000E-03	**0.8590**	1.3000E-03	0.8576	3.1000E-03
Test 2	2	**0.7683**	1.3424E-04	0.6554	1.3000E-03	0.7587	1.1300E-16	0.7587	1.1500E-16	0.6474	2.9000E-03	0.6559	1.1300E-16	0.6469	1.1300E-16	0.6473	1.4000E-03
	3	**0.8216**	5.2662E-04	0.7938	5.1000E-03	0.7928	1.2000E-03	0.7693	4.5100E-16	0.7095	2.5100E-02	0.7218	4.5100E-16	0.7218	4.5100E-16	0.7224	2.7000E-03
	4	**0.9006**	1.0326E-05	0.8701	1.0000E-04	0.8612	1.0000E-04	0.8614	6.3500E-03	0.7512	2.0300E-02	0.7647	4.5100E-16	0.7648	1.0000E-04	0.7647	1.6000E-03
	5	**0.9131**	2.0032E-03	0.8822	1.9400E-02	0.8840	1.0800E-02	0.8837	9.0000E-03	0.7698	1.7000E-02	0.7897	8.8000E-03	0.7993	1.2100E-02	0.7813	1.0500E-02
Test 3	2	0.7791	8.3640E-05	**0.7899**	8.1000E-03	0.7886	1.0300E-16	0.7886	1.1300E-16	0.7585	3.5000E-03	0.7886	1.1300E-16	0.7876	1.0100E-16	0.7887	5.0000E-04
	3	**0.8713**	2.0652E-04	0.8617	2.0000E-03	0.8574	1.5000E-03	0.8562	7.8900E-16	0.7312	2.0700E-02	0.8544	7.8900E-16	0.8522	3.0100E-05	0.8533	2.0000E-03
	4	**0.9147**	8.2607E-05	0.9138	8.0000E-04	0.9123	1.1000E-03	0.9109	9.0000E-04	0.8922	3.3500E-02	0.9101	3.0000E-04	0.9026	7.0000E-04	0.8288	2.1000E-03
	5	**0.9701**	2.1684E-04	0.9691	2.1000E-03	0.9327	2.5000E-03	0.9346	3.4000E-03	0.9038	2.6400E-02	0.9328	6.8400E-05	0.9343	0.0000E+00	0.9357	2.7000E-03
Test 4	2	**0.7953**	2.0652E-05	0.7684	2.0000E-04	0.7679	2.2500E-16	0.7679	2.2500E-16	0.6065	1.1000E-03	**0.7953**	2.2500E-16	0.6615	2.2500E-16	0.7582	6.0000E-04
	3	**0.8772**	8.2607E-05	0.8475	8.0000E-04	0.8066	1.0000E-03	0.8066	5.6300E-16	0.7169	3.6000E-03	0.8466	5.6300E-16	0.8465	5.6300E-16	0.8275	1.1000E-03
	4	**0.9189**	1.9619E-04	0.8878	1.9000E-03	0.8312	3.2000E-03	0.8312	6.0000E-04	0.7214	1.7000E-02	0.8886	3.0000E-04	0.8326	2.8000E-03	0.8875	2.5000E-03
	5	**0.9548**	3.0978E-05	0.9225	3.0000E-04	0.8584	4.0000E-04	0.8582	2.2900E-04	0.7761	1.4300E-02	0.9225	6.7300E-05	0.9212	2.1000E-03	0.9206	1.7000E-03
Test 5	2	0.8452	2.0652E-05	0.8166	2.0000E-04	**0.8492**	4.0000E-04	0.8292	2.2500E-16	0.7014	2.5000E-03	0.8382	2.2500E-16	0.8382	2.2500E-16	0.8396	1.0000E-03
	3	**0.9070**	1.6521E-04	0.9053	1.6000E-03	0.8894	3.7000E-03	0.8894	2.2500E-16	0.7869	2.0800E-02	0.8794	2.2500E-16	0.8513	1.7000E-03	0.8426	3.2000E-03
	4	0.9072	8.2607E-05	0.9075	8.0000E-04	0.9110	1.2000E-03	0.9112	5.4500E-04	0.8062	1.4900E-02	**0.9131**	4.5100E-16	0.9105	2.0000E-04	0.9099	2.1000E-03
	5	0.9162	5.1630E-04	**0.9459**	5.0000E-03	0.9457	7.0000E-04	0.9457	8.0000E-03	0.8176	1.3600E-02	0.9435	4.0000E-04	0.9405	5.0000E-04	0.9441	2.6000E-03
Test 6	2	0.5776	2.6847E-04	0.5836	2.6000E-03	0.6137	3.3800E-16	0.6137	2.8100E-04	0.5596	1.8000E-03	**0.7589**	3.3800E-16	0.7577	3.3800E-16	0.7546	5.0000E-04
	3	0.6836	3.6141E-03	0.6907	3.5000E-03	**0.7992**	2.5600E-05	0.7572	5.0000E-04	0.6077	7.7000E-03	0.7985	0.0000E+00	0.7979	1.0200E-02	0.7981	1.3000E-03
	4	0.7549	1.0326E-04	0.7628	1.0000E-04	0.8458	7.3100E-05	0.8478	4.0100E-04	0.6439	2.5500E-02	0.8312	1.8100E-05	0.8412	0.0000E+00	**0.8611**	1.9000E-03
	5	0.8201	3.0978E-04	0.8286	3.0000E-04	**0.8961**	5.0000E-04	0.8720	1.5800E-04	0.7691	2.5400E-02	0.8856	8.4300E-05	0.8841	1.2000E-03	0.8841	1.0700E-02
Test 7	2	0.8203	9.2933E-04	**0.8288**	9.0000E-04	0.7315	0.0000E+00	0.7316	0.0000E+00	0.7149	1.2000E-03	0.7310	2.0000E-04	0.7313	2.2000E-03	0.7309	3.0000E-04
	3	**0.8359**	1.0326E-04	0.8076	1.0000E-04	0.8006	1.0000E-04	0.8016	4.2800E-04	0.6385	2.3100E-02	0.8076	2.2500E-16	0.8056	2.2500E-16	0.8066	7.0000E-04
	4	**0.8879**	5.1630E-03	**0.8879**	5.0000E-03	0.8853	9.0000E-04	0.8876	1.6000E-03	0.7796	3.6900E-02	0.8849	5.6300E-16	0.8835	5.6300E-16	0.8759	1.8000E-03
	5	**0.9366**	1.4456E-03	0.9146	1.4000E-03	0.9281	1.4000E-03	0.9212	1.6400E-03	0.8942	2.6400E-02	0.9108	3.6100E-05	0.9174	9.0000E-04	0.9195	3.1000E-03
Test 8	2	0.7661	5.2662E-04	0.7015	5.1000E-03	**0.7684**	4.5100E-16	0.7584	4.3100E-16	0.6049	1.2000E-02	0.7012	4.5100E-16	0.7035	4.5100E-16	0.7014	2.0000E-04
	3	**0.8839**	1.2391E-04	0.8830	1.2000E-03	0.8465	2.3500E-16	0.8465	2.7700E-03	0.6443	3.1700E-02	0.8830	2.2500E-16	0.8726	2.6400E-16	0.8727	3.5000E-03
	4	**0.9250**	1.3424E-04	0.9227	1.3000E-03	0.8861	6.0000E-04	0.8861	2.0000E-03	0.7845	6.1400E-02	0.9026	4.0000E-04	0.9197	1.1300E-16	0.9018	3.3000E-03
	5	0.9315	2.6847E-04	**0.9419**	2.6000E-03	0.9222	3.0000E-04	0.9222	4.7000E-03	0.8944	4.2100E-02	**0.9419**	2.0000E-04	0.9317	3.0000E-04	0.9290	3.9000E-03
Test 9	2	0.5878	1.0326E-05	0.5679	1.0000E-04	0.5991	1.0000E-04	**0.5991**	3.0000E-04	0.5146	9.0000E-04	0.5137	1.0000E-04	0.52285	1.1000E-03	0.5128	1.2000E-03
	3	**0.7641**	2.5815E-04	0.6523	2.5000E-03	**0.7641**	8.0000E-03	0.7635	1.7300E-04	0.5483	5.7300E-02	0.6571	2.2500E-16	0.6532	2.2500E-16	0.6645	9.4000E-03
	4	**0.8882**	3.7173E-04	0.8582	3.6000E-03	0.8413	5.1000E-03	0.8419	3.6000E-04	0.6294	4.4200E-02	0.8434	4.5100E-16	0.8438	1.7700E-05	0.8481	4.6000E-03
	5	**0.8970**	3.0978E-05	0.8667	3.0000E-04	0.8567	5.3000E-03	0.8683	2.8500E-03	0.7585	2.9200E-02	0.8911	5.9600E-05	0.8952	4.4000E-03	0.8907	3.3000E-03
Test 10	2	**0.7165**	2.2097E-02	0.6923	2.1400E-02	0.7015	1.6000E-03	0.7005	1.1300E-16	0.5516	4.8000E-03	0.5991	1.1300E-16	0.53991	1.1300E-16	0.6505	2.8000E-03
	3	0.8799	1.5592E-03	**0.8801**	1.5100E-02	0.8641	3.0700E-05	0.8339	2.1700E-03	0.6494	5.9100E-02	0.7341	4.5100E-16	0.7504	1.0300E-02	0.7307	6.0000E-03
	4	0.8780	2.0652E-04	0.8896	2.0000E-03	**0.9229**	9.0000E-04	0.9228	1.8400E-02	0.7995	4.1300E-02	0.8431	5.6300E-16	0.8412	6.0000E-04	0.8323	4.0000E-03
	5	**0.9476**	2.2717E-04	0.9156	2.2000E-03	0.9412	2.0000E-04	0.9418	1.0000E-03	0.8187	3.1900E-02	0.8567	7.0000E-04	0.8571	1.8000E-03	0.8618	1.1000E-03

Significant values are in bold.

**Table 13:** Comparison of the *p*-values obtained through the Wilcoxon signed-rank test between the pairs of IHBO vs HBO, IHBO vs SSA, IHBO vs MFO, IHBO vs GWO, IHBO vs SCA, IHBO vs HS, and IHBO vs EMO for fitness results using Otsu’s method.

Test Image	nTh	HBO	SSA	MFO	GWO	SCA	HS	EMO
Test 1	2	1.532E-07	3.280E-01	3.280E-01	3.280E-01	1.390E-08	3.280E-01	3.280E-01
	3	2.303E-06	6.570E-02	4.840E-01	4.230E-01	2.090E-13	5.890E-03	1.430E-15
	4	1.510E-03	6.310E-01	1.300E-01	4.380E-01	1.370E-12	1.090E-07	1.510E-06
	5	7.098E-10	2.700E-05	2.850E-02	1.860E-03	6.440E-13	2.910E-05	2.130E-05
Test 2	2	6.635E-01	4.250E-01	4.250E-01	4.250E-01	6.020E-07	4.250E-01	4.200E-01
	3	2.561E-10	5.910E-03	3.650E-01	5.890E-03	2.260E-13	5.890E-03	1.380E-15
	4	6.119E-15	1.390E-03	4.290E-02	1.230E-04	5.400E-13	4.120E-08	3.280E-06
	5	1.036E-11	3.200E-03	4.100E-02	1.340E-07	9.140E-13	1.130E-06	4.300E-01
Test 3	2	NaN	NaN	NaN	NaN	2.610E-07	NaN	1.110E-16
	3	4.408E-04	5.230E-03	3.610E-01	3.670E-04	3.890E-13	3.670E-04	2.010E-03
	4	6.954E-14	1.700E-01	7.180E-02	4.580E-05	6.310E-12	1.310E-07	1.280E-01
	5	7.836E-15	6.52E-05	2.960E-01	7.280E-09	7.110E-13	1.400E-11	7.610E-05
Test 4	2	2.557E-05	1.580E-01	1.580E-01	1.580E-01	2.320E-04	1.590E-01	2.590E-16
	3	6.954E-02	1.220E-02	9.140E-01	3.010E-03	6.310E-11	3.010E-03	1.890E-15
	4	6.789E-12	4.250E-01	3.650E-01	1.510E-04	6.160E-10	1.790E-06	3.300E-01
	5	4.684E-01	4.610E-01	1.110E-03	4.290E-05	4.250E-09	1.420E-08	3.760E-10
Test 5	2	NaN	NaN	3.290E-01	NaN	2.460E-07	NaN	1.120E-16
	3	9.820E-15	2.180E-01	8.190E-02	7.340E-04	8.910E-13	7.420E-04	7.420E-05
	4	3.868E-12	9.880E-06	1.900E-03	3.870E-09	3.510E-10	8.290E-11	1.590E-05
	5	7.439E-15	8.890E-01	5.310E-02	7.130E-04	6.750E-13	1.490E-06	7.210E-02
Test 6	2	6.205E-06	1.580E-01	1.580E-01	9.630E-01	5.630E-04	1.580E-01	4.130E-13
	3	2.348E-05	3.640E-04	1.580E-03	2.590E-02	2.130E-13	3.590E-04	3.390E-15
	4	5.775E-04	1.120E-02	3.820E-06	6.860E-01	5.240E-13	1.280E-07	4.740E-02
	5	5.720E-04	4.300E-02	7.510E-01	2.690E-02	5.190E-13	3.120E-07	2.290E-06
Test 7	2	1.034E-06	8.140E-02	8.140E-02	8.140E-02	9.100E-06	8.140E-02	8.140E-02
	3	9.331E-13	3.510E-04	1.620E-03	3.330E-01	8.210E-13	3.550E-04	3.260E-15
	4	2.318E-04	4.530E-01	1.850E-01	2.360E-01	2.040E-13	7.560E-04	7.280E-04
	5	7.035E-14	1.820E-01	6.520E-02	8.480E-03	6.190E-13	1.610E-09	2.120E-07
Test 8	2	6.876E-08	NaN	NaN	NaN	6.050E-07	NaN	NaN
	3	2.182E-14	1.100E-02	1.100E-02	1.656E-03	1.920E-13	1.130E-02	1.430E-07
	4	2.784E-14	7.600E-02	3.450E-01	1.070E-01	2.450E-13	4.120E-03	1.380E-02
	5	7.421E-13	1.460E-01	1.410E-05	4.680E-01	6.530E-13	1.510E-13	4.510E-09
Test 9	2	NaN	NaN	NaN	NaN	1.640E-05	NaN	NaN
	3	5.239E-13	1.770E-04	2.720E-01	9.630E-04	4.610E-13	1.800E-04	4.230E-15
	4	6.756E-04	4.510E-07	4.710E-02	5.650E-07	6.130E-13	2.120E-09	5.080E-14
	5	5.279E-04	3.890E-01	3.560E-01	6.300E-01	4.790E-13	3.900E-01	4.040E-04
Test 10	2	2.215E-05	8.120E-02	6.580E-01	8.200E-02	2.010E-05	8.060E-02	3.900E-16
	3	2.425E-16	3.290E-01	1.050E+00	5.800E-02	2.200E-14	3.290E-01	1.120E-14
	4	1.929E-03	4.120E-02	8.570E-05	5.990E-03	1.750E-12	6.360E-07	6.790E-14
	5	7.572E-05	4.750E-03	1.890E-01	6.670E-08	6.870E-13	8.130E-11	1.390E-05

### Kapur results analysis

The best results are illustrated in Table 16, and were obtained by the IHBO using a fitness function such as Kapur entropy (14). Tables 14, and 15, present the histogram distribution of the benchmark images and segmented images with different numbers of thresholds produced by the IHBO. The results in Table 18 illustrate that the proposed algorithm with the Kapur entropy method proved outperform other algorithms in terms of SSIM (Table 18); in addition to, it outperformed other algorithms in terms of the mean FSIM (Table 20), PSNR (Table 19), and mean fitness.

The values of the computational time of comparison algorithms obtained by Otsu’s method are presented in Table 17. The IHBO came in first place with 24 cases in 40 experiments and proved its superiority in computational time compared to other competitive algorithms. HBO came in second place with 13 experiments, while GWO came in third place with ten experiments, followed by SSA with four experiments. Finally, the SCA came in fifth place with only one experiment, and the remaining algorithms could not obtain the best computational time in any of the experiments.

Table 18 presents the STD and average fitness results of the Kapur method on the benchmark images. The IHBO was in first place by obtaining optimal fitness values with 24 cases in 40 experiments. The SCA was in second place in seven experiments, while the HBO was in third place in five experiments. SSA was in fourth place in three experiments, and HS was in fifth place in two experiments followed by GWO in sixth place in one experiments. Finally, EMO and MFO could not produce optimal fitness values. Table 18 also presents the STD values to demonstrate the stability of the algorithm according to the repetition of the values.

Table 19 illustrates the STD and mean PSNR. The IHBO came in first place in 23 experiments with optimal PSNR values, while the SCA came in second place in ten experiments only. HBO came in third place in four experiments, while HS and GWO came in fourth place in two experiments. Finally, SSA, MFO, and EMO came in last place with no experiments. According to the STD values, EMO came in first place with the maximum number of minimum STD cases, followed by IHBO in second place, HS in third place, MFO and the HBO in fourth place, SSA in fifth place, and SCA in sixth place. GWO had no optimal STD values.

Table 20 provides the STD and mean FSIM. It can be observed that the IHBO was in first place in 17 experiments, while SCA was in second place in nine experiments. HS came in third place with six experiments, while MFO, HBO and GWO came in fourth place with three experiments, followed by SSA in fifth place with one experiment. Finally, EMO came in last place with no experiments. In terms of the STD, MFO came in first place with the maximum number of minimum STD cases, followed by SSA in second place, EMO and the IHBO in third place, HS in fourth place, HBO in fifth place, and SCA in sixth place. GWO had no optimal STD values.

The mean and STD of SSIM are presented in Table 21. The results indicate that IHBO was in first place in 19 experiments in terms of the SSIM, followed by SCA, which were in second place with seven experiments. The GWO and MFO were in third place in six experiments, while HBO and HS were in fourth place with three experiments, followed by SSA in fifth place with only one experiment. Lastly, EMO had no optimal experiments in terms of SSIM. According to the STD values, MFO came in first place with the maximum number of minimum STD cases. GWO, EMO, and SCA were in second place, while the IHBO was in third place. SSA and HBO were in fourth place, while HS was in fifth place.

Finally, Table 22 presents the *p*-values resulting from the Wilcoxon test for fitness using the Kapur fitness function. This table presents the difference between the proposed algorithm and the compared algorithms (HBO, SSA, MFO, GWO, SCA, HS, and EMO). The results in Table 22 indicate that the IHBO was different from the SCA and EMO but similar to the remaining algorithms. The exceptions occurred for nTH = 5, where in some cases the values exhibited differences as well as similarities (NaN values).

**Table 14:**
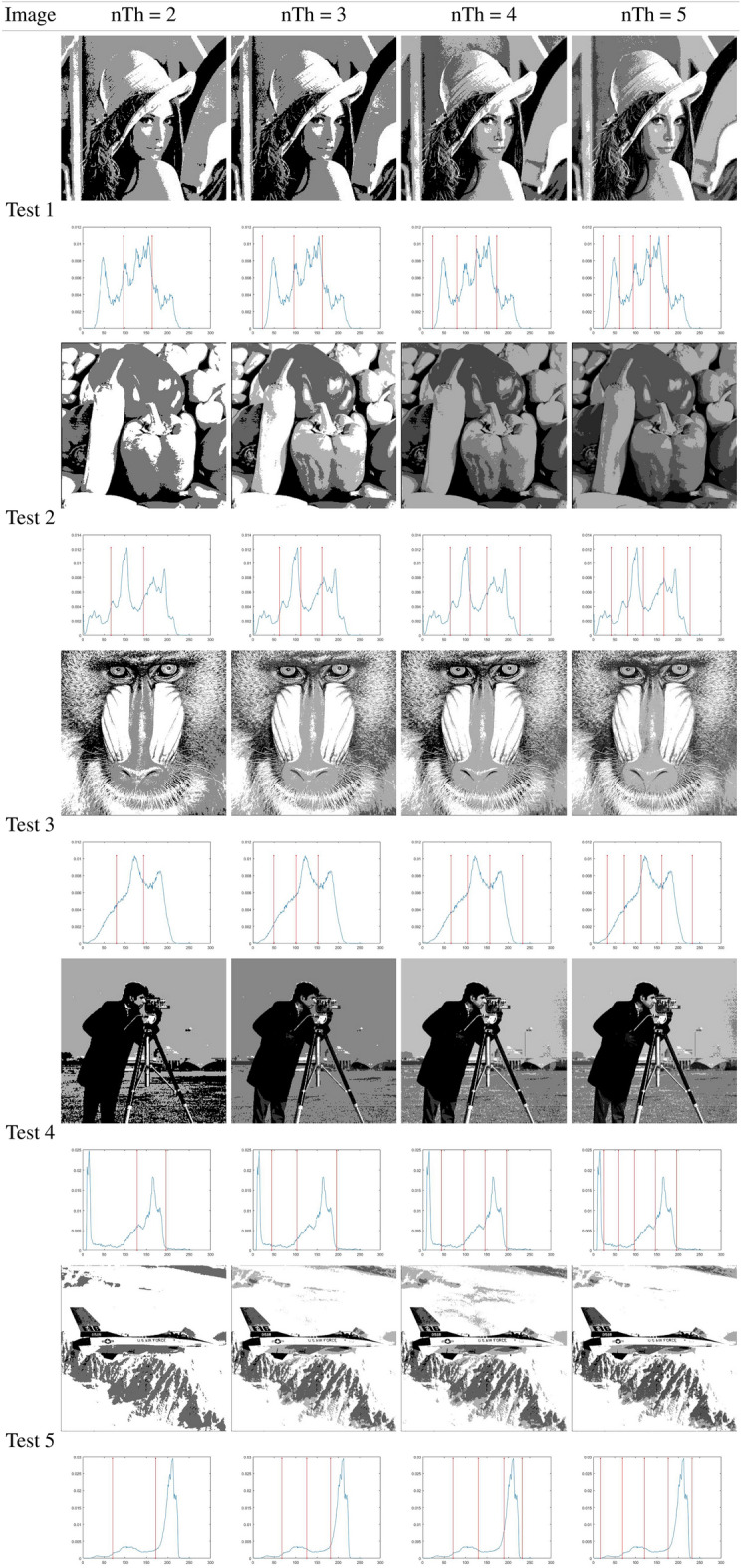
Implementation results of IHBO-Kapur over the set of test images.

**Table 15:**
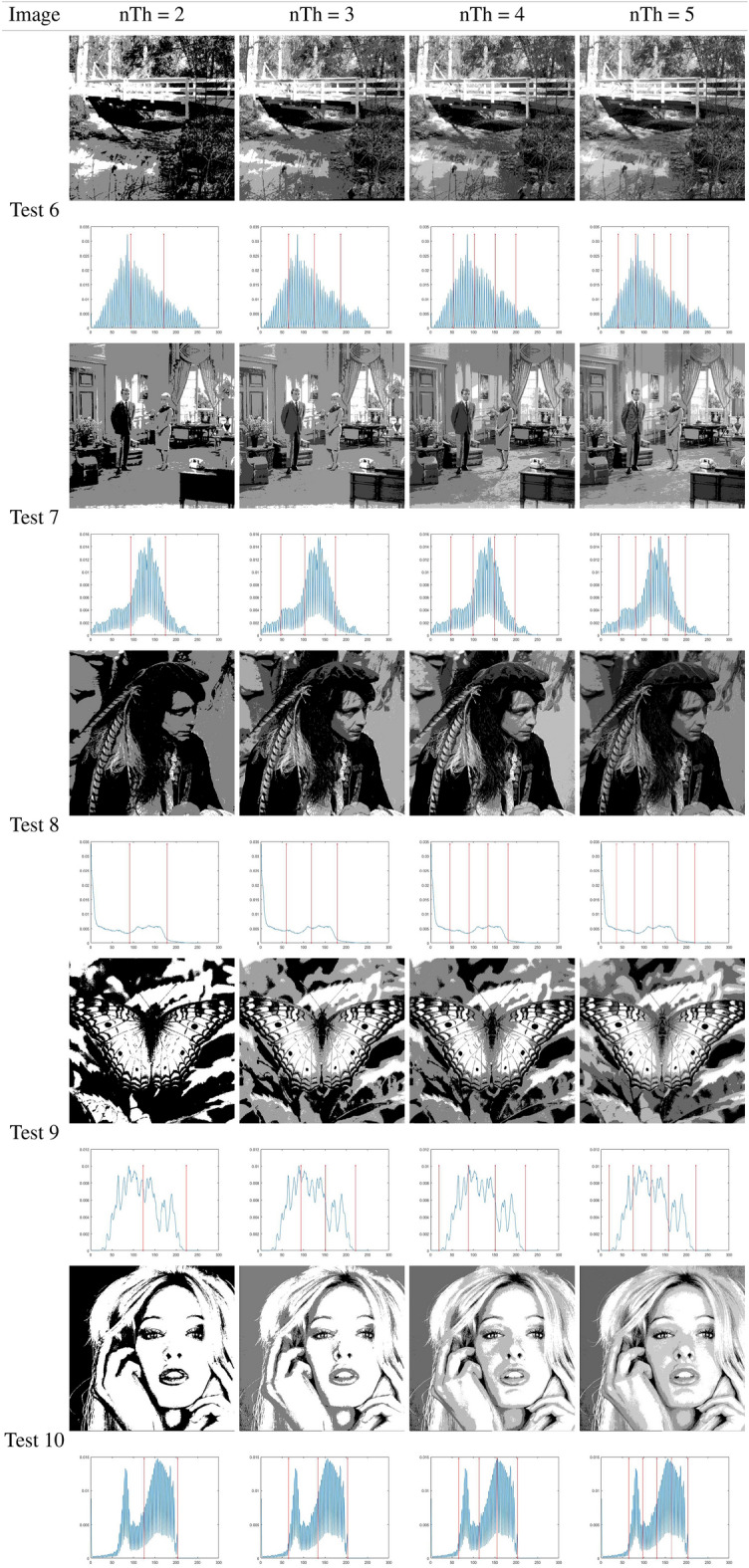
Implementation results of IHBO-Kapur over the set of test images.

**Table 16:** The best thresholds values obtained by Kapur’s method.

Test Image	nTh	IHBO	HBO	SSA	MFO	GWO	SCA	HS	EMO
Test 1	2	95 164	96 163	96 163	96 163	96 164	96 163	96 163	97 162
	3	24 96 164	23 96 163	23 96 163	23 96 163	23 109 161	23 96 163	23 96 163	23 95 164
	4	23 80 125 173	23 80 125 173	23 80 125 173	23 80 125 173	23 74 130 184	23 80 125 173	23 80 125 173	47 48 139 140
	5	23 62 94 135 177	23 77 119 159 190	23 62 94 135 177	23 62 94 135 177	21 76 113 135 166	23 71 109 144 180	23 71 109 144 180	23 111 115 119 138
Test 2	2	66 143	66 143	66 143	66 143	67 140	66 143	66 143	66 143
	3	61 111 161	62 112 162	62 112 162	62 112 162	58 108 159	62 112 162	62 112 162	55 108 159
	4	61 111 161 227	62 112 162 227	62 112 162 227	61 111 161 227	65 124 172 223	48 88 131 175	62 112 162 227	65 122 172 220
	5	45 85 125 169 227	61 108 144 179 227	61 109 152 196 227	45 85 128 173 227	33 62 102 169 227	41 79 110 167 227	51 90 146 196 227	30 62 100 169 228
Test 3	2	79 143	79 143	79 143	79 143	80 142	79 143	79 143	79 143
	3	78 143 233	79 143 233	49 101 153	49 101 153	47 103 157	49 101 153	49 101 153	49 100 153
	4	49 102 151 233	49 101 153 233	33 73 113 159	49 101 153 233	36 91 154 234	33 73 113 159	33 73 114 160	36 91 155 234
	5	33 73 113 160 233	33 73 115 161 233	33 73 113 159 233	33 72 115 159 233	29 59 107 166 237	33 69 104 138 172	33 69 104 138 172	33 69 104 135 170
Test 4	2	128 196	128 196	128 196	128 196	128 196	124 196	128 196	124 196
	3	44 103 196	44 103 196	44 103 196	44 103 196	42 99 198	44 103 196	41 103 197	42 100 198
	4	44 96 146 196	44 96 146 196	44 96 146 196	44 96 146 196	44 89 135 196	44 95 143 196	42 94 143 196	43 95 143 195
	5	24 60 98 146 196	44 96 145 192 217	24 60 98 146 196	23 60 98 145 196	34 74 100 141 195	31 81 118 154 195	27 81 118 154 195	34 72 100 142 195
Test 5	2	70 171	70 171	70 171	70 171	70 169	70 171	70 170	70 171
	3	68 126 182	68 126 182	68 126 182	68 126 182	70 131 182	68 126 182	69 126 182	66 126 182
	4	69 115 163 232	68 125 182 232	68 126 182 232	68 125 182 232	68 131 183 232	63 97 136 183	63 97 136 185	69 131 183 230
	5	58 100 138 182 233	64 104 143 184 232	64 104 144 185 232	63 102 142 184 232	14 52 102 153 186	16 64 104 143 185	16 60 104 145 185	17 60 105 145 184
Test 6	2	94 171	94 171	94 171	94 171	96 171	94 171	94 171	94 171
	3	65 126 187	65 126 187	65 126 187	65 131 195	68 129 191	57 122 187	56 122 187	68 129 190
	4	53 98 147 199	53 102 151 203	53 102 151 199	53 98 147 199	51 88 142 190	53 105 145 196	50 105 145 195	53 105 145 197
	5	40 81 122 167 207	45 89 131 171 207	45 85 131 171 211	40 85 131 171 211	40 84 124 181 218	40 77 114 155 203	42 78 114 155 201	42 78 112 155 204
Test 7	2	94 175	94 175	89 170	94 175	94 171	89 170	89 170	89 170
	3	47 103 175	50 103 175	47 103 175	47 103 175	47 102 172	47 103 175	45 104 176	40 104 176
	4	47 98 149 197	47 99 153 197	46 98 145 197	46 98 149 197	48 100 156 195	46 98 145 195	40 98 142 195	39 97 143 195
	5	42 85 124 162 197	42 85 124 162 197	42 85 124 162 197	42 85 124 162 197	45 80 126 164 194	46 79 113 149 189	43 79 112 149 185	23 80 124 164 196
Test 8	2	91 179	91 179	91 179	91 179	92 179	91 179	91 179	91 179
	3	60 118 179	60 118 179	60 118 179	60 118 179	61 122 178	60 118 179	62 118 180	62 118 181
	4	45 90 134 181	45 90 134 181	45 90 134 181	44 90 134 181	40 92 140 183	44 89 133 180	40 89 130 182	39 92 140 184
	5	45 88 132 179 220	44 89 133 179 220	44 89 133 179 220	41 86 130 179 220	47 87 119 145 184	33 69 105 141 181	30 69 104 140 182	30 69 102 141 183
Test 9	2	125 226	124 222	94 151	124 222	120 222	124 222	124 222	124 222
	3	92 154 226	94 151 222	94 151 222	94 151 222	95 154 222	114 155 223	113 154 223	114 152 220
	4	19 93 150 222	19 82 142 222	19 94 151 222	73 114 157 222	69 106 151 226	89 129 158 226	87 126 158 225	68 106 150 226
	5	19 74 115 157 222	19 74 116 158 222	19 74 115 157 222	61 95 135 174 222	63 92 127 184 224	19 73 113 155 222	20 70 113 155 225	18 70 113 155 223
Test 10	2	124 204	125 203	125 203	125 203	125 204	109 206	125 204	124 204
	3	66 133 204	65 134 203	65 134 203	65 134 203	60 146 205	65 134 203	64 133 203	66 132 203
	4	65 113 155 203	65 113 155 203	65 113 155 203	65 113 155 203	64 116 164 207	65 106 147 203	62 104 147 201	61 104 145 203
	5	65 98 131 166 202	65 100 134 168 203	65 113 155 203 229	65 97 131 166 203	65 98 146 177 203	65 120 155 203 224	63 123 155 200 225	67 98 145 177 220

**Table 17:** The computational time values of comparison algorithms obtained by Kapur’s method.

Test Image	nTh	IHBO		HBO		SSA		MFO		GWO		SCA		HS		EMO	
		Mean	STD	Mean	STD	Mean	STD	Mean	STD	Mean	STD	Mean	STD	Mean	STD	Mean	STD
Test 1	2	**0.6211**	3.2201E-01	0.6836	7.0752E-01	0.7857	5.3643E-01	0.6930	3.1476E-01	0.6549	3.1269E-01	0.6528	6.4282E-01	0.6422	2.6673E-01	0.6527	3.1368E-01
	3	**0.5093**	4.3560E-02	0.5254	1.0785E-01	0.6369	2.1898E-01	0.6749	1.2499E-01	0.7524	1.0979E-01	0.6887	3.6489E-01	0.6254	1.1956E-01	0.6886	1.4060E-01
	4	**0.5634**	5.0861E-02	0.9697	2.7778E-01	0.6134	1.7778E-01	0.6296	5.1239E-02	0.6121	3.1738E-02	0.7898	9.4604E-02	0.5834	3.9039E-02	0.7898	4.5911E-02
	5	**0.5653**	5.2965E-02	0.9085	1.0697E-01	0.6058	2.0219E-01	0.6571	1.3897E-01	0.6398	4.3965E-02	0.7935	8.0408E-02	0.6090	5.1128E-02	0.7934	6.0127E-02
Test 2	2	0.7779	3.9933E-02	0.8627	3.0127E-01	0.7142	2.5375E-01	0.8898	9.0833E-02	**0.6877**	1.3219E-02	0.9225	2.6054E-01	0.8899	1.2951E-01	0.9965	1.5230E-01
	3	0.9947	1.2210E-01	0.8889	1.2891E-01	0.9523	3.9247E-01	1.0086	1.9930E-01	**0.6890**	1.1056E-02	0.9347	2.5006E-01	1.0087	4.4735E-01	0.9281	5.2608E-01
	4	0.8699	1.6830E-02	0.8588	1.8140E-01	0.9645	8.1876E-01	0.9650	1.2422E-01	**0.8503**	1.3099E-02	0.9623	2.0064E-01	0.9651	1.6877E-01	0.9554	1.9003E-01
	5	**0.7865**	1.2499E-02	0.8645	9.5719E-02	0.9652	8.5805E-01	1.0000	9.5602E-02	**0.7865**	1.5981E-02	0.9771	1.6492E-01	1.0002	4.6753E-01	0.9702	5.2643E-01
Test 3	2	0.6717	1.1017E-02	**0.6617**	1.4953E-01	0.8472	5.8609E-01	0.8830	6.5657E-02	0.8550	2.6329E-02	0.9388	2.8028E-01	0.8831	2.9681E-01	0.8628	3.0682E-01
	3	0.6306	9.4267E-03	**0.6216**	9.8255E-02	0.8025	5.2226E-01	0.9609	1.0703E-01	0.8747	1.8732E-02	0.9235	2.3095E-01	0.9610	1.5853E-01	0.8488	1.6387E-01
	4	**0.5589**	9.4267E-03	**0.5589**	3.0645E-01	0.8296	4.7428E-01	1.1662	2.8681E-01	0.9157	5.3339E-01	**0.5589**	1.5967E-01	1.1663	1.0969E-01	0.9181	1.1339E-01
	5	**0.5909**	2.6733E-02	**0.5909**	2.2728E-01	0.9605	9.9428E-01	0.9246	1.0181E-01	0.9706	3.9691E-02	0.9635	1.5405E-01	0.9247	1.6923E-01	0.8855	1.7494E-01
Test 4	2	0.4941	7.1552E-02	**0.4641**	1.9497E-01	0.8276	5.2441E-01	0.8285	4.4177E-02	0.5565	6.3007E-02	0.8104	2.8270E-01	0.7678	2.7930E-02	0.7435	2.9292E-02
	3	**0.4993**	3.6084E-02	**0.4993**	1.2151E-01	0.8512	5.1131E-01	0.8736	7.2636E-02	0.5781	3.4843E-02	0.7371	1.4383E-01	0.8096	3.8484E-02	0.6763	4.0360E-02
	4	0.5497	3.6538E-02	**0.5390**	1.1557E-01	0.9829	6.7516E-01	0.8855	4.0486E-02	**0.5390**	1.2968E-02	0.8286	2.6200E-01	0.8206	3.1534E-02	0.7602	3.3073E-02
	5	0.5363	9.1571E-02	**0.5562**	2.3257E-01	1.2723	1.4614E+00	0.8818	5.5846E-02	0.6361	2.6198E-02	0.7207	1.9359E-01	0.8172	1.1146E-01	0.6612	1.1690E-01
Test 5	2	0.7228	3.6311E-02	**0.6647**	7.3112E-02	0.8580	5.3215E-01	0.8007	4.4415E-02	0.9133	1.4802E-02	0.7437	3.2203E-01	0.7420	7.6839E-02	0.6755	8.6981E-02
	3	**0.6075**	2.2467E-02	0.7664	2.1040E-01	0.8947	8.0661E-01	0.8520	5.4253E-02	0.9414	1.1582E-02	0.9284	4.7313E-01	0.7895	6.5899E-02	0.9200	7.4597E-02
	4	**0.6222**	5.9007E-03	**0.6222**	6.0431E-02	1.0681	8.9282E-01	0.8611	7.2294E-02	0.9758	1.6728E-02	1.0533	4.2204E-01	0.7980	2.0362E-01	1.0437	2.3049E-01
	5	**0.6001**	5.3445E-02	0.6913	7.6421E-02	1.1622	8.4519E-01	0.8857	7.1385E-02	1.0463	6.6057E-02	1.0656	1.6732E-01	0.8208	2.8715E-01	1.0559	3.2505E-01
Test 6	2	0.4836	2.5644E-02	0.7428	3.6359E-02	0.9585	1.8394E-01	1.2662	7.5695E-01	**0.4436**	1.5728E-02	0.9087	3.3413E-01	1.1734	5.4196E-02	0.9005	5.9941E-02
	3	0.5435	5.6509E-02	0.6535	7.3002E-02	0.8827	6.4412E-01	1.0701	3.3902E-01	**0.5279**	2.3592E-02	0.8868	2.6062E-01	1.0807	1.7295E-01	0.8787	1.9128E-01
	4	0.5504	2.1446E-02	**0.5395**	8.7401E-02	0.6977	3.0568E-01	0.8458	2.1584E-01	**0.5395**	1.6157E-02	0.9269	3.2188E-01	0.8542	6.3354E-02	0.9186	7.0070E-02
	5	**0.5677**	2.4963E-02	0.6194	1.0559E-01	0.7069	1.2637E-01	0.8570	7.8913E-02	0.6364	5.8908E-02	0.8848	1.0022E-01	0.8654	1.2911E-01	0.8768	1.4280E-01
Test 7	2	0.5455	3.7339E-02	0.5939	1.2669E-01	**0.4780**	3.6799E-02	0.5795	1.4316E-01	0.5105	1.6504E-02	0.8684	1.9612E-01	0.6316	4.5877E-02	0.9387	5.0740E-02
	3	**0.4883**	2.8560E-02	0.5818	1.1185E-01	0.5335	3.4044E-02	0.6467	1.8119E-01	0.6021	1.2470E-01	0.9573	3.0246E-01	0.6984	1.1541E-01	1.0349	1.2764E-01
	4	**0.5849**	1.5257E-01	0.6108	1.9344E-01	0.7632	1.6912E-01	0.9253	1.0241E-01	0.8054	1.8470E-02	0.9063	1.0705E-01	0.9993	4.0029E-02	0.9798	4.4271E-02
	5	**0.5458**	1.8743E-01	0.5655	4.1189E-02	1.0039	1.0460E-01	1.2502	6.7362E-02	0.8526	4.7710E-01	0.9619	2.3782E-01	1.3143	1.3659E-01	1.0399	1.5107E-01
Test 8	2	0.5915	1.3711E-01	**0.5551**	6.2732E-02	0.5924	7.9398E-02	0.8981	2.2399E-01	0.7822	3.0141E-02	0.9030	8.2909E-01	0.8908	4.2758E-02	0.8217	4.7290E-02
	3	**0.5216**	7.4430E-02	0.5548	6.4964E-02	0.6459	5.7744E-02	0.9793	1.2907E-01	0.8038	2.3314E-02	1.0378	4.8812E-01	0.9713	8.0057E-02	0.9443	9.0945E-02
	4	0.6999	2.9661E-01	**0.5630**	3.8956E-02	0.6904	8.1345E-02	1.0467	1.1226E-01	0.8315	1.1721E-02	1.0638	8.5800E-01	1.0382	7.6029E-02	0.9679	8.6369E-02
	5	**0.5329**	1.1733E-01	0.7009	2.2693E-01	0.8285	9.7747E-02	1.2561	2.6571E-01	1.0010	1.1077E-02	1.0808	6.5415E-01	1.2458	5.9913E-02	0.9834	6.8062E-02
Test 9	2	0.4615	4.5870E-02	0.7689	3.5663E-01	**0.4458**	1.0675E-01	0.5404	1.0088E-01	0.5355	1.8306E-02	0.8187	6.2703E-02	0.5408	3.4623E-02	0.8039	3.5065E-02
	3	**0.4833**	3.2517E-02	0.7936	9.7089E-02	0.5035	2.0462E-01	0.6104	1.3673E-01	0.5638	1.8436E-02	0.8210	9.1884E-02	0.6645	4.0029E-02	0.8062	4.0540E-02
	4	**0.5012**	6.3550E-02	0.7529	4.2596E-01	0.5015	2.7837E-01	0.7292	1.8009E-01	0.5955	3.4912E-02	0.8691	1.1840E-01	0.7939	3.1534E-02	0.8534	3.6487E-02
	5	**0.5241**	2.2329E-02	0.7772	5.2625E-01	**0.5241**	2.3312E-01	0.8345	1.3417E-01	0.6217	3.0728E-02	0.9612	5.0023E-01	0.9086	3.4027E-02	0.9438	3.9372E-02
Test 10	2	**0.4651**	2.0153E-02	0.8459	3.1818E-01	0.7235	3.6587E-02	0.8950	1.4572E-01	**0.4651**	1.8426E-02	0.9310	1.3329E+00	0.8877	4.1338E-02	0.8471	5.6208E-02
	3	**0.4015**	3.3950E-02	0.8162	4.8704E-01	0.7768	2.2043E-02	0.9610	2.2695E-01	**0.4015**	6.5846E-02	1.0508	1.2380E+00	0.9532	2.7673E-02	0.9561	3.7627E-02
	4	**0.5070**	9.2734E-03	0.8488	8.6520E-02	0.8080	2.2270E-02	0.9996	4.1537E-01	0.5698	7.3988E-02	1.0611	6.9543E-01	0.9915	4.4210E-02	0.9654	5.8677E-02
	5	**0.5128**	6.6147E-02	0.8712	1.8250E-01	0.9260	8.6580E-01	1.1455	1.5104E-01	0.5150	8.8414E-02	1.0858	4.0510E-01	1.1363	6.9128E-02	0.9879	9.1750E-02

Significant values are in bold.

**Table 18:** Mean and STD values of the optimal fitness obtained by Kapur’s method.

Test Image	nTh	IHBO		HBO		SSA		MFO		GWO		SCA		HS		EMO	
		Mean	STD	Mean	STD	Mean	STD	Mean	STD	Mean	STD	Mean	STD	Mean	STD	Mean	STD
Test 1	2	**18.1417**	3.4934E-18	17.8095	3.4500E-15	17.8096	3.4500E-15	17.7866	3.4000E-15	17.8096	3.9000E-15	17.8033	3.4000E-15	17.8019	2.2500E-15	17.8033	2.0300E-15
	3	**22.7497**	3.6453E-14	22.3331	3.6000E-15	22.3330	3.6000E-15	22.2716	5.1900E-16	22.3330	3.6000E-15	22.1292	1.8100E-16	22.0993	2.6500E-15	22.0568	3.1300E-15
	4	26.3699	2.3492E-03	26.6193	2.3200E-03	26.6193	2.3200E-03	26.5518	2.1200E-03	26.6123	6.1300E-04	**26.7491**	1.4100E-03	26.7421	2.1200E-03	26.7431	5.3100E-03
	5	30.3417	2.3694E-03	**30.5028**	2.3400E-02	30.5017	1.9800E-02	30.4099	2.3100E-02	30.3931	1.0400E-02	30.5023	2.3100E-02	29.6273	4.1100E-02	30.1110	1.3500E-02
Test 2	2	**18.5056**	3.4731E-17	18.1667	3.4300E-16	18.1665	3.4200E-16	18.1665	3.4300E-16	18.1665	3.2200E-16	18.1279	3.5500E-14	18.0279	3.4200E-15	17.2279	1.6300E-15
	3	**23.0357**	3.3516E-14	22.6139	3.3100E-13	22.6097	1.1900E-01	22.5809	4.7100E-02	22.6098	3.1300E-13	22.9852	8.1100E-12	22.9028	6.3300E-13	21.6341	3.7700E-14
	4	**27.5598**	1.6303E-03	27.0551	1.6100E-03	27.0543	1.5300E-04	27.0461	1.1100E-01	27.0576	6.7700E-03	27.0879	6.1100E-03	26.6845n	3.1100E-04	25.3889	1.9600E-04
	5	**32.2244**	4.5870E-01	31.3268	4.5300E-02	31.0742	1.1000E-02	31.0122	1.0600E-03	31.0743	1.3200E-02	31.3268	1.4900E-02	31.0123	7.4100E-02	31.0016	5.2300E-02
Test 3	2	18.1469	3.6453E-14	17.6414	3.6000E-15	**18.6413**	3.4200E-15	17.6141	3.6000E-15	17.6411	2.1300E-15	17.6255	1.2200E-15	17.6255	3.5600E-15	17.6251	6.3200E-12
	3	22.6997	4.1921E-01	22.0674	4.1400E-02	**23.0662**	2.3000E-02	22.0418	1.1400E-03	22.0668	5.8000E-03	22.0384	3.1600E-02	22.0391	1.7000E-03	22.0398	5.1200E-02
	4	27.3529	3.4731E-01	26.5910	3.4300E-02	26.5910	1.9800E-02	26.5910	4.7000E-03	26.5689	3.2200E-02	**27.5168**	3.5200E-03	26.1369	1.3600E-02	26.1437	5.2300E-03
	5	**31.9289**	1.2353E-01	30.6514	1.2200E-02	30.7193	1.9600E-01	30.6091	8.3200E-03	30.4081	1.0300E-02	**31.9289**	2.5500E-03	29.9288	5.3600E-03	29.9106	1.6900E-03
Test 4	2	**18.0880**	2.1568E-14	17.5841	2.1300E-15	17.5834	1.1200E-15	17.5518	3.2200E-07	17.5832	1.8900E-15	17.5558	1.1200E-14	17.5558	1.1600E-14	12.5772	2.1200E-16
	3	22.6379	2.5414E-05	22.0073	2.5100E-05	22.0054	1.0800E-05	21.9724	1.2200E-05	**22.8691**	2.3100E-05	21.9481	1.0800E-05	21.9685	1.1000E-05	17.5558	3.6800E-12
	4	**27.3481**	3.4526E-04	26.5863	3.4100E-04	26.5626	4.9100E-05	26.5324	4.1600E-04	26.5812	3.1600E-04	26.5364	1.5800E-04	26.5264	1.9300E-04	21.9685	1.9800E-05
	5	31.1370	3.2605E-02	**31.5668**	3.2200E-03	30.5469	2.2000E-03	30.4892	1.4700E-03	30.5541	2.9600E-03	30.5839	8.2300E-03	30.5023	4.5700E-03	30.5051	3.2900E-03
Test 5	2	17.9628	6.1970E-14	17.6339	6.1200E-15	**17.9811**	3.3200E-15	17.6312	3.6000E-15	17.6312	3.4100E-15	17.6174	2.6400E-15	17.6174	2.6400E-15	17.4348	1.3200E-15
	3	22.8002	6.6425E-14	22.3827	6.5600E-14	22.3813	1.0800E-14	22.3470	1.8100E-14	22.3824	5.2300E-14	**22.8676**	1.3100E-14	22.3674	1.1800E-14	22.3674	1.1800E-14
	4	**27.3774**	2.2581E-03	26.9820	2.2300E-03	25.9676	2.5200E-04	26.8941	1.6600E-04	26.9672	2.2000E-03	26.4152	2.0500E-03	26.9524	2.9800E-02	26.9525	3.0200E-02
	5	**31.3049**	2.3897E-02	30.8528	2.3600E-02	30.6169	2.1500E-02	30.7064	1.3900E-02	30.7652	2.2600E-02	30.9643	2.8500E-02	30.9089	2.1200E-02	30.9936	3.0100E-02
Test 6	2	15.9637	3.5643E-14	**15.9831**	3.5200E-15	15.7306	3.0100E-15	15.7312	3.2000E-15	15.7132	3.4100E-15	17.8130	6.2100E-15	17.8119	5.0100E-15	17.8051	6.2100E-15
	3	**22.8561**	3.6858E-03	22.5260	3.6400E-03	19.5334	1.3200E-03	19.5331	1.1800E-03	19.5332	1.2000E-03	22.4568	2.6000E-03	22.0993	2.1400E-03	22.4298	5.4100E-02
	4	23.2270	3.6554E-03	22.8915	3.6100E-04	22.8903	2.9800E-04	22.8927	3.9400E-04	22.8903	2.6900E-04	**26.7491**	5.2200E-02	26.7421	3.2600E-02	26.7431	4.2000E-02
	5	26.3719	1.7113E-02	25.9399	1.6900E-03	25.9323	1.1200E-03	25.9225	1.1000E-03	25.9179	1.0500E-03	30.5388	3.5900E-02	**30.6273**	3.8900E-02	30.6113	3.2300E-02
Test 7	2	18.1966	3.5438E-15	17.8985	3.5000E-15	17.8981	3.3800E-15	17.8981	3.3800E-15	17.8981	3.3800E-15	**18.2279**	6.5200E-15	18.2099	6.5200E-15	18.2171	6.5200E-15
	3	22.1188	1.8834E-13	22.4376	1.8600E-14	22.4377	2.7800E-14	22.4377	2.7800E-14	22.4377	2.7800E-14	**22.9852**	3.6800E-14	22.9828	2.9800E-14	22.6343	2.6400E-14
	4	**27.9917**	3.6048E-02	27.6491	3.5600E-03	26.6383	2.3600E-03	26.6481	2.1500E-03	26.6481	2.1500E-03	27.6491	6.1500E-03	26.6845	3.2300E-03	27.3889	2.1300E-03
	5	**32.1399**	4.9515E-01	31.6134	4.8900E-02	30.5136	3.2300E-03	30.5124	3.1000E-03	30.5076	2.8900E-03	31.5273	2.9900E-02	31.3923	2.5200E-03	31.5016	3.0900E-02
Test 8	2	**18.2946**	2.0049E-13	17.9949	1.9800E-14	17.8949	1.3600E-14	17.8949	1.3600E-14	17.8949	1.3600E-14	17.9949	1.3600E-14	17.6255	1.3600E-14	17.6251	1.0900E-14
	3	**22.9549**	3.1998E-04	22.5789	3.1600E-04	22.5790	3.2600E-04	22.5793	3.5200E-04	22.5791	3.1300E-04	22.0384	3.4600E-05	22.0391	3.9800E-05	22.0398	4.2300E-05
	4	**27.3468**	2.3289E-02	26.8988	2.3000E-03	26.7988	2.1300E-03	26.7988	2.1300E-03	26.7984	2.3600E-04	26.5168	1.1600E-03	26.1369	2.1300E-04	26.1437	2.6800E-04
	5	**31.3574**	2.6530E-01	30.8437	2.6200E-02	30.8458	2.9600E-02	30.8456	2.6400E-02	30.8132	6.6400E-03	29.9289	2.7400E-03	29.9288	2.6400E-03	29.9106	2.1400E-03
Test 9	2	**17.9633**	6.0856E-13	17.7739	6.0100E-14	17.7614	5.5400E-14	17.7614	4.4300E-14	17.7614	3.6400E-14	17.5558	4.0100E-14	17.5558	4.0100E-14	12.5772	4.7400E-14
	3	**22.4930**	2.1467E-02	22.2559	2.1200E-03	22.4500	2.5100E-03	22.4503	2.5100E-03	22.4503	2.5100E-03	22.4503	2.1200E-03	21.9685	2.1900E-03	17.5558	2.9800E-05
	4	26.3234	3.6959E-03	**26.6969**	3.6500E-03	26.6878	2.0100E-03	26.6031	1.8900E-03	26.6019	1.2400E-03	26.5364	1.5600E-04	26.5264	1.0900E-04	21.9685	1.7600E-04
	5	30.4194	4.1536E-04	**30.8510**	4.6300E-03	30.4339	4.2200E-03	30.8509	4.5400E-03	30.8505	4.4100E-03	30.5839	4.0300E-03	30.5023	3.6400E-03	30.5051	3.3300E-03
Test 10	2	**17.9739**	1.2201E-12	17.7844	1.3600E-12	17.7841	6.1900E-12	17.7841	6.1900E-12	17.7841	6.1900E-12	17.6174	0.0000E+00	17.6174	1.2100E-03	17.4348	2.4200E-03
	3	**22.6818**	3.1795E-03	22.4427	3.1400E-04	22.4421	1.4200E-04	22.4421	1.4200E-04	22.4421	1.4200E-04	22.4427	3.1200E-05	22.3674	3.0200E-04	22.3674	3.0200E-04
	4	**26.9854**	3.3516E-01	26.4635	3.3100E-02	26.4609	1.8900E-02	26.4636	3.4300E-02	26.4605	1.7400E-02	26.4152	2.1000E-03	26.9524	2.8900E-01	26.9525	1.3500E-01
	5	**31.3269**	4.1009E-01	30.9967	4.0500E-01	30.1383	2.3600E-01	30.1514	2.6900E-01	30.1919	3.1300E-01	30.9967	3.8900E-01	**31.3269**	2.4600E-01	30.9936	2.8800E+00

Significant values are in bold.

**Table 19:** Mean and STD values of PSNR results obtained by kapur’s method.

Test Image	nTh	IHBO		HBO		SSA		MFO		GWO		SCA		HS		EMO	
		Mean	Std	Mean	Std	Mean	Std	Mean	Std	Mean	Std	Mean	Std	Mean	Std	Mean	Std
Test 1	2	**15.0365**	8.7289E-14	14.6383	8.2100E-13	14.6373	6.1100E-13	14.6382	9.0100E-15	14.6383	4.5100E-15	13.9227	8.5200E-02	14.6383	1.1900E-01	14.6363	6.2000E-12
	3	**17.8278**	3.4341E-14	17.3557	3.2300E-13	15.2182	3.4500E-14	16.2169	5.9700E-10	16.2189	5.2600E-11	17.2013	1.2400E-03	17.3557	5.4100E-15	16.2189	3.9300E-08
	4	19.2329	3.3172E-05	**19.2869**	3.1200E-04	19.2866	3.0200E-04	19.2864	4.5600E-02	19.2846	4.4300E-13	17.4863	3.7800E+00	19.2860	1.8200E-10	19.2859	2.9300E-01
	5	20.8324	2.1583E-04	20.8909	2.0300E-03	19.8789	2.0900E-04	20.7909	9.4300E-14	**21.1109**	3.2000E-02	17.7789	3.3300E-02	20.9807	1.8900E-10	20.4476	3.6500E-08
Test 2	2	**17.2214**	3.6255E-13	16.7654	3.4100E-12	16.2656	3.6900E-10	16.2654	5.4100E-15	16.4459	5.4100E-15	14.7513	1.1000E+00	16.2655	5.4100E-15	16.2655	6.3220E-15
	3	**18.8670**	5.3266E-09	18.36738	5.0100E-08	18.36735	4.2100E-06	18.36718	5.7300E-01	16.4601	3.0300E-04	18.3863	3.4100E+00	18.3674	4.6100E-01	18.3674	9.3000E-01
	4	18.9566	3.6149E-12	18.4546	3.4000E-11	16.3546	3.9700E-10	18.4096	5.3100E-14	17.3097	3.0200E-09	**20.5965**	5.0700E-11	**20.5965**	1.4700E-01	19.8412	6.0700E-14
	5	**21.3556**	3.6149E-05	20.7901	3.4000E-04	19.4901	3.0900E-06	20.7819	2.1900E-06	19.1754	1.6900E-14	17.6409	3.0900E-02	19.8529	2.4900E-06	19.8412	3.5900E-01
Test 3	2	16.4529	3.2534E-15	16.0172	3.0600E-15	16.0171	3.1700E-15	16.0172	3.6000E-15	15.1254	2.1000E-08	**16.6230**	2.2800E+00	16.0493	3.6000E-15	16.0293	1.6300E-03
	3	16.3862	4.6356E-04	15.9523	4.3600E-04	15.9423	3.1200E-05	15.9468	1.7500E-03	17.5652	3.0900E-15	16.89951	3.3600E+00	**18.7642**	2.2100E-01	18.6642	3.5600E-01
	4	**19.2745**	3.2428E-02	18.7641	3.0500E-02	17.7641	2.1500E-02	18.7255	5.9200E-10	19.0701	2.3200E-12	17.1838	2.3100E+00	18.8583	5.6200E-09	18.5823	1.2200E-02
	5	**20.9717**	4.1040E-02	20.4164	3.8600E-01	20.3369	3.4500E+00	20.4144	2.0200E-05	20.4742	6.2000E+01	17.4752	6.32E6-02	17.6935	6.2000E-06	17.2931	2.0000E-02
Test 4	2	**13.9962**	6.3898E-17	13.6256	6.0100E-16	13.6251	5.4100E-10	13.5356	5.8900E-16	13.6419	7.2100E-15	13.6419	2.1200E-01	13.6257	9.0100E-15	13.6312	8.3200E-09
	3	14.8534	2.6580E-07	14.4601	2.5000E-06	12.4561	2.5000E-08	14.3691	1.1000E-02	14.3004	1.0200E+03	**16.9059**	2.4500E+00	14.6542	3.6000E-15	14.6012	2.0300E+02
	4	**20.7013**	1.6480E-04	20.1531	1.5500E-03	20.1528	1.0500E-02	20.0639	1.3200E+02	20.1756	6.5600E-08	17.1663	6.4000E+01	20.1531	5.0200E-13	20.1234	3.9200E-01
	5	**21.2249**	3.8488E-02	20.6629	3.6200E-01	20.6417	6.0100E-02	20.5698	3.2000E+01	20.6621	9.0600E-15	20.6629	3.2500E+03	20.6608	2.3200E+01	20.5308	9.0000E-01
Test 5	2	**16.1870**	8.5162E-16	15.7584	8.0100E-14	13.7574	4.6100E-15	15.6588	9.0100E-15	15.7533	3.6000E-15	13.6633	4.5000E+00	15.7584	9.0100E-15	15.7584	1.8400E-06
	3	**19.3218**	2.8281E-14	18.8102	2.6600E-12	18.7322	2.0600E-10	18.8102	5.1600E-01	18.8384	7.0400E-10	18.8584	5.2300E+00	18.8102	3.6000E-15	18.8102	2.5400E-04
	4	**21.0439**	4.8163E-06	20.4867	4.5300E-04	19.2899	3.9700E-01	20.3878	4.6900E-02	18.3942	1.4400E+00	14.1232	5.2200E+00	20.3208	6.0900E-14	18.8102	1.7400E-02
	5	**20.7689**	3.8700E-02	20.2189	3.6400E+00	19.2089	3.6400E+01	20.0799	1.9400E-16	18.2268	3.1400E+02	14.2814	5.4100E-15	20.5404	5.1200E-02	20.4868	3.0100E-16
Test 6	2	13.2961	3.8275E-16	**13.7299**	3.6000E-15	11.3533	3.6000E-15	13.5298	3.6000E-15	13.5298	5.4100E-15	12.1402	2.8700E+00	13.5298	3.6000E-15	13.6298	3.9900E-02
	3	16.7287	7.9102E-02	17.0229	7.4400E-01	10.6508	3.0900E-04	16.8065	9.5800E-01	16.8065	1.0900E-03	**17.3447**	3.0900E+00	17.1017	6.4000E-03	17.0017	4.1200E-12
	4	**19.6562**	2.1902E-03	19.1357	2.0600E-02	13.6031	3.2200E-02	18.9026	2.8300E-12	18.9026	3.2100E-02	17.6229	3.0600E+01	19.0357	6.6700E-01	19.1125	3.0400E-06
	5	21.1709	2.4985E-01	20.6103	2.3500E+00	12.6928	1.3400E+02	20.7048	2.7700E+02	20.6103	2.8900E-01	**21.7048**	3.9100E-07	20.2447	6.8100E-06	20.3441	5.6300E-13
Test 7	2	**14.9932**	5.5605E-15	14.5962	5.2300E-14	14.4886	1.7300E-02	14.5962	3.5800E-02	14.5962	5.4100E-15	10.1415	7.2800E-01	14.6310	8.2000E-03	14.6213	2.0800E-04
	3	17.6125	3.3384E-15	17.1461	3.1400E-14	9.4044	2.4300E-10	17.1979	1.0800E-01	17.1461	3.4200E-08	**17.8925**	3.1800E+00	17.4714	3.6000E-15	17.4716	2.6900E-12
	4	19.6647	5.3373E-03	19.1440	5.0200E-02	14.8254	1.9200E-04	17.1461	2.8300E-01	19.1447	3.1200E-08	**17.9647**	3.6100E+00	19.4588	1.0600E-01	19.6587	3.0900E-15
	5	21.7843	5.3373E-01	**21.8975**	5.0200E+00	9.0705	3.1200E-01	21.1875	2.6900E-10	21.1341	5.0900E-02	21.2075	1.0900E+01	20.9991	3.4200E-08	20.5951	2.6900E-12
Test 8	2	**15.6964**	2.0094E-14	15.2029	1.8900E-13	15.1958	1.8000E-15	15.2029	1.2900E-04	15.2028	1.8000E-15	12.7136	5.6900E-10	15.2023	2.4500E-06	15.5967	6.0900E-10
	3	**19.0008**	1.5097E-02	18.4977	1.4200E-02	13.0695	1.0000E-01	18.4977	7.3200E-01	18.4987	2.6900E-12	15.1063	3.2800E+00	18.4977	0.0000E+00	18.0481	3.4200E-08
	4	**21.7998**	4.6887E-04	21.0278	4.4100E-02	11.8816	2.4200E-06	21.0279	3.0200E-18	21.0279	2.6200E+00	16.4222	3.4200E+00	21.0484	3.8900E-02	21.6258	1.0400E-01
	5	23.4708	3.7850E-04	22.8493	3.5600E+01	12.3141	1.2000E+00	21.0654	3.4200E-08	22.8493	3.0200E-06	**23.7483**	6.2200E-02	22.8258	2.1200E-01	15.4654	3.9400E-05
Test 9	2	15.3868	1.6267E-13	14.9794	1.5300E-13	10.7742	2.6900E-12	14.2865	6.0200E-06	10.8308	1.8000E-15	**17.5018**	6.8900E-02	14.4657	1.2600E-14	13.7461	5.3100E+00
	3	16.0658	2.0732E-12	**17.8962**	1.9500E-12	12.2133	1.1500E-04	14.2865	9.3000E-02	14.2865	6.0200E-02	17.8029	2.4400E+00	17.8462	3.8900E-02	15.8914	6.2200E-02
	4	19.1575	2.2752E-04	19.6237	2.1400E-04	15.068	3.9800E-05	15.9906	9.1500E-02	15.9906	6.8900E-02	**20.1125**	2.7900E+00	15.9906	5.1100E-14	19.5751	2.3200E-03
	5	**19.9007**	3.6787E-03	19.3737	3.4600E-01	12.2638	3.1000E-02	19.3733	2.6200E+00	19.3731	2.9600E+01	19.3737	3.0200E-13	19.1750	5.6100E-04	9.8521	1.9500E-12
Test 10	2	**16.8790**	5.4330E-15	12.2449	5.1100E-14	12.1690	3.0100E-12	12.2447	5.4100E-15	10.8308	3.8900E-15	12.2449	1.9500E-12	9.9525	6.8900E-02	16.8782	6.2900E-02
	3	**17.3375**	2.2752E-11	16.8784	2.1400E-10	16.8809	5.3200E-14	16.8784	3.4200E-04	16.8784	2.5400E-01	16.4935	3.4200E-08	16.8784	1.9500E-12	16.2132	2.4600E-10
	4	**20.6547**	6.5387E-05	20.1078	6.1500E-04	19.9473	2.5400E-01	20.1075	8.7300E-01	20.1078	2.9200E-06	16.7673	2.6200E+00	19.2037	3.4200E-04	19.2046	1.8100E-07
	5	20.7243	7.3254E-03	21.9716	6.8900E-02	18.4898	2.3400E+00	20.1075	3.4200E-08	**22.0537**	2.0200E-14	16.9775	6.2200E-02	21.8813	1.0900E+00	20.8812	6.8700E-09

Significant values are in bold.

**Table 20:** Mean and STD values of FSIM results obtained by kapur’s method.

Test Image	nTh	IHBO		HBO		SSA		MFO		GWO		SCA		HS		EMO	
		Mean	STD	Mean	STD	Mean	STD	Mean	STD	Mean	STD	Mean	STD	Mean	STD	Mean	STD
Test 1	2	**0.7647**	3.6969E-04	0.7327	3.4000E-03	0.6624	3.3800E-16	0.7327	3.3800E-16	0.7327	0.0000E+00	0.6322	3.5800E-03	0.6739	2.2300E-02	0.6327	2.1600E-02
	3	**0.7794**	2.7414E-04	0.7468	6.2000E-03	0.6615	2.2500E-16	0.7327	9.8000E-03	0.7479	6.5000E-03	0.2468	6.4900E-02	0.6911	2.2500E-16	0.6327	5.3000E-03
	4	**0.8524**	1.3048E-03	0.8167	1.2000E-03	0.6987	4.5100E-16	0.7485	1.2100E-03	0.8165	2.9000E-03	0.5167	6.9600E-02	0.7621	4.0000E-04	0.6486	5.7000E-03
	5	**0.8643**	2.0659E-03	0.8591	1.9000E-03	0.8224	2.3000E-03	0.8157	4.1000E-03	0.8319	1.9800E-02	0.8552	7.4200E-02	0.8174	4.8000E-03	0.7157	4.9000E-03
Test 2	2	0.7220	1.0873E-04	**0.7364**	1.0000E-04	0.6296	4.5100E-16	0.6684	4.5100E-16	0.7353	4.5100E-16	0.7351	2.9100E-02	0.7243	4.5100E-16	0.6689	2.0000E-04
	3	0.7281	2.4465E-03	0.7364	2.2500E-02	0.7341	2.7000E-02	0.7353	2.7200E-02	0.7364	4.3000E-03	0.7362	8.1300E-02	**0.7791**	2.1800E-02	0.7353	2.7000E-02
	4	**0.8647**	2.2073E-04	0.8477	2.0300E-02	0.8477	5.8100E-05	0.8108	7.1900E-03	0.8101	9.6000E-03	0.8577	8.7000E-02	0.7686	1.6000E-02	0.7108	2.5000E-03
	5	0.8698	2.2834E-05	0.8469	2.1000E-03	0.6174	4.0000E-03	0.8108	6.8000E-03	0.8265	1.0200E-02	**0.8709**	8.5200E-02	0.8173	6.0000E-03	0.8108	9.2000E-03
Test 3	2	0.8530	2.7183E-05	0.8556	2.5000E-03	0.6437	4.5100E-16	0.7142	4.5100E-16	0.8557	5.6300E-16	0.8456	1.0500E-01	**0.8651**	4.5100E-16	0.7142	3.0000E-04
	3	0.9030	3.1315E-04	0.8939	2.8800E-02	0.8945	9.6000E-03	0.855	9.6900E-06	0.8938	1.3000E-03	0.8639	1.4100E-01	**0.9067**	4.5100E-16	0.8254	4.0000E-04
	4	**0.9378**	1.6310E-05	0.9273	1.5000E-03	0.9213	1.0400E-02	0.8942	1.6000E-03	0.9177	2.7200E-02	0.9362	1.8050E-01	0.9253	8.9000E-03	0.8942	4.3000E-03
	5	**0.9559**	1.0873E-04	0.9446	1.0000E-03	0.9213	8.6000E-03	0.9178	1.3000E-03	0.9406	2.2800E-02	0.9432	1.7850E-01	0.8857	1.6200E-02	0.9078	6.7000E-03
Test 4	2	**0.7067**	1.7397E-04	0.6771	1.6000E-03	0.6693	0.0000E+00	0.5968	2.1500E-04	0.6771	0.0000E+00	0.6723	1.7500E-02	0.6136	0.0000E+00	0.5468	2.9000E-03
	3	0.7771	5.3279E-04	**0.8010**	4.9000E-03	0.6494	5.6300E-16	0.6771	1.1400E-03	0.8002	4.0000E-03	0.8004	6.9100E-02	0.7994	5.6300E-16	0.5771	1.7000E-03
	4	0.8146	3.6112E-05	0.8397	7.0000E-04	0.6916	8.0000E-04	0.8012	5.8800E-04	0.8383	4.8000E-03	0.8187	8.3800E-02	**0.8414**	5.0000E-04	0.8012	1.7000E-03
	5	**0.8863**	6.3065E-04	0.8684	5.8000E-03	0.6801	1.4200E-02	0.8658	3.8600E-03	0.8622	9.3000E-03	0.8662	1.0800E-01	0.8617	1.6600E-02	0.8558	1.1800E-02
Test 5	2	0.7849	4.9038E-17	0.7904	4.5100E-16	0.5794	4.5100E-16	0.7308	4.5100E-16	0.7904	3.3800E-16	0.6423	1.0600E-01	**0.7913**	4.5100E-16	0.7413	7.0000E-04
	3	0.8608	6.8501E-04	0.8602	6.3000E-03	0.7542	5.6300E-16	0.8602	1.1700E-02	0.8602	2.8000E-03	**0.8612**	1.1900E-01	0.8607	5.6300E-16	0.8407	3.0000E-04
	4	**0.9070**	6.5239E-05	0.8978	6.0000E-04	0.7352	1.8700E-02	0.8603	5.3700E-04	0.8973	4.2000E-03	0.8875	1.1900E-01	0.8991	1.8900E-02	0.8607	1.0000E-02
	5	0.9091	2.0007E-03	0.8902	1.8400E-02	0.7522	8.4000E-03	**0.9098**	1.5600E-02	0.8682	1.8200E-02	0.8856	1.2820E-01	0.8374	7.5000E-03	0.8484	9.2000E-03
Test 6	2	**0.7958**	4.1318E-04	0.7625	3.8000E-03	0.7591	3.3800E-16	0.7625	3.3800E-16	0.7625	5.6300E-16	0.4562	5.3500E-02	0.7625	3.3800E-16	0.7127	5.2300E-04
	3	0.8468	3.9144E-04	**0.8497**	3.6000E-03	0.5891	3.9600E-04	0.8476	2.6600E-02	0.8473	1.5000E-03	**0.8497**	6.3700E-02	0.8476	1.6900E-04	0.7327	6.0000E-04
	4	0.8986	5.1104E-04	0.8998	4.7000E-03	0.7352	7.5000E-03	**0.8999**	2.5700E-04	0.8997	1.4800E-02	0.8112	6.6500E-02	0.8975	1.6000E-02	0.7489	1.8700E-02
	5	0.9245	1.1961E-04	0.9257	1.1000E-03	0.6903	3.6000E-03	0.9246	9.4700E-03	**0.9265**	1.5600E-02	0.8425	7.1400E-02	0.9258	4.5000E-03	0.8161	1.2500E-02
Test 7	2	0.7482	4.1216E-17	0.7169	5.6300E-16	0.6273	5.7700E-03	0.7168	1.9700E-03	0.7166	3.3800E-16	0.7342	4.6800E-02	0.7276	2.7400E-03	0.6681	4.4000E-03
	3	0.7941	5.5453E-04	0.7992	5.1000E-03	0.7089	1.0700E-03	0.7993	2.2200E-03	**0.7993**	5.3000E-03	0.7345	1.0900E-01	**0.7993**	4.5100E-16	0.7453	4.0000E-03
	4	**0.8918**	3.0890E-05	0.8545	5.6000E-03	0.7441	4.0000E-03	0.8545	3.2900E-03	0.8544	8.5000E-03	0.8137	1.2100E-01	0.8537	2.0400E-03	0.8008	5.4000E-03
	5	**0.9145**	2.6096E-05	0.9050	2.4000E-03	0.6529	3.3000E-03	0.9028	1.2200E-02	0.9005	1.2600E-02	0.8426	1.3010E-01	0.8904	5.5000E-03	0.8108	2.8000E-03
Test 8	2	0.6907	1.4135E-03	0.7152	1.3000E-03	0.6758	1.1300E-16	0.7153	2.2600E-03	0.7153	0.0000E+00	**0.8425**	1.0700E-03	0.7153	4.5400E-02	0.5042	5.2100E-02
	3	0.7956	6.6327E-03	0.8238	6.1000E-03	0.8210	4.5100E-16	0.8239	5.7500E-03	0.8239	9.9000E-03	**0.8923**	7.7300E-02	0.8239	4.5100E-16	0.6553	2.7000E-03
	4	0.8273	1.7397E-03	0.8885	1.6000E-03	0.8868	6.0000E-04	0.8886	1.5800E-02	0.8905	6.3000E-03	**0.9125**	9.0200E-02	0.8886	1.0000E-03	0.7942	8.2000E-03
	5	0.9297	2.9901E-04	0.8908	2.7500E-02	0.6242	2.2600E-02	0.8906	2.4600E-02	0.8918	3.2000E-02	**0.9498**	9.0700E-02	0.8981	2.4100E-02	0.8178	1.6500E-02
Test 9	2	0.6760	2.4465E-18	0.6477	2.2500E-16	0.6443	5.1900E-03	**0.7332**	4.1500E-03	0.6471	1.1300E-16	0.6123	5.4700E-02	0.6459	2.2500E-16	0.5468	6.9000E-03
	3	**0.8360**	3.6771E-05	0.8010	8.9000E-03	0.7318	5.0000E-04	0.7332	1.7000E-03	0.7332	8.1000E-03	0.7312	1.0500E-01	0.6484	3.6500E-04	0.6771	3.9000E-03
	4	0.7899	3.4366E-06	0.7568	5.0000E-04	0.8123	1.0000E-04	0.7554	1.0000E-03	0.7554	5.1000E-03	**0.8353**	1.1970E-01	0.7461	6.4800E-04	0.7012	1.7000E-03
	5	0.8083	1.4135E-04	0.8344	1.3000E-03	0.6201	1.1000E-03	0.8341	1.7000E-03	**0.8545**	5.6000E-03	0.8468	1.2170E-01	0.8336	1.4000E-03	0.8358	2.2000E-03
Test 10	2	**0.7673**	1.1961E-03	0.6476	1.1000E-03	0.6467	2.0200E-02	0.6477	1.1300E-16	0.6477	1.1300E-16	0.7123	3.3200E-04	0.6477	2.7800E-02	0.7108	1.2800E-02
	3	0.8721	5.4366E-04	0.8452	5.0000E-04	0.7455	3.7000E-03	0.7459	8.4000E-04	0.7459	1.8200E-02	**0.8821**	4.1000E-02	0.7459	3.2700E-02	0.7602	4.2600E-02
	4	**0.9468**	2.6639E-02	0.9072	2.4500E-02	0.7264	9.3000E-03	0.8372	2.4900E-02	0.8372	1.0880E-02	0.8978	9.3200E-02	0.8279	1.8660E-02	0.8603	2.5600E-02
	5	**0.9535**	4.7859E-03	0.9136	9.0000E-03	**0.9535**	6.5000E-03	0.8898	7.0400E-03	0.8384	2.8600E-02	0.8902	1.4100E-01	0.8888	1.1700E-02	0.8778	1.9700E-02

Significant values are in bold.

**Table 21:** Mean and STD values of SSIM results obtained by Kapur’s method.

Test Image	nTh	IHBO		HBO		SSA		MFO		GWO		SCA		HS		EMO	
		Mean	Std	Mean	Std	Mean	Std	Mean	Std	Mean	Std	Mean	Std	Mean	Std	Mean	Std
Test 1	2	0.5218	3.9233E-04	0.5044	3.8000E-03	0.5034	3.3800E-16	0.5017	3.3800E-16	**0.5838**	5.6300E-17	0.4799	3.5400E-03	0.5191	2.1700E-02	0.5017	2.0400E-02
	3	0.6501	6.5044E-04	0.6284	6.3000E-03	0.5052	5.6300E-16	0.6687	6.1600E-03	**0.7047**	9.4000E-03	0.6158	1.5400E-01	0.6266	5.6300E-16	0.5687	6.3000E-03
	4	**0.8299**	3.2272E-05	0.8022	7.0000E-04	0.5991	6.7600E-16	0.8005	8.1200E-04	0.7637	9.6000E-03	0.7279	1.5200E-01	0.7195	4.0000E-04	0.6005	3.5000E-03
	5	0.8633	1.1357E-04	**0.8786**	1.1000E-03	0.8741	1.4000E-03	0.8741	2.5000E-03	0.7438	1.5400E-02	0.8139	1.5850E-01	0.7613	4.2000E-03	0.6741	2.7000E-03
Test 2	2	**0.6319**	1.3422E-04	0.5448	1.3000E-03	0.4549	1.1300E-16	0.4923	1.1300E-16	0.5279	5.6300E-17	0.1283	2.1900E-02	**0.6319**	1.1300E-16	0.4923	9.7100E-05
	3	0.5424	3.9233E-03	0.5520	3.8000E-03	0.6239	1.0500E-02	**0.7448**	1.0600E-02	0.6305	8.3000E-03	0.6647	1.9600E-01	0.6601	8.5000E-03	0.6448	1.4500E-02
	4	**0.8166**	3.9233E-03	0.6052	3.8000E-03	0.7796	4.0000E-04	**0.8166**	8.5900E-03	0.6187	1.6700E-02	0.7702	1.6000E-01	0.7013	1.7200E-02	0.7166	5.1000E-03
	5	0.6884	1.3422E-03	0.6654	1.3000E-03	0.3887	6.8000E-03	0.8166	8.5000E-03	0.6381	1.3800E-02	**0.8264**	1.2340E-01	0.7202	2.8300E-02	0.8166	1.0500E-02
Test 3	2	0.6555	5.2655E-04	0.6336	5.1000E-03	0.4136	4.5100E-16	0.6262	4.5100E-16	**0.7534**	3.3800E-16	0.6436	1.2400E-01	0.6448	4.5100E-16	0.6262	2.2100E-05
	3	0.8120	1.6519E-04	0.7269	1.6000E-03	**0.8347**	1.8100E-02	0.8139	1.4300E-04	0.6335	7.0000E-04	0.7669	1.6400E-01	0.7433	7.8900E-16	0.8139	3.0000E-04
	4	**0.8924**	1.6519E-04	0.7659	1.6000E-03	0.8737	1.3400E-02	0.8894	1.6000E-03	0.8700	4.4100E-02	0.8759	2.0700E-01	0.7838	1.1200E-02	0.8794	5.9000E-03
	5	**0.9290**	1.0325E-04	0.8692	1.0000E-03	0.8692	7.8000E-03	0.9185	9.0700E-04	0.9212	2.5000E-02	0.6592	2.0800E-01	0.7147	1.4500E-02	0.9185	9.7000E-03
Test 4	2	**0.5555**	3.0974E-05	0.5369	3.0000E-04	0.5197	1.1300E-16	0.1315	4.0000E-04	0.5316	0.0000E+00	0.5549	8.1100E-03	0.3546	1.1300E-16	0.1115	4.7000E-03
	3	**0.6699**	3.0974E-05	0.6282	3.0000E-04	0.2821	3.3800E-16	0.6639	3.0800E-03	0.6148	5.9000E-03	0.6287	7.2700E-02	0.6247	3.3800E-16	0.6639	6.7000E-03
	4	**0.7790**	1.3422E-04	0.6853	1.3000E-03	0.3141	1.1000E-03	0.7760	1.3000E-03	0.6803	1.2900E-02	0.6854	9.6500E-02	0.6879	9.0000E-04	0.6768	3.5000E-03
	5	**0.8456**	9.2921E-04	0.8367	9.0000E-03	0.3995	1.9500E-02	0.8347	9.1800E-03	0.7054	2.2200E-02	0.8359	1.2600E-01	0.7081	2.1800E-02	0.8247	1.7100E-02
Test 5	2	0.7754	4.6563E-17	0.7495	4.5100E-16	0.3785	4.5100E-16	**0.8224**	4.5100E-16	0.8104	3.3800E-16	0.7595	1.9600E-01	0.7478	4.5100E-16	0.7468	9.0000E-04
	3	0.8304	6.1888E-04	0.8027	8.9000E-03	0.7685	7.8900E-16	0.9226	7.7500E-03	0.8675	3.1000E-03	**0.9236**	1.9600E-01	0.8005	7.8900E-16	0.8105	4.0000E-04
	4	0.9062	5.1947E-05	**0.9406**	6.0000E-04	0.7058	1.1200E-02	0.9226	1.9300E-03	0.8357	3.6000E-03	**0.9406**	1.9900E-01	0.8361	1.1400E-02	0.8215	5.5000E-03
	5	0.9161	4.0531E-04	**0.9508**	7.8000E-03	0.7664	4.2000E-03	0.9452	7.0000E-03	0.7976	1.5800E-02	**0.9508**	2.1990E-01	0.7794	4.7000E-03	0.8348	3.3000E-03
Test 6	2	0.4244	5.1623E-05	0.4102	5.0000E-04	0.5247	4.5100E-16	0.5875	4.5100E-16	**0.5879**	1.1300E-16	0.5154	8.4500E-02	0.4103	4.5100E-16	0.5017	1.3000E-03
	3	**0.7983**	1.5900E-03	0.6044	1.5400E-02	0.3652	4.7000E-04	0.7874	2.2000E-02	0.7883	3.8000E-03	0.6454	8.8400E-02	0.5901	1.0000E-04	0.6487	7.0000E-04
	4	**0.8693**	7.2272E-04	0.7087	7.0000E-04	0.6089	9.5700E-03	0.8660	4.7800E-04	0.8601	1.3300E-02	0.8022	9.9500E-02	0.7134	1.5500E-02	0.8005	2.4000E-02
	5	0.8921	5.1623E-04	0.7656	5.0000E-04	0.5617	7.4000E-03	0.9062	2.0500E-02	0.8794	1.6900E-02	**0.9146**	1.1500E-01	0.7656	7.3000E-03	0.8441	1.1000E-02
Test 7	2	0.4345	5.2655E-04	0.4831	5.1000E-03	0.4845	4.9000E-03	0.6839	1.7000E-03	0.5779	3.3800E-16	**0.5442**	3.3600E-02	0.4942	2.3000E-03	0.4923	3.3000E-03
	3	0.6360	7.8466E-04	0.6148	7.6000E-03	0.6306	3.0000E-04	**0.8244**	6.3600E-04	0.7235	4.1000E-03	0.5532	1.5400E-01	0.6148	6.7600E-16	0.7148	2.5000E-03
	4	0.6925	1.5487E-04	0.6694	1.5000E-03	0.6591	4.4000E-03	**0.8634**	5.0900E-03	0.7874	6.5000E-03	0.6052	1.6000E-01	0.6702	2.4900E-03	0.6066	5.4000E-03
	5	0.7525	1.5487E-04	0.7274	1.5000E-03	0.5411	2.1000E-03	0.9054	1.0600E-02	**0.9067**	1.6500E-02	0.6124	1.6300E-01	0.7097	3.6000E-03	0.4136	1.7000E-03
Test 8	2	**0.6538**	1.0325E-05	0.3613	1.0000E-04	0.4099	1.1300E-16	0.6104	6.7400E-03	0.4361	1.7600E-18	0.6336	2.8200E-03	0.3613	9.8700E-02	0.6262	9.9400E-02
	3	**0.7617**	4.0266E-04	0.4849	3.9000E-03	0.5846	5.6300E-16	0.7536	4.2300E-02	0.5909	2.8700E-02	0.5532	1.1900E-01	0.7648	5.6300E-16	0.7148	6.9000E-03
	4	**0.8486**	5.8850E-04	0.5786	5.7000E-03	0.6865	1.0000E-04	0.8375	1.1200E-02	0.6964	1.6200E-02	0.6052	1.1630E-01	0.6702	7.5000E-04	0.6066	4.4000E-03
	5	**0.9023**	1.7448E-03	0.5822	1.6900E-02	0.3601	1.4100E-02	0.8388	1.5800E-02	0.6972	2.8600E-02	0.6124	1.1040E-01	0.7097	1.3600E-02	0.4136	9.1000E-03
Test 9	2	0.6980	4.1239E-03	0.5561	6.9000E-03	0.3421	1.6400E-02	0.6373	1.3100E-02	0.3466	1.1300E-16	0.6124	5.9800E-02	**0.7097**	0.0000E+00	0.4136	3.8400E-02
	3	**0.7178**	5.2979E-03	0.6282	6.1000E-03	0.5571	1.3000E-03	0.6373	3.6200E-03	0.5637	3.6600E-02	0.6336	1.2200E-01	0.7613	1.3900E-03	0.6262	9.1000E-03
	4	0.7246	1.1357E-03	0.64562	1.1000E-03	0.7357	6.0000E-04	0.7556	2.7800E-03	0.6727	1.8900E-02	0.6785	1.4500E-01	**0.7850**	2.1800E-03	0.6339	3.8000E-03
	5	0.7940	2.8909E-04	0.6708	2.8000E-03	0.4719	5.2000E-03	0.8719	4.7500E-03	**0.8799**	1.2300E-02	0.7642	1.4800E-01	0.8786	5.6000E-03	0.6694	5.6000E-03
Test 10	2	**0.8690**	2.5811E-04	0.5113	2.5000E-03	0.4771	5.4800E-02	0.5898	2.2500E-16	0.4826	2.2500E-16	0.8681	2.1500E-04	0.6029	7.5100E-02	0.8185	2.4300E-02
	3	**0.8785**	8.2596E-04	0.6365	8.0000E-03	0.7206	2.0000E-02	0.8337	6.2600E-03	0.7226	4.9100E-02	**0.8785**	4.5700E-02	0.3678	7.4300E-02	0.1315	6.7800E-02
	4	0.8837	2.3127E-02	0.7092	2.2400E-02	0.6881	8.8000E-03	**0.8996**	2.6300E-02	0.8199	6.5700E-02	0.4235	1.3000E-01	0.7005	3.1000E-02	0.6639	3.3900E-02
	5	**0.9360**	1.2286E-02	0.7514	1.1900E-02	0.8106	1.9000E-02	0.9321	2.0600E-02	0.8197	5.7000E-02	0.6853	2.1820E-01	0.5919	1.3700E-02	0.6764	3.1200E-02

Significant values are in bold.

**Table 22:** Comparison of the *p*-values obtained through the Wilcoxon signed-rank test between the pairs of IHBO vs HBO, IHBO vs SSA, IHBO vs MFO, IHBO vs GWO, IHBO vs SCA, IHBO vs HS, and IHBO vs EMO for fitness results using Kapur’s method.

Test Image	nTh	HBO	SSA	MFO	GWO	SCA	HS	EMO
Test 1	2	1.413E-01	1.570E-01	1.600E-01	1.570E-01	9.640E-08	1.570E-01	1.570E-01
	3	2.960E-03	3.290E-02	2.870E-01	2.390E-01	1.870E-13	2.970E-03	1.790E-15
	4	7.495E-01	8.330E-01	4.570E-01	9.120E-01	4.340E-13	6.210E-07	1.170E-05
	5	1.152E-02	1.280E-03	3.620E-02	6.830E-03	1.570E-12	1.250E-04	1.710E-05
Test 2	2	3.005E-01	3.340E-01	3.410E-01	3.430E-01	6.720E-06	3.380E-01	3.210E-01
	3	1.988E-03	2.210E-02	7.540E-01	2.210E-02	8.330E-14	2.210E-02	7.920E-16
	4	1.188E-01	1.320E-02	1.440E-01	2.930E-03	4.400E-13	1.480E-06	1.680E-04
	5	2.411E-04	2.680E-04	8.390E-03	8.110E-09	6.480E-13	1.170E-07	2.630E-01
Test 3	2	3.770E-03	4.190E-02	4.180E-02	4.190E-02	1.870E-04	4.190E-02	4.220E-10
	3	2.132E-02	2.370E-03	6.310E-01	1.780E-04	6.670E-13	1.810E-04	8.840E-04
	4	1.134E-01	1.260E-02	5.300E-01	4.670E-07	8.680E-14	3.230E-14	1.010E+01
	5	4.040E-04	4.490E-05	1.620E-01	4.570E-09	6.550E-13	7.260E-12	4.570E-05
Test 4	2	NaN	NaN	NaN	1.630E-05	1.680E-05	NaN	1.120E-16
	3	2.186E-02	2.430E-02	6.490E-01	5.870E-03	7.870E-12	5.920E-03	1.380E-15
	4	5.191E-01	5.770E-01	1.640E-01	3.460E-04	4.700E-12	6.480E-06	3.080E-01
	5	6.901E-03	7.670E-03	6.550E-06	1.170E-07	8.140E-11	5.510E-11	4.590E-11
Test 5	2	2.907E-01	3.231E-01	3.290E-01	4.520E-06	2.590E-07	4.120E-01	1.100E-16
	3	2.402E-02	2.670E-02	3.620E-01	3.670E-05	5.720E-13	3.670E-05	1.400E-02
	4	1.125E-07	1.250E-07	8.290E-05	1.910E-11	4.400E-10	7.400E-13	2.700E-09
	5	7.171E-01	7.970E-01	1.960E-01	1.180E-02	5.140E-13	4.890E-05	4.310E-01
Test 6	2	1.825E-05	NaN	3.310E-01	NaN	3.290E-01	NaN	7.660E-15
	3	5.299E-03	5.890E-03	2.340E-02	1.690E-01	1.510E-13	5.910E-03	1.470E-15
	4	4.175E-01	4.640E-01	3.340E-02	9.190E-03	6.230E-13	1.480E-03	3.560E-03
	5	5.578E-01	6.200E-01	4.500E-01	1.700E-03	5.720E-13	4.140E-08	2.300E-07
Test 7	2	1.988E-01	2.210E-02	2.210E-02	2.210E-02	2.120E-04	2.210E-02	2.210E-02
	3	6.739E-03	7.490E-04	3.160E-03	3.470E-01	2.870E-13	7.490E-04	2.890E-15
	4	5.290E-01	5.880E-01	2.990E-01	1.290E-01	1.850E-13	7.530E-04	7.270E-04
	5	2.627E-01	2.920E-01	1.900E-02	4.790E-03	8.110E-13	1.340E-09	1.250E-07
Test 8	2	1.467E-01	1.630E-01	1.620E-01	1.620E-01	4.480E-05	1.620E-01	1.620E-01
	3	1.359E-03	1.510E-03	1.510E-03	5.910E-03	2.650E-13	1.510E-03	1.190E-04
	4	6.757E-01	7.510E-01	1.410E-02	9.850E-01	4.420E-13	4.580E-05	8.300E-01
	5	2.564E-01	2.850E-01	2.760E-04	7.810E-01	9.130E-13	2.650E-12	1.120E-06
Test 9	2	5.560E-04	6.180E-04	4.200E-02	6.180E-04	1.640E-05	6.170E-04	6.170E-04
	3	6.739E-04	7.490E-04	4.370E-01	3.100E-03	6.510E-13	7.500E-04	2.910E-15
	4	5.236E-07	5.820E-07	6.130E-02	6.850E-07	5.950E-13	2.060E-09	5.100E-14
	5	8.601E-01	9.560E-01	4.240E-04	3.700E-07	4.150E-13	4.240E-02	4.230E-02
Test 10	2	NaN	4.240E-02	NaN	NaN	2.380E-04	NaN	NaN
	3	3.842E-02	4.270E-02	1.650E-01	6.590E-01	1.900E-13	4.200E-02	3.910E-10
	4	7.908E-01	8.790E-02	2.400E-04	1.370E-02	4.790E-13	1.480E-06	6.880E-14
	5	3.239E-05	3.600E-06	4.600E-03	4.510E-09	1.890E-12	9.110E-12	1.600E-08

### Human participants or animals

This article does not contain any studies with human participants or animals performed by any of the authors.

## Conclusions and future works

Image segmentation is the most substantial pivotal phase that should be performed for image analysis and understanding. To handle this growing challenge, different methods using MTH, including feature-based, threshold-based, and region-based segmentation, have been implemented. The most common technique used to perform and analyze image segmentation is threshold-based segmentation. This paper presented an improved variant of the Heap-based optimizer (HBO) called IHBO. The effectiveness of the proposed IHBO was estimated using the functions in the CEC’2020 benchmark functions, however, the proposed algorithm superiority on the competing algorithms regarding various statistical metrics. In addition, IHBO was applied to image segmentation using objective functions such as the Otsu and Kapur methods. The main target of IHBO is to determine the best thresholds that maximize the Otsu and Kapur methods. The IHBO was implemented on a set of test images with different characteristics, and the results were compared against seven well-known metaheuristic algorithms including the original HBO algorithm, SSA, MFO, GWO, SCA, HS, and EMO. The experimental results revealed that the IHBO algorithm outperformed all counterparts in terms of FSIM, SSIM, and PSNR. It should be noted that the IHBO results using the Otsu method provided better class variance in most metrics. However, when applying the Kapur method, the IHBO produced SSIM, FSIM, PSNR, and fitness values were better than those of all counterparts. The IHBO produced promising results because it preserved an effective balance between exploration and exploitation, and had the ability to avoid being trapped in local optima.

For future work, there are many research directions in this field, such as studying the performance of the IHBO algorithm on different datasets, and other real-world complex problems. In addition, future work can study the hybridization of the original HBO with other metaheuristic or machine learning algorithms to automate the search process for the optimal number of thresholds in a specific image.

## Data Availability

All data generated or analysed during this study are included in this published article^71,72^.
